# Epigenetics-targeted drugs: current paradigms and future challenges

**DOI:** 10.1038/s41392-024-02039-0

**Published:** 2024-11-26

**Authors:** Wanlin Dai, Xinbo Qiao, Yuanyuan Fang, Renhao Guo, Peng Bai, Shuang Liu, Tingting Li, Yutao Jiang, Shuang Wei, Zhijing Na, Xue Xiao, Da Li

**Affiliations:** 1 Center of Reproductive Medicine, Department of Obstetrics and Gynecology, Shengjing Hospital of China Medical University, Shenyang, China; 2 Department of Orthopedics, Shengjing Hospital of China Medical University, Shenyang, China; 3 Department of Forensic Genetics, West China School of Basic Medical Sciences & Forensic Medicine, Sichuan University, Chengdu, China; 4 Shenyang Maternity and Child Health Hospital, Shenyang, China; 5 Department of General Internal Medicine VIP Ward, Liaoning Cancer Hospital & Institute, Shenyang, China; 6 NHC Key Laboratory of Advanced Reproductive Medicine and Fertility (China Medical University), National Health Commission, Shenyang, China; 7 Department of Gynecology and Obstetrics, West China Second Hospital, Sichuan University, Chengdu, China; 8 Key Laboratory of Birth Defects and Related Diseases of Women and Children (Sichuan University), Ministry of Education, West China Second Hospital, Sichuan University, Chengdu, China; 9 Key Laboratory of Reproductive Dysfunction Diseases and Fertility Remodeling of Liaoning Province, Shenyang, China

**Keywords:** Epigenetics, Molecular medicine

## Abstract

Epigenetics governs a chromatin state regulatory system through five key mechanisms: DNA modification, histone modification, RNA modification, chromatin remodeling, and non-coding RNA regulation. These mechanisms and their associated enzymes convey genetic information independently of DNA base sequences, playing essential roles in organismal development and homeostasis. Conversely, disruptions in epigenetic landscapes critically influence the pathogenesis of various human diseases. This understanding has laid a robust theoretical groundwork for developing drugs that target epigenetics-modifying enzymes in pathological conditions. Over the past two decades, a growing array of small molecule drugs targeting epigenetic enzymes such as DNA methyltransferase, histone deacetylase, isocitrate dehydrogenase, and enhancer of zeste homolog 2, have been thoroughly investigated and implemented as therapeutic options, particularly in oncology. Additionally, numerous epigenetics-targeted drugs are undergoing clinical trials, offering promising prospects for clinical benefits. This review delineates the roles of epigenetics in physiological and pathological contexts and underscores pioneering studies on the discovery and clinical implementation of epigenetics-targeted drugs. These include inhibitors, agonists, degraders, and multitarget agents, aiming to identify practical challenges and promising avenues for future research. Ultimately, this review aims to deepen the understanding of epigenetics-oriented therapeutic strategies and their further application in clinical settings.

## Introduction

**Fig. 1:**
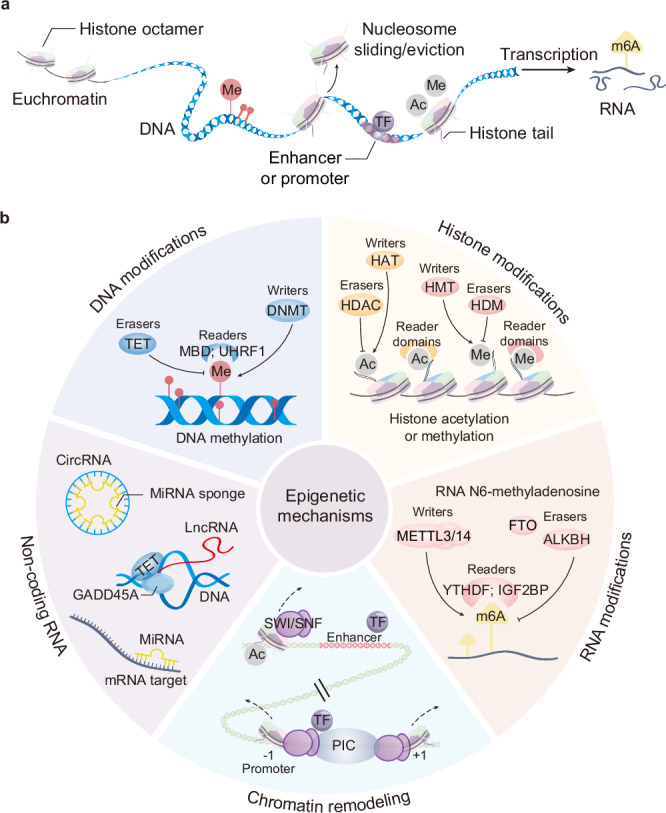
Epigenetic mechanisms and key examples of widely studied modifications and their modifying enzymes. **a** DNA modifications, histone modifications, RNA modifications, chromatin remodeling, and the regulation based on non-coding RNA constitute the core content of epigenetics, being responsible for passing on heritable variations of genetic information independently of the DNA sequence. **b** Epigenetic modifications are reversible progress catalyzed by functionally complementary modifying enzymes, which provide targets for disease therapeutics

From a historical perspective, the term “epigenetics” was first introduced by Conrad Waddington in 1942 to describe heritable changes in gene function that do not involve alterations to the DNA sequence, leading to changes in biological phenotypes. Following nearly a century of rigorous research, a diverse array of epigenetic-modifying enzymes has been identified, and the elucidation of distinct molecular mechanisms has established epigenetics as a robust discipline.^1^ Presently, epigenetics is defined as a chromatin state regulatory system comprised of five principal mechanisms: DNA modifications,^2^ histone modifications,^3^ RNA modifications,^4^ chromatin remodeling,^5^ and the regulation based on non-coding RNA (ncRNA).^6^ These mechanisms independently transmit genetic information from the DNA sequence, enabling the activation or repression of specific genome regions in response to physiological or pathological signals (Fig. 1).

Enzymes that regulate epigenetic modifications are categorized into “writers,” “erasers,” “readers,” and “remodelers” based on their functions.^7–9^ Writers modify specific bases or amino acids, whereas erasers remove these modifications, exerting reciprocal effects on gene expression. For instance, DNA methyltransferase (DNMT) catalyzes the addition of methyl groups to form 5-methylcytosine (m5C) in DNA bases,^10^ whereas the ten-eleven translocation (TET) enzymes initiate DNA demethylation, converting m5C into derivatives, such as 5-hydroxymethylcytosine (5hmC), 5-formylcytosine, and 5-carboxycytosine.^11^ Typically, genes expressed at higher levels exhibit lower methylation, whereas genes with lower expression levels tend to be more heavily methylated.^12^ Readers are proteins that contain specific motifs to recognize and bind these modifications, such as the methyl-CpG-binding domain (MBD) responsible for recognizing 5mC.^13^ These proteins influence chromatin status and recruit or collaborate with other enzymes to regulate gene expression.^13,14^ Remodelers are crucial in chromatin remodeling, moving or removing nucleosomes at vital regulatory elements like enhancers and promoters to modify chromatin accessibility.^15^ Furthermore, as unique epigenetic regulators distinct from epigenetic-modifying enzymes, ncRNAs directly bind to various genomic regions or specific RNA sequences to modulate gene expression.^16^ Variations in the given ncRNA may regulate the interactions or functions of its interactor partners, including proteins, RNAs, DNAs, and lipids, thereby influencing various biological cellular processes or pathological phenotypes.^17^

**Fig. 2:**
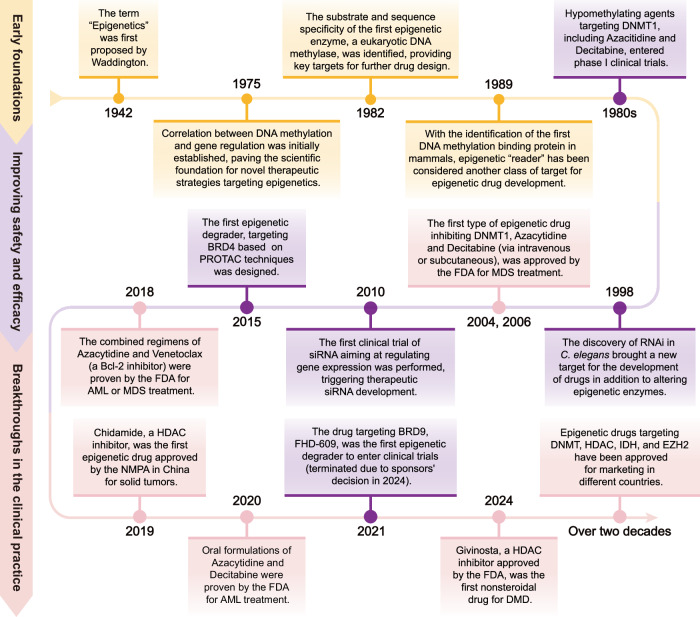
Timeline of major discoveries and advances in epigenetic research. The significant discoveries and advances are depicted in the illustrator and displayed as primarily “Early foundations” (yellow boxes) on the top, “Improving safety and efficacy” (purple boxes) in the middle, and “Breakthroughs in the clinical practice” (pink boxes) at the bottom

The discovery of functionally complementary epigenetic-modifying enzymes has underscored the reversibility of most known epigenetic modifications. This insight supports the development of strategies to modulate gene expression via targeted regulation of these enzymes, providing a strong theoretical basis for creating novel therapeutic approaches from an epigenetic perspective. To date, four categories of epigenetics-targeted drugs have received the Food and Drug Administration (FDA) approval for clinical use, with numerous clinical trials ongoing to refine their applications. A timeline of significant milestones in epigenetic research is depicted in Fig. 2.

Over the past few decades, numerous studies have underscored that abnormalities in the expression and function of epigenetic-regulating enzymes are crucial in the onset and progression of various diseases. Epigenetics-targeted drugs, therefore, have emerged as pivotal topics due to their significant physiological and pathological implications. The development of drug screening models rooted in epigenetic principles is anticipated to substantially expand therapeutic options in clinical settings. Moreover, advancements in epigenetic analysis and molecular modification techniques have accelerated the clinical adoption of these targeted drugs. Despite these developments, there remains a gap in comprehensive reviews that address epigenetic regulations in physiological and disease contexts and detail the latest advancements in drug development targeting these mechanisms. This review aims to fill that void by summarizing the current understanding of epigenetic regulations and clinical trials of targeted drugs, thereby outlining the future application of these promising agents. We begin with an overview of epigenetic mechanisms and their crucial roles in health and disease, followed by an in-depth discussion on the exploration and application of marketed epigenetic drugs. We then provide a systematic account of recent progress in developing potential therapeutic agents targeting various epigenetic enzymes, highlighting emerging research trends. Finally, we present the breakthroughs and challenges in epigenetic drugs, particularly the benefits of combining them with traditional therapies such as radiotherapy, chemotherapy, and targeted therapy, to underscore their potential in translational medicine.

## Biological and pathological roles of epigenetics

Epigenetic modifications are a fundamental mechanism regulating gene expression, crucial for various cellular functions. Dysregulated epigenetic regulators, whether overexpressed or underactive, compromise normal functions and contribute to disease onset. Thus, epigenetic modifications hold significant potential for disease treatment and biotechnological applications, driving the development of targeted therapeutic drugs.

### Epigenetics and early embryonic development

Epigenetic landscapes undergo substantial changes to ensure the coordinated progression of embryogenesis and subsequent development throughout an individual’s life.^18^ Mutations in epigenetic-modifying enzymes, whether heterozygous or hemizygous, are commonly associated with congenital conditions, such as Rubinstein-Taybi syndrome, linked to mutations in the cyclic adenosine monophosphate-responsive element-binding protein (CREB)-binding protein (CBP) and its paralog, E1A-binding protein (P300),^19^ immunodeficiency-centromeric instability-facial anomalies syndrome related to DNMT3B mutations,^20^ and Kabuki syndrome due to mutations in lysine methyltransferase 2D (KMT2D).^21^ DNA methylation reprogramming, a pivotal aspect of epigenetic modification in early embryonic stages, involves genome-wide removal of epigenetic marks through extensive DNA demethylation, followed by remethylation.^22^ This process, integral to mammalian development, has only been fully understood with the advent of whole-genome bisulfite sequencing, which allows for single-base resolution analysis of DNA methylation kinetics.^23,24^ Advances in precise assays for assessing DNA methylation at specific genetic loci have led to significant insights into these epigenomic reprogramming processes. This reprogramming results in global hypomethylation and significant loss of genetic memory, which is foundational for acquiring pluripotency and redetermining cell fate.^25^ Following fertilization, methylation patterns evolve progressively, enabling cells to differentiate and contribute to the development of various biological systems. The dynamic regulation of DNA methylation, including reprogramming, is indispensable for mammalian development and differentiation. Another vital mechanism, histone modification, plays a critical role during zygotic genome activation (ZGA), which involves the transition of the zygotic genome from a state of silence to active transcription.^26^ Notably, the de novo establishment of histone 3 lysine 14 acetylation (H3K14ac) and histone 3 lysine 9 trimethylation (H3K9me3) following fertilization is crucial for the timely activation of ZGA genes during development.^27,28^ The SWItch/Sucrose NonFermentable (SWI/SNF) complex also plays a significant role in the precise activation and repression of tissue-specific transcription factors, functioning as a chromatin remodeler that orchestrates the coordinated differentiation of multiple cell lineages during development.^29,30^ Additionally, the role of RNA modifications in embryo development is increasingly recognized and summarized.^31,32^ Recent studies indicate that deficiencies in methyltransferase-like proteins (METTLs) and their associated RNA N6-methyladenosine (m6A) levels can induce G1/S cell cycle arrest in hematopoietic stem and progenitor cells in model organisms.^33^ Furthermore, aberrant RNA modification patterns are integrated into the regulatory networks of other epigenetic mechanisms, such as histone deubiquitylation and DNA methylation, playing critical roles in nuclear reprogramming.^34,35^ Recently, preliminary evidence of ncRNAs being engaged in embryo development has been proposed according to the reported variation among ncRNAs contents during different stages of early embryonic development in mouse models.^36–38^ As an indicator of developmental competence, ncRNA plays an irreplaceable role in the continuous stages of pre-implantation development, embryo implantation, and post-implantation development.^39^ Aberrant levels of certain ncRNAs may disturb the transition of fertilized oocytes to pluripotent blastocysts, and may even affect the differentiation of epiblast stem cells.^40,41^ Notably, ncRNAs may also act as regulatory factors for other epigenetic mechanisms. For example, during mouse ZGA, the negative regulation of Dnmt3a/3b expression by microRNA-29b (miR-29b) helps maintain proper DNA demethylation to establish the imprinting of genes.^42^ However, considering that most of the current understanding has only been validated in animal models, much work is still required to explore the role of ncRNAs during embryonic and fetal development in humans.

### Epigenetics and aging

Since the late 1990s and early 21st century, researchers have observed that epigenetic changes accompany aging based on data derived from cellular experiments.^43^ Initially, it was unclear whether these epigenetic alterations were a cause or a consequence of aging. Recent work by Yang et al.^44^ has successfully dissociated epigenetic dysregulation from genetic changes, confirming that the collapse of epigenetic modifications is a potent driver of aging. DNA methylation, a central epigenetic mechanism, regulates both development and aging. Notably, global DNA methylation levels in most regeneratively capable tissues tend to decrease with age.^45^ Beyond global changes, studies increasingly report high frequencies of age-related alterations in DNA methylation accumulated in specific cellular regions. These differentially methylated regions associated with aging lead to either the upregulation or repression of downstream genes. For example, age-related hypermethylation within the promoter regions of tumor suppressor and metabolic genes may partially explain the increased susceptibility of the elderly to tumors and various metabolic disorders.^46^ Conversely, DNMT inhibitor decitabine can reverse hypermethylation in tumor suppressors, enhancing their expression and inducing a senescence-like phenotype in tumor cell lines.^47^ Other mechanisms, such as histone modifications and chromatin remodeling, are also strongly linked to aging. A deficiency in sirtuin 7 (SIRT7) and histone methylation patterns like H3K9me2 and H3K27me3, for example, can activate the cyclic guanosine monophosphate-adenosine monophosphate synthase (cGAS)-stimulator of interferon genes pathway, a well-recognized aging-associated signaling pathway, thus exacerbating the aging process.^48,49^ Moreover, the importance of RNA modifications—particularly m6A and m5C—and ncRNA regulation is increasingly studied in aging research.^50–52^

### Epigenetics and cancer

**Fig. 3:**
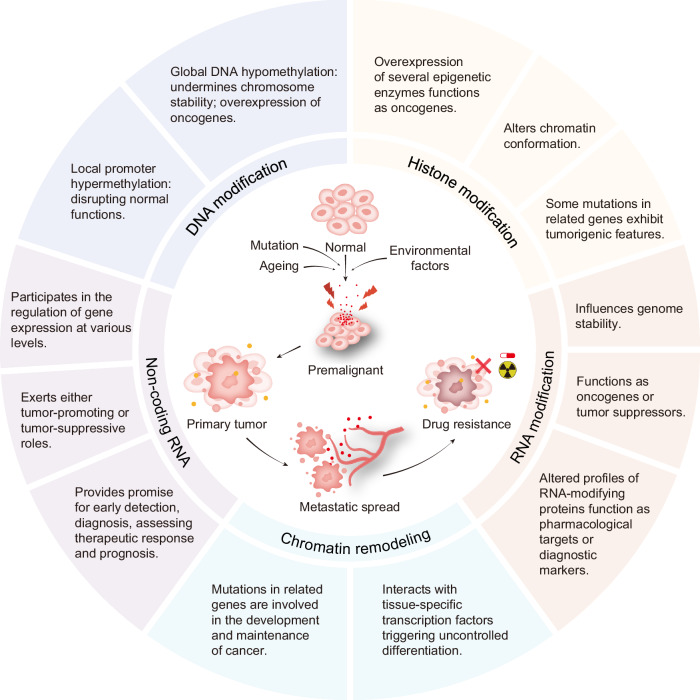
Epigenetic mechanisms in cancer. Epigenetic alterations in cancer cells affect various cellular responses, such as cell proliferation, invasion, apoptosis, and drug resistance. These modifications, which include DNA modification, histone modification, RNA modification, chromatin remodeling, and non-coding RNAs, significantly affect the pathogenesis and progression of tumors. By targeting these epigenetic mechanisms, novel therapeutic strategies for combating cancer can be developed. The primary roles of epigenetic mechanisms in tumorigenesis and their further development are presented in the illustrator

Abnormal epigenetic mechanisms play crucial roles at various stages of tumor development, including initiation, progression, invasion, migration, and the development of chemotherapy resistance (Fig. 3). DNA methylation was the first discovered epigenetic mechanism associated with tumors, initially implicated in the hypermethylation of specific gene promoter regions, which drives tumor development by silencing gene transcription.^53^ This silencing leads to the dysfunction of critical genes such as tumor suppressor and DNA repair genes, disrupting normal cell proliferation and differentiation and fostering the malignant phenotype of tumor cells.^54,55^ Moreover, methylation loss at specific sites in tumor cell genomes, particularly in oncogene promoter regions, and extensive demethylation in DNA repeat sequences, undermines chromosome stability, facilitating tumor development.^56,57^ Changes in histone modifications are also prevalent in tumors. The roles of histone methylation and acetylation in tumor progression have been extensively explored, with numerous reviews summarizing therapeutic strategies targeting these histone modifications or their associated epigenetic-modifying enzymes, underscoring their pathological significance and therapeutic potential.^58,59^ Noticeably, bromodomain (BD) and extra-terminal (BET) family member proteins, including BRD2, BRD3, BRD4, and BRDT, serving as interpreters of histone acetylation modification, have recently been found to facilitate tumorigenesis when overexpressed.^60,61^ Upregulated BET proteins can function as oncogenic transcriptional factors in tissue cells, driving a unique transcriptional program and controlling cell phenotype.^62,63^ Therefore, potent inhibitors targeting BET proteins may be considered potential agents for tumor treatment. Research into metabolic reprogramming and the Warburg effect in tumor cells has recently highlighted histone lactylation’s function in pathological processes.^64,65^ Histone lactylation, induced by glycolysis, has been studied extensively in various malignancies such as endometrial cancer, pancreatic ductal adenocarcinoma, and glioblastoma, where it plays roles in tumor progression and the suppression of the immune microenvironment.^66–68^ Additionally, dysregulation in RNA modifications, particularly m6A, is linked to the malignant potential and resistance of tumor cells,^69,70^ affecting multiple pathways that ensure tumor cell survival, including the maintenance of stemness,^71^ the establishment of vascular networks,^72^ and the formation of an immunosuppressive tumor microenvironment (TME).^73^ Thus, targeting aberrant RNA modifications could effectively disrupt the survival mechanisms of tumor cells, offering new avenues for cancer treatment.^74^ Changes in ncRNA families have also been observed in various tumors, first noted in chronic lymphocytic leukemia with chromosome 13q14 deletion, characterized by decreased levels of miR-15 and miR-16.^75^ Among these, various ncRNA molecules with antitumor effects have been identified and are commonly suppressed in various tumor diseases, representing promising targets for therapeutic intervention.^76^ Furthermore, ncRNAs can participate in the post-translational regulation of other epigenetic-modifying enzymes, integrating into broader epigenetic networks.^77^ In addition to functioning as pathogenic triggers in different tumors, ncRNAs present in extracellular vesicles in the TME also hold promise for assessing therapeutic response.^78–80^ According to clinical data from well-organized observational studies, unique plasma exosomal miRNA profiles are associated with predicting the efficacy of antitumor therapies in various tumor diseases, such as advanced non-small cell lung cancer,^81^ colorectal cancer,^82,83^ and breast cancer.^84^

### Epigenetics and metabolic syndrome and related disorders

Metabolic syndrome encompasses a constellation of pathological conditions characterized by abnormal aggregation of metabolic components, notably abdominal obesity or overweight, dyslipidemia, insulin resistance and/or glucose tolerance abnormalities, and hypertension.^85^ These metabolic dysfunctions significantly elevate the risk of developing diseases such as type 2 diabetes mellitus (T2DM), nonalcoholic fatty liver disease (NAFLD), and cardiovascular diseases.^86^ Epigenetic modifications play a crucial role in nutrient metabolism under physiological conditions and also bridge the genetic and environmental factors contributing to metabolic disorders.^87^ For instance, dietary patterns significantly influence epigenetic markers; studies have shown that a high-fat diet in mice leads to hypermethylation in the promoter regions of genes like Rac family small guanosine triphosphate hydrolase (GTPase) 1, which promotes the progression of diabetic retinopathy.^88^ Dietary-induced epigenetic changes can impact subsequent generations, increasing their risk of glucose intolerance and diabetes.^89^ Moreover, epigenetic alterations linked to diet are implicated in developing gout and NAFLD.^90,91^

Additionally, the activity of epigenetics-modifying enzymes and their cofactors, such as TET and α-ketoglutarate (α-KG) from the tricarboxylic acid cycle (TCA), can be influenced by abnormal metabolite levels in patients with metabolic diseases, further disrupting epigenetic regulation and exacerbating disease progression.^92^ Epigenetic markers, especially DNA methylation landscapes, also provide diagnostic tools;^93^ in T2DM, for example, differential methylation in genes such as thioredoxin interacting protein, adenosine triphosphate (ATP)-binding cassette subfamily G member 1, peroxisome proliferator-activated receptor gamma-coactivator 1 alpha, and protein tyrosine phosphatase receptor type N2, can elucidate pathophysiological mechanisms.^94^ Understanding these epigenetic mechanisms in metabolic diseases is thus pivotal for developing innovative prevention, diagnosis, and treatment strategies.

### Epigenetics and immune system disease

Epigenetic modifications are integral to the development and differentiation of immune cells and the regulation of immune functions. These modifications influence the differentiation of functional B and T cell subpopulations and maintain the homeostasis of innate immune cells by controlling specific gene expressions.^95–97^ Epigenetic dysregulation is closely linked to immune system diseases, including allergic reactions and autoimmune diseases, which have been extensively studied.^98,99^ For instance, allergic bronchial asthma involves reduced TET2 expression in regulatory T cells, leading to hypermethylation in the promoter region of forkhead box protein P3 and impaired immune function in controlling inflammatory responses.^100^ Additionally, low expression of METTL3 in monocyte-derived macrophages in allergy patients exacerbates airway inflammation through M2 macrophage polarization.^97^ Histone modification also plays a critical role in sustaining the therapeutic effects of glucocorticoids in asthma; oxidative stress in severe asthma cases leads to reduced histone deacetylase (HDAC) levels in alveolar macrophages, contributing to glucocorticoid resistance.^101^ Consequently, elevating HDAC levels in patients with severe, steroid-insensitive asthma could be a viable strategy to reduce airway hyperresponsiveness and restore steroid sensitivity.^102^

Epigenetic mechanisms play a significant role in the pathogenesis and progression of autoimmune diseases. For instance, hypomethylation mediated by TET2 within the promoter region of absent in melanoma 2, a critical component of the inflammasome, influences T follicular helper cell-dependent humoral immune responses in systemic lupus erythematosus (SLE).^103^ Additionally, altered patterns of miRNA in serum exosomes and immune cells have been identified, promising potential as biomarkers for diagnosis and indicators of disease severity.^104,105^ Histone modifications also play a pivotal role in SLE, where the administration of HDAC inhibitors has been shown to reduce cytokine profiles and improve pathogenesis in SLE and other inflammatory conditions.^106^ Moreover, the therapeutic potential of BET proteins in antibody-mediated diseases (e.g., SLE) has recently been evaluated. BET inhibitors alter the pro-inflammatory phenotypes of mononuclear phagocytes and impair the recruitment of dendritic cells in vitro.^107^ Beyond SLE, epigenetic mechanisms are implicated in the progression of other autoimmune diseases such as rheumatoid arthritis,^108^ autoimmune thyroid diseases,^109^ multiple sclerosis,^110^ T1DM,^111^ and severe aplastic anemia,^112^ highlighting the potential for epigenetics-modifying drugs in treatment strategies.

### Epigenetics and neurodegenerative disease

Epigenetic modifications significantly influence learning, memory, and cognition, which are essential in maintaining synaptic plasticity.^113,114^ Disruptions in epigenetic regulation lead to the abnormal expression of genes involved in protein aggregation, neuroinflammation, and neuronal apoptosis, contributing to the pathogenesis of neurodegenerative diseases such as Alzheimer’s disease (AD), Parkinson’s disease (PD), and Huntington’s disease (HD).^115^ The deposits of extracellular Aβ plaques and tau phosphorylation, as well as the loss of plasticity, are basic pathogenesis of AD. In AD, aberrant histone modification patterns, particularly histone acetylation, have been observed in hippocampal neurons of AD mouse models, potentially driving cognitive decline and inadequate removal of Aβ plaques.^116^ Lactylation modifications of histones H4K12 and H3K18 affect the metabolic activity of various glial cells, influencing the progression of the AD phenotype.^117,118^ In addition, aberrant DNA-methylation patterns in the promoter regions of functional genes are linked to the accumulation of toxic peptides and the development of memory deficiency.^119,120^ Recent studies also consider RNA modifications and ncRNAs as potential therapeutic targets and diagnostic biomarkers for AD.^121,122^ PD is characterized by the misfolding and aggregation of α-synuclein, leading to the formation of Lewy bodies. Altered DNA methylation patterns have been observed in brain and blood samples from individuals with PD.^123,124^ TET2 may play a critical pathogenic role in PD, where its inactivation has shown a neuroprotective effect on dopaminergic neurons.^125^ Histone acetylation dysregulation is extensively studied in PD, associated with the accumulation of phosphorylated α-synuclein and mitochondrial respiratory dysfunction.^126,127^ Dysregulation in ncRNAs, particularly long non-coding RNAs (lncRNAs) and miRNAs affects the mRNA levels of pathogenic factors post-transcriptionally and is linked with clinical symptoms such as non-motor symptoms, cognitive deficits, and inflammation, presenting potential targets for PD treatment.^128^ In HD, epigenetic modification alterations are vital markers of its pathogenesis. Studies have shown the positive effects of using DNMT inhibitors, HDAC inhibitors, and extracellular vesicles loaded with miRNAs in preventing mutant huntingtin-induced neurotoxicity, emphasizing the potential roles of epigenetic dysregulations in HD.^129–131^ Recently, the impact of aberrant m6A RNA methylation on the progression of HD has been increasingly recognized. Hyper-methylation of m6A in genes related to HD and synaptic function has been linked to memory deficits. Conversely, inhibition of the fat mass and obesity associated protein (FTO) in the hippocampal regions of HD mouse models has shown promise in reversing cognitive symptoms, suggesting a potential therapeutic target.^132^

In summary, the dynamic nature of epigenetic modifications plays a crucial role in maintaining physiological functions and life cycle processes. During embryonic development, precise epigenetic regulation is crucial to cell differentiation and ensures proper tissue specialization by activating or suppressing specific genes. Furthermore, epigenetic modifications are closely linked to an individual’s adaptation to environmental influences such as nutritional status, stress, and toxin exposure, which can alter epigenetic landscapes and impact health and disease risk. On the other hand, understanding epigenetics offers a new perspective for disease prevention and treatment. The development and progression of many diseases, including cancer, metabolic disorders, immune system diseases, and neurodegenerative disorders, are closely associated with aberrant epigenetic modifications. A deeper understanding of epigenetic modulators could lead to novel therapeutic strategies, laying the groundwork for drug interventions targeting epigenetic processes.

## Epigenetics-targeted drugs approved for clinical use

Epigenetic modifications and the enzymes involved can either activate or suppress the expression of specific genes at different levels (Table 1). Therefore, in contrast to traditional therapies, drugs targeting epigenetic-modifying enzymes have been developed with a focus on gene regulation. This unique mechanism provides epigenetic-targeted drugs with an advantage over other traditional treatments, especially for the treatment of tumors. More specifically, epigenetics-targeted drugs specifically target the abnormal epigenetic hallmarks of cancer cells, restoring their normal cellular function or enhancing the immune system’s recognition of tumor cells.^133,134^ Compared to traditional radiotherapy and chemotherapy, which directly kill cancer cells or prevent their proliferation, or immunotherapy, which activates or enhances the patient’s own immune system, the administration of epigenetic agents achieves maximized damage to tumor cells with usually fewer side effects.^135–137^ The application of these novel agents helps to reverse the progression of drug resistance caused by altered epigenetic characteristics in traditional antitumor therapies.^138–140^ Hence, these features endow epigenetic agents with importance and possibilities as monotherapy or adjuvants in combination with other therapeutic methods.^141^ Currently, some of these drugs have been approved for marketing, primarily for cancer treatmentt, and they exhibit exciting clinical potential. These drugs are categorized into four main types based on their mechanisms: DNMT inhibitors, HDAC inhibitors, isocitrate dehydrogenase (IDH) inhibitors, and enhancer of zeste homolog 2 (EZH2) inhibitors (Table 2).

**Table 1:** Key examples of epigenetics-modifying enzymes that are considered targets for drug development and their major biological functions

Epigenetic modification	Type	Epigenetics-modifying enzyme	Biological processes	Reference(s)
DNA methylation	Writer	DNMT1	Maintains DNA methylation after replication	^907^
		DNMT2	Binds to DNA with very weak methyltransferase activity; involved in RNA methylation	^908^
		DNMT3A	Promotes the genome-wide de novo DNA methylation	^909^
		DNMT3B	Promotes the genome-wide de novo DNA methylation	^910,911^
		DNMT3L	Increases the methyltransferase activity of DNMT3A or DNMT3B	^912^
	Eraser	TET1	Active DNA demethylation and binds to DNA via the CXXC zinc finger domain	^913^
		TET2	Active DNA demethylation and binds to DNA via the interaction with DNA binding proteins	^914^
		TET3	Active DNA demethylation and binds to DNA via the CXXC zinc finger domain	^915^
	Reader	MeCP1	Preferentially binds to methylated DNA and represses transcription	^916^
		MeCP2	Binds to a single methyl-CpG pair, not influenced by sequences flanking the methyl-CpGs	^917^
		MBD1	Recruits chromatin-modifying enzymes to both methylated and unmethylated CpG islands; largely silence transcription	^918,919^
		MBD2	A transcriptional repressor or activator depending on the cellular context	^920^
		MBD3	Interacts with NuRD complex to cause transcriptional repression	^921,922^
		MBD4	Exerts DNA glycosylase activity and functions in DNA repair	^923^
		UHRF1	Negatively regulates transcription via the binds to 5hmC and 5mC on DNA, as well as H3K9me3, and H3R2me0; recruits DNMT1 to methylate DNA	^356,358^
		UHRF2	Allosterically activated by 5hmC and participates in DNA demethylation during neuronal commitment	^924–926^
Histone acetylation	Writer	HAT1 (KAT1)	Acetylates H4K12/K5 predominantly; has less activity for H2A	^927–929^
		GCN5 (KAT2A)	Acetylates H3 and H4 and its primary sites are H3K14	^930^
		PCAF (KAT2B)	Acetylates H3 and H4 predominantly and its primary sites are H3K14; has less activity for H2A and HAB	^387^
		CBP/P300 (KAT3A/KAT3B)	Acetylates H2A, H2B, H3, H4 and its primary sites are H3K14/K18/K27	^931,932^
	Eraser	HDAC1	Removes acetylated modifications from H1, H2A, H2B type 1/2 and H3	^933^
		HDAC2	Removes acetylated modifications from H1, H2A, H2B type 1/2 and H3	^933^
		HDAC3	Removes acetylated modifications from H2BK12/K15/K16	^933^
		HDAC4	A very weak deacetylase activity on histone	^934^
		HDAC9	A very weak deacetylase activity on histone	^935^
		SIRT1	Removes acetylated modifications from H1K2, H3K9, and H4K16	^936,937^
		SIRT2	Removes acetylated modifications from histones during G2/M transition and mitosis	^938,939^
		SIRT6	Removes acetylated modifications from H3K9 and H3K56	^940,941^
		SIRT7	Removes acetylated modifications from H3K18	^942^
	Reader	BRD2	Recognizes H4K12ac preferentially	^943^
		BRD4	Recognizes H3K27ac preferentially	^944,945^
		ENL	Recognizes H3K9/K18/27ac preferentially	^946^
		AF9	Recognizes H3K9ac preferentially and H3K27/K18ac to a lesser extent	^946^
		YEATS2	Recognizes H3K9ac preferentially; functions as a selective histone crotonylation reader	^947,948^
		GAS41	Recognizes H3K18/K27ac preferentially; binds to H3K14	^949,950^
Histone methylation	Writer	EZH2	Catalyzes mono-, di-, and tri-methylation of H3K27 and H3K9, as well as non-histones substrates	^951^
		DOT1L	Catalyzes mono-, di-, and tri-methylation of H3K79	^952^
		SETDB1	Catalyzes trimethylation of H3K9	^953^
		GLP/G9a	Catalyzes mono- or di-methylation of H3K9 and non-histone substrates	^954,955^
		SMYD2	Catalyzes both trimethylation of H3K36 and non-histone substrates	^956,957^
		NSD	Catalyzes the dimethylation of H3K36	^958^
		PRMT1	The member of type I PRMTs; the dominant enzyme catalyzing asymmetric dimethylarginine production in proteins and mainly functions as a transcriptional activator	^959^
		PRMT5	The member of type II PRMTs; functions as a transcriptional suppressor or coactivator depending on the cellular context	^960,961^
	Eraser	LSD1(KDM1A)	Removes methylation modifications at H3K4 and H3K9; acts as a coactivator or a corepressor depending on the cellular context	^962^
		KDM2	Removes methylation modifications at H3K4; H3K9, and H3K36; stimulates and inhibits gene transcription	^963^
		KDM7	Removes mono- and di-methylated modifications on H3K9 and H3K27	^964^
		KDM3	Removes mono- and di-methylated modifications from lysine H3K9	^965^
		KDM4	Removes methylated modifications from H3K9 and H3K36	^965,966^
		KDM5	Removes di- and tri-methylated modifications ftom H3K4	^967^
		KDM6A	An X-linked protein removing methylated modifications from H3K27	^968^
		KDM6B	Removes trimethylated modifications from H3K27	^969^
	Reader	MBT	Recognizes lysine residues on H3 and H4, and helps form monomethylated, dimethylated, or trimethylated modifications	^970^
		Chromodomain	Recognizes dimethylated lysine residues of H3K9 and H3K27	^971,972^
		Tudor	Recognizes methylated lysine and arginine residues on histones H3 and H4	^973^
		PWWP	Recognizes H3K36me2/3; binds to dsDNA in a non-specific manner	^974,975^
		PHD	Recognizes H3K4me2/3/0, H3K14ac or H3K27me0 to a lesser extent	^976^
		WDR	Recognizes lysine and arginine methylation of H3	^977^
RNA methylation	writer	METTL3	Catalyzes m6A methylation	^978^
		METTL14	Binds to METTL3 and enhances the catalytic activity of METTL3	^979^
	Eraser	FTO	RNA m6A demethylation; regulates RNA splicing	^980,981^
		ALKBH5	RNA m6A demethylation; regulates RNA metabolism and export	^982^
		ALKBH3	Removes the methyl group at the m6A from tRNA; functions an m1A demethylase	^983^
	Reader	YTHDF1	Responsible for mRNA translation	^984^
		YTHDF2	Responsible for mRNA degradation	^984,985^
		IGF2BP	Regulates mRNA translation	^714^
Chromatin remodeling	Mover	SMARCA2	DNA-stimulated ATPase in the SWI/SNF complex	^986^
		SMARCA4	DNA-stimulated ATPase in the SWI/SNF complex; binds to acetylated peptides on H3 and H4	^986,987^
	Reader	Polybromo-1	Recognizes H3K14ac preferentially	^988^
		BRD7	Recognizes acetylated modifications on histones and non-histones substrates	^989,990^
		BRD9	Recognizes butyryllysine, and crotonyllysine histone peptide modifications	^991^

*AF9* acute lymphocytic leukemia 1 (ALL1)-fused gene from chromosome 9 protein, *ALKBH* ALKB homolog, *ARID* AT-rich interactive domain, *ATPase* adenosine triphosphate hydrolase, *BAF* BRG1-associated factor, *BRD* bromodomain-containing protein, *CBP/P300* cyclic adenosine monophosphate-responsive element-binding protein (CREB)-binding protein/histone acetyltransferase P300, DNMT DNA methyltransferase, *DOT1L* disruptor of telomeric silencing-1-like, *dsDNA* double-stranded DNA, *ENL* eleven–nineteen leukemia, *EZH2* enhancer of zeste homolog 2, *FTO* fat mass and obesity associated protein, *GAS41* glioma amplified sequence 41, *GCN5* general control non-depressible 5, *GLP* G9a-like protein, *HAT* histone acetyltransferase, *HDAC* histone deacetylase, *H4K12ac* histone 3 lysine 12 acetylation, *H3K9me3* histone 3 lysine 9 trimethylation, *IGF2BP* insulin-like growth factor 2 mRNA-binding protein, *KAT* lysine acetyltransferase, *LSD1(KDM1A)* lysine-specific demethylase 1, *m6A* N6-methyladenosine, *MBD* methyl-CpG binding domain protein, *MBT* malignant brain tumor, *MeCP* methy-CpG-bindig protein, *METTL* methyltransferase-like, *NSD* nuclear receptor binding SET domain protein, *NuRD* nucleosome remodeling and deacetylase, *PBAF* polybromo, brahma-related gene 1-associated factor, *PCAF* P300/CBP associated factor, *PHD* plant homeodomain, *PRMT* protein arginine methyltransferase, *PWWP* proline-tryptophan-tryptophan-proline, *SETDB1* SET domain bifurcated histone lysine methyltransferase 1, *SIRT* sirtuin, *SMARCA2* SWI/SNF-related, matrix-associated, actin-dependent regulator of chromatin A2, SMYD2 SET and MYND domain containing 2, *SWI/SNF* Switch/Sucrose nonfermentable chromatin-modifying complex, *TET* ten-eleven translocation, *UHRF1* ubiquitin-like with PHD and RING finger domains 1, *WDR* WD40 repeat, *YEATS2* YAF9, eleven-nineteen-leukemia protein (ENL), acute lymphocytic leukemia 1-fused gene from chromosome 9 protein (AF9), TAF14, and SAS5 (YEATS) domain-containing 2, *YTHDC1* YTH domain-containing protein 1, *YTHDF1* YTH domain family protein 1, *5hmC* 5-hydroxymethylcytosine, *5mC* 5-methylcytosine

**Table 2:** Broad indications and common treatment-related adverse events of marketed epigenetic-targeting drugs

Drug(s)	Target(s)	Route(s) of administration	FDA approval	EMA/NMPA approvals	Broad indications	Common grades 3 or worse treatment-related adverse effects reported in clinical trials	Reference(s)
Azacitidine	DNMT1	Intravenous/subcutaneous	Yes	—	Juvenile myelomonocytic leukemia	Thrombocytopenia, neutropenia, anemia, sepsis, infection, and pneumonia	^992–994^
		Subcutaneous	Not approved yet	EMA	AML; CMML; MDS		
		Intravenous	Not approved yet	NMPA	AML; MDS; Philadelphia chromosome positive CML		
Oral azacitidine	DNMT1	Oral	Yes	—	AML	Febrile neutropenia, thrombocytopenia,leukopenia, pneumonia, respiratory failure, bacteraemia, and sepsis	^149,995^
Decitabine	DNMT1	Intravenous	Yes	NMPA	MDS	Febrile neutropenia, thrombocytopenia, anemia, pneumonia, and infection	^996–998^
		Intravenous	Yes	—	CMML; Refractory anemia (with/without) excess blasts		
		Intravenous	Not approved yet	EMA	AML		
Decitabine/Cedazuridine	DNMT1, CDA	Oral	Yes	—	MDS	Thrombocytopenia, febrile neutropenia, pneumonia, respiratory failure, bacteraemia, and sepsis	^831,997,999^
		Oral	Not approved yet	EMA	AML		
Vorinostat	HDACs	Oral	Yes	—	CTCL	Cellulitis, pulmonary embolism, sepsis, anorexia, increased creatinine phosphokinase, rash, and thrombocytopenia	^1000,1001^
Romidepsin	HDACs	Intravenous	Yes	—	CTCL; PTCL	Lymphopenia, neutropenia, leukopenia, thrombocytopenia, infections, and tumor lysis syndrome	^1002–1004^
Belinostat	HDACs	Intravenous	Yes	—	PTCL	Anemia, thrombocytopenia, dyspnea, neutropenia, infections, tumor lysis syndrome, and ventricular fibrillation	^180,1005,1006^
Panobinostat	HDACs	Oral	Canceled by the FDA in 2022	EMA	MDS	QTc prolongation, hemorrhage, thrombocytopenia, lymphopenia, and asthenia	^1007,1008^
Chidamide	Class I HDAC	Oral	Not approved yet	NMPA	Breast cancer; DLBCL; PTCL	Neutropenia, thrombocytopenia, anemia, leukopenia, diarrhea, and mucositis	^202,1009,1010^
Givinostat	HDAC1, HDAC3	Oral	Yes	—	DMD	Diarrhea	^207^
Enasidenib	IDH2	Oral	Yes	—	AML	Febrile neutropenia, IDH differentiation syndrome, and indirect hyperbilirubinemia	^228,234^
Ivosidenib	IDH1	Oral	Yes	NMPA	AML	QT interval prolongation, IDH differentiation syndrome, anemia, and ascites	^1011,1012^
		Oral	Yes	—	Cholangiocarcinoma; MDS		
		Oral	Not approved yet	EMA	IDH1-mutated AML; IDH1-mutated cholangiocarcinoma		
Ivosidenib/Azacitidine	IDH1/DNMT1	Oral; intravenous/subcutaneous	Yes	—	IDH1-mutated AML	Febrile neutropenia, neutropenia, bleeding events, infection, IDH differentiation syndrome, and QT interval prolongation	^240,241^
Olutasidenib	IDH1	Oral	Yes	—	IDH1-mutated AML	Thrombocytopenia, febrile neutropenia, anemia, alanine aminotransferase increased, and aspartate aminotransferase increased	^248,249^
Tazemetostat	EZH2	Oral	Yes	—	FL; Sarcoma	Hyperglycemia, hyponatremia, anemia, thrombocytopenia, neutropenia, lymphopenia, and weight loss	^252,254^
Valemetostat tosilate	EZH2/EZH1	Oral	Not approved yet	^*^Only approved by the PMDA in Japan	ATL	Thrombocytopenia, anemia, lymphopenia, leukopenia, and neutropenia	^261^

*AML* acute myeloid leukemia, *ATL* adult T-cell leukemia/lymphoma, *CDA* cytidine deaminase, CML chronic myelogenous leukemia, *CMML* chronic myelomonocytic leukemia, *CTCL* cutaneous T-cell lymphoma, *DLBCL* diffuse large B-cell lymphoma, *DMD* Duchenne muscular dystrophy, *DNMT1* DNA methyltransferase 1, *EMA* European Medicines Agency, *EZH2* enhancer of zeste homolog 2, *FDA* Food and Drug Administration, *FL* follicular lymphoma, *HDAC* histone deacetylase, *IDH* isocitrate dehydrogenase, *MDS* myelodysplastic syndrome, *NMPA* National Medical Products Administration, *PMDA* Pharmaceuticals and Medical Devices Agency, *PTCL* peripheral T-cell lymphoma

### DNMT inhibitors

Azacitidine and decitabine, known as hypomethylating agents (HMAs), target DNMT1 and were among the first epigenetics-targeted drugs approved for clinical use. The US FDA approved azacitidine in May 2004 and decitabine in June 2006 for treating myelodysplastic syndrome (MDS).^142,143^ The clinical success of these HMAs has led to a focus on optimizing their dosing schedules and administration methods. Early studies involving azacitidine and decitabine assessed their therapeutic potential through continuous and/or frequent intravenous or subcutaneous injections, establishing standard doses and delivery methods in clinical settings.^144–146^ Recent advancements have explored reduced dosages for patients at lower risk. In recent phase II clinical trials, azacitidine or decitabine was applied for three or five consecutive days in a 28-day cycle, exhibiting satisfactory therapeutic efficacy and tolerable safety.^147,148^ Some patients who received decitabine experienced myelosuppression, and future efforts are required to take steps to avoid this.^147^

Additionally, the development of oral formulations of azacitidine and decitabine, such as oral azacitidine (CC-486), approved in September 2020 for patients with acute myeloid leukemia (AML) who are not candidates for intensive curative therapy, has improved patient convenience and treatment adherence.^149^ In a well-organized phase III randomized trial, the median overall survival and relapse-free survival of patients treated with CC-486 were greatly improved.^149^ Importantly, fewer grades 3 or 4 adverse events were observed during CC-486 treatment, allowing the preservation of overall health-related quality of life.^149^ The doses of CC-486 used in clinical settings are approximately four times higher than the standard doses administered by intravenous or subcutaneous routes due to their reduced bioavailability.^150^ Meanwhile, the poor bioavailability of oral decitabine has led to the development of ASTX727, an oral combination of decitabine with cedazuridine. This cytidine deaminase enzyme inhibitor enhances decitabine exposure after oral administration. This combination has been approved for marketing in treating MDS and AML in some countries.^151,152^ However, in China, the therapeutic potential of CC-486 and ASTX727 for AML and MDS is still under evaluation in clinical trials (NCT05413018, NCT04102020, NCT06091267, NCT02649790). Furthermore, there has been an increased focus on the synergistic effects of HMA and traditional antitumor treatments to enhance therapeutic outcomes and prevent resistance in hematologic malignancies refractory to monotherapy. This topic is further summarized in the subsequent section on drug combination applications.

Inspired by the successful application of HMAs in hematologic malignancies, their therapeutic potential in treating solid tumors is also being explored. However, as of now, HMAs are not approved for treating solid tumors. In 2017, Linnekamp et al.^153^ conducted a systematic review to illustrate the clinical and biological effects of HMAs on solid tumors based on previously completed clinical trials. The efficacy of azacitidine and decitabine in solid tumors was less pronounced than in hematological malignancies, primarily because most study participants had advanced-stage tumors with short life expectancies.^153^ Moreover, many early-stage studies were small-sized cohorts, lacking sufficient evidence to generalize therapeutic effects across different tumor types. With significant advances in optimizing HMA formulations and dosages, as well as the increasing number of combination therapies showing promising effects on solid tumors in vitro and in vivo, clinical trials of HMAs, particularly the oral formulations CC-486 and ASTX727, among patients with solid tumors, are being extensively conducted, and their results are eagerly anticipated.^154–156^

### HDAC inhibitors

Over the past two decades, substantial progress has been made in developing HDAC inhibitors, with six approved for clinical use. These include vorinostat (SAHA), romidepsin (FK228), belinostat (PXD101), and panobinostat (LBH589, although the FDA canceled it in 2022). These drugs have been approved by various regulatory bodies, such as the US FDA, the European Medicines Agency, and the Pharmaceuticals and Medical Devices Agency (PMDA). They are used to treat conditions such as multiple myeloma (MM), cutaneous T-cell lymphoma (CTCL), and peripheral T-cell lymphoma (PTCL).^157,158^ Additionally, chidamide (tucidinostat) was approved by PMDA in Japan and National Medical Products Administration (NMPA) in China for the treatment of PTCL and advanced breast cancer,^159,160^ and more recently, givinostat (ITF2357) was approved by the FDA in March 2024 as the first nonsteroidal treatment for Duchenne muscular dystrophy (DMD) for patients aged six years and older.^161^

Vorinostat was the first pan-inhibitor of HDACs approved by the FDA in October 2006 for CTCL.^162^ Soon after, in July 2011, it was also approved for clinical therapy by PMDA. In addition to CTCL, the application of vorinostat to AML, MM, malignant pleural mesothelioma, newly diagnosed high-grade glioma (NCT01236560), and advanced non-small cell lung cancer (NCT00473889) therapy has entered phase III clinical trials.^163–166^ Disappointingly, though vorinostat exerts effective therapeutic effects in diverse hematological malignancies, limited efficacy has been observed in solid tumors.^166^ Another thing to note when using vorinostat as a clinical medication is the potential adverse events that may occur. While generally mild, adverse events related to vorinostat can include thrombosis, QT interval prolongation, and potentially fatal ventricular tachycardia or torsional tachycardia.^167–169^ These findings have driven the development of other HDAC inhibitors, with the expectation of elevated safety and efficacy in vivo.

Romidepsin, another pan-HDAC inhibitor, was approved by the FDA in November 2009 for CTCL and later for PTCL. It has shown a higher affinity to class I HDAC proteins.^170^ Subsequently, it was approved for treating patients with PTCL by the FDA and PMDA. Phase III randomized controlled trials are performed to evaluate the therapeutic value of the first-line treatment of PTCL, referring to the combination of cyclophosphamide, doxorubicin, vincristine, prednisone (CHOP), and romidepsin plus CHOP in patients with PTCL, while the addition of romidepsin failed to increase efficacy as expected.^171,172^ However, after a six-year follow-up, the application of romidepsin shows beneficial effects in prolonging median progression-free survival.^171^ In addition, the combination of romidepsin with other therapeutics, such as oral 5-azacytidine, tenalisib (an inhibitor of phosphoinositide-3-kinase and salt-inducible-kinase-3), and lenalidomide (a new generation of immune modulator) shows promising therapeutic potential in various types of T-cell lymphoma in the initial stages of clinical practice, supporting further exploration.^173–176^ Meanwhile, investigations on the therapeutic effects of romidepsin against other diseases are ongoing, particularly in antiretroviral treatment in human immunodeficiency virus-1 (HIV-1) infection.^177,178^

Belinostat was FDA-approved in July 2014 for relapsed or refractory (R/R) PTCL, showing pan-inhibitory effects on HDAC proteins.^179^ Common adverse effects of belinostat include nausea, vomiting, diarrhea, dysgeusia, fatigue, and severe hematologic treatment-related adverse events.^180,181^ Further, dosing considerations are needed for patients with hepatic impairment due to liver metabolism.^182^ Belinostat is being explored for other myeloid malignancies and solid tumors, including glioblastoma and small-cell lung cancer.^183–186^

Panobinostat, an oral broad-spectrum HDAC inhibitor approved in January 2015 for R/R MM in combination with dexamethasone and bortezomib, showed a slight overall survival benefit in phase II and III trials.^187^ However, many participants experienced adverse events like thrombocytopenia, lymphopenia, asthenia, and fatigue, which raises concerns about its tolerability.^187–189^ In a recent randomized phase II clinical trial, it was proposed that administering bortezomib via subcutaneous application rather than intravenous injection could improve the safety and tolerability of the triplet regimen, including panobinostat.^190^ Beyond its primary indications, panobinostat is being explored for its efficacy in various other tumor diseases such as lymphoma, primary myelofibrosis, glioma, clear cell renal cell carcinoma, and prostate cancer, both as monotherapy and in combination with other tumor therapeutics, showing promising efficacy across multiple malignancies.^191–195^ However, the safety of panobinostat continues to be a primary concern and requires further evaluation in advanced clinical studies.

Chidamide, the first orally administered selective HDAC inhibitor targeting HDAC1, HDAC2, HDAC3, and HDAC10,^160^ is currently under investigation for a variety solid and hematological malignancies, autoimmune diseases, and neurodegenerative diseases.^196–201^ It offers advantages over pan-inhibitors in the treatment of tumor diseases and in minimizing severe adverse effects.^160^ Recent therapeutic strategies using chidamide in combination with a second antitumor intervention have shown promising prospects. For instance, a combination of chidamide and exemestane has proven effective as a neoadjuvant treatment for patients with stage II-III breast cancer that is hormone receptor-positive and human epidermal growth factor receptor 2-negative.^199,202^ A recent phase III clinical trial reported an increased occurrence of grades 3–4 hematological adverse events in the tucidinostat plus exemestane group, while the median progression-free survival of these patients was notably improved.^202^ Furthermore, synergistic effects have been observed when chidamide is used in conjunction with immunotherapy, endocrine therapy, or chemoradiation, offering novel adjuvant approaches for tumor therapy.^203–206^

Givinostat, developed by Italfarmaco SpA, is a potent inhibitor of HDAC1 and HDAC3 recently approved for clinical use. In a pivotal, multicenter, randomized phase III clinical trial involving 179 patients with DMD aged at least six years, givinostat effectively delayed disease progression.^207^ The most common adverse events reported were diarrhea and vomiting.^207^ Additionally, givinostat shows promise as a treatment for polycythemia vera, particularly in patients unresponsive to hydroxycarbamide monotherapy.^208^ In phase I/II clinical trials, givinostat demonstrated promising efficacy and tolerability in patients with polycythemia vera.^209,210^ Subsequently, long-term follow-up over four years has further substantiated the therapeutic benefits and safety profile of givinostat.^211^ Throughout the follow-up period, the overall response rate consistently exceeded 80% among patients with PV, while only 10% of these patients experienced grade 3 treatment-related adverse events, suggesting its potential for prolonged clinical use.^211^

### IDH inhibitors

IDH is a key enzyme in the TCA cycle that normally catalyzes the conversion of isocitrate to α-KG and carbon dioxide. In cells with mutated IDH, this enzyme instead produces 2-hydroxyglutarate (2-HG), a metabolite that inhibits α-KG-dependent epigenetic enzymes and contributes to the aberrant epigenetic landscape seen in various diseases, particularly tumors.^212,213^ Currently, three IDH inhibitors are approved for clinical use: enasidenib (AG-221), ivosidenib (AG-120), and olutasidenib (FT-2102), targeting different forms of the enzyme mutation.^214–217^ Additional IDH1/2 inhibitors that are allowed to be investigated in clinical practice include the dual inhibitor of mutant IDH1/2 vorasidenib (AG-881),^218^ the irreversible mutant IDH1 inhibitor LY3410738 (NCT06181045, NCT06181084), and the pan-mutant-IDH1 inhibitor BAY1436032.^219,220^

Enasidenib is an allosteric inhibitor targeting mutated IDH2, approved by the FDA in August 2017 for the treatment of R/R AML with IDH2 mutations.^221^ Based on preliminary animal experiments and preclinical evidence, enasidinib effectively reduces the 2-HG levels derived from IDH2 mutations, reversing excessive histone and DNA methylation landscapes.^222–224^ Subsequently, enasidinib has entered clinical trials and demonstrated good efficacy in treating AML and MDS patients, which is further considered a promising option for elderly AML patients over 60 years old, especially those who are not suitable for intensified chemotherapy.^225–230^ Combination therapy with enasidenib and azacitidine has shown acceptable tolerability and potential to improve outcomes for patients with IDH-mutated AML.^229,231,232^ However, potential severe adverse effects include hyperbilirubinemia, thrombocytopenia, pneumonia, and IDH differentiation syndrome, the latter of which can be life-threatening and requires careful management.^233–235^ Noticeably, IDH differentiation syndrome is one of the potentially lethal entities that require prompt recognition and more appropriate management.^236^ Enasidenib is also being explored for other conditions caused by IDH2 mutations, such as D-2-hydroxyglutaric aciduria type II,^237^ chondrosarcoma,^238^ angioimmunoblastic T-cell lymphoma (NCT02273739), and malignant sinonasal or skull base tumors (NCT06176989).

Ivosidenib, targeting mutated IDH1, was first approved by the FDA in July 2018 for R/R AML.^239^ In April 2022, with the data from a completed phase III clinical trial being made public, the therapeutic potentials and good safety of the combination of azacitidine and ivosidinib among patients with AML received broader attention.^240^ Subsequently, the FDA approved this regimen for elderly patients with newly diagnosed IDH1-mutated AML in May 2022.^241^ Besides AML, ivosidenib is approved for MDS and cholangiocarcinoma, with ongoing phase III studies in unresectable or metastatic cholangiocarcinoma with IDH1 mutations.^216,242^ The most significant adverse events include ascites and other severe conditions, necessitating vigilant monitoring.^242^ Ivosidenib has also shown promising results in phase I clinical trials for IDH-mutated advanced glioma, with a daily dose of 500 mg proving effective in reducing 2-HG levels and controlling the disease.^243–246^

Olutasidenib, an oral IDH1 inhibitor, was approved by the FDA in December 2022 for treating R/R AML with specific IDH1 mutations. It has also shown promise as a therapeutic option for patients with IDH1-mutated AML who are insensitive to venetoclax, offering a new avenue for treatment where previous therapies may have failed.^217^ Clinical trials have demonstrated that olutasidenib, combined with azacitidine, provides comparable efficacy and tolerability in AML and MDS patients harboring mutant IDH1.^247^ Treatment-emergent side effects of grade 3-4, such as febrile neutropenia, anemia, thrombocytopenia, and neutropenia, occur at a low frequency with olutasidenib monotherapy or in combination therapies, suggesting a manageable safety profile.^247,248^ Beyond hematological malignancies, the therapeutic potential of olutasidenib is also being explored in other diseases, such as IDH-mutated R/R gliomas. In a multicenter, open-label, phase Ib/II clinical trial involving 26 patients, olutasidenib achieved a disease control rate of 48%. Notable grade 3-4 adverse events included increases in alanine aminotransferase and aspartate aminotransferase, indicating the need for careful liver function monitoring during treatment.^249^

### EZH2 inhibitors

Currently, two EZH2 inhibitors, tazemetostat (EPZ-6438) and valemetostat tosilate (DS-3201, DS-3201B), targeting EZH1/2 or EZH2 have been approved and are being utilized in various therapeutic strategies.

Tazemetostat, the first oral EZH2 inhibitor, was approved by the FDA in January 2020 for patients over 16 years of age with advanced epithelioid sarcoma that is ineligible for complete resection.^250^ It is also the first targeted drug for epithelioid sarcoma treatment.^251^ In a phase II clinical trial (NCT02601950), tazemetostat demonstrated good tolerability and clinical activity, with a low incidence of severe treatment-related adverse events such as anemia and weight loss.^252^ Tazemetostat has also been studied as a monotherapy in R/R follicular lymphoma (FL), showing promising, durable responses and an acceptable safety profile.^253,254^ Common severe adverse events included thrombocytopenia, neutropenia, and anemia.^254^ In Japan, a phase I/II trial evaluated tazemetostat 800 mg twice daily in R/R EZH2 mutation-positive FL, showing encouraging response rates and tolerability, which helped to accelerate its approval by the FDA and PMDA for this indication.^255,256^ Furthermore, tazemetostat is being investigated as a single agent for malignant mesothelioma, with ongoing efforts to refine biomarkers for its activity in malignant pleural mesothelioma.^257^ Research is also underway to assess the efficacy and tolerability of tazemetostat in combination with other therapeutic agents, including programmed cell death 1 (PD-1)/programmed cell death 1 ligand 1 (PD-L1) inhibitors, chemotherapy, and targeted therapeutics across different tumor types.^258–260^ Notably, phase III clinical trials are exploring tazemetostat in combination with lenalidomide and rituximab (NCT04224493) or doxorubicin (NCT04204941) focusing on R/R FL and epithelioid sarcoma, which are highly anticipated for their potential to redefine treatment paradigms.

Valemetostat tosilate is an innovative dual inhibitor of EZH1/2 that received approval from the PMDA in Japan for the treatment of R/R adult T-cell leukemia/lymphoma (ATL) in September 2022.^261^ It is administered orally and should be taken on an empty stomach to avoid adverse food effects.^262^ Dosage adjustments are necessary when valemetostat is used concurrently with strong inhibitors of cytochrome P450 3 A and P-glycoprotein, which can affect its metabolism and excretion.^263^ In a multicenter phase 2 trial involving patients with R/R aggressive ATL, valemetostat demonstrated promising efficacy, even in heavily pretreated patients. The common treatment-associated adverse effects reported were manageable, including thrombocytopenia, anemia, alopecia, dysgeusia, neutropenia, lymphopenia, leukopenia, decreased appetite, and pyrexia.^264^ However, resistance to valemetostat has been observed in some patients with ATL, potentially due to acquired mutations in the polycomb repressive complex 2 (PRC2) within tumor cells, highlighting a significant challenge in long-term treatment scenarios.^59^ Currently, valemetostat and its analogs are also being investigated in various preclinical studies and animal models for conditions such as tumor protein p63 gene-rearranged lymphoma, sinonasal teratocarcinosarcoma, ibrutinib-resistant mantle cell lymphoma, and human T-cell leukemia virus type 1-associated myelopathy. These studies further expand the potential therapeutic applications of valemetostat and warrant continued exploration of valemetostat-based treatment strategies.^265–268^

## Other epigenetics-targeted drugs under research as clinical candidates

**Fig. 4:**
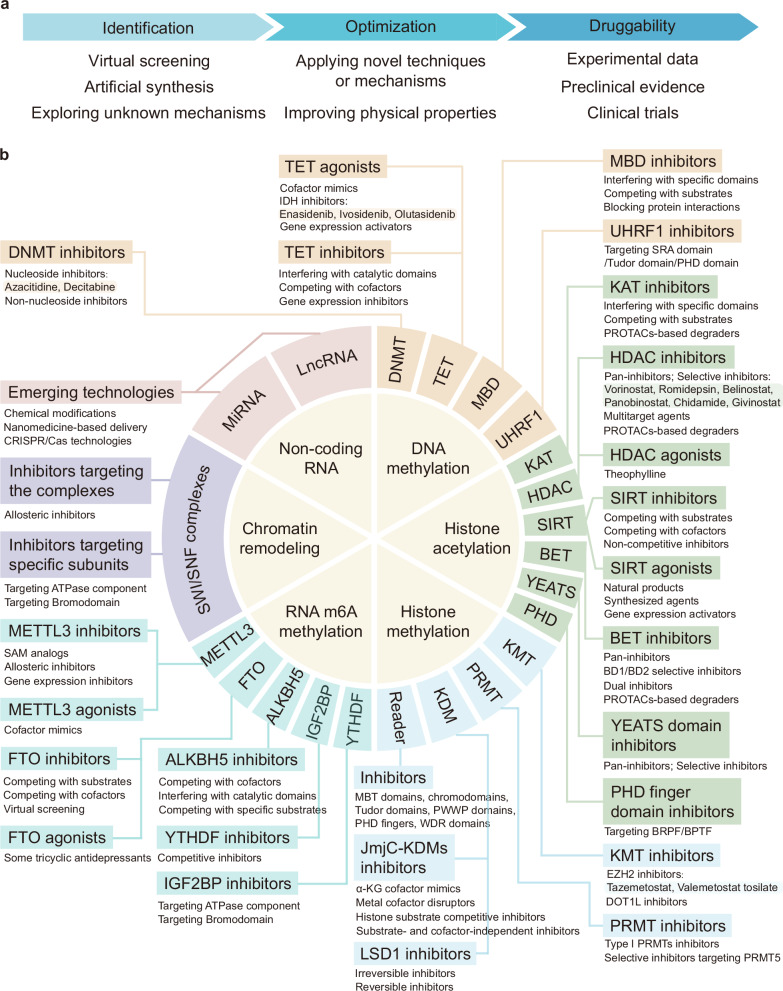
The development direction and major categories of epigenetics-targeted drugs. **a** Epigenetics-targeted drugs are developed through the virtual screening of compound libraries, drug design based on molecular structure, and the exploration of potential mechanisms of known agents. Subsequently, applying PROTAC, CRISPR/Cas, and other technologies or mechanisms to optimize the physical properties, and inhibitory or agonistic effects of compounds. Finally, the druggability of possible agents should be improved in experimental, preclinical and clinical studies. **b** The classification of epigenetics-targeted drugs and their corresponding marketed representative agents are depicted in this section. Among them, epigenetics-targeted drugs that have already been approved and applied in clinical treatment are highlighted in corresponding colors

Although there have been many advances in the research of marketed drugs, they still belong to the tip of the iceberg relative to the entire field of epigenetics-targeting drug development. Many small molecule inhibitors and agonists targeting epigenetic-modifying enzymes are being identified and progressively advancing into the early stages of clinical trials. This section categorizes and summarizes these drugs based on their mechanisms, highlighting agents that show potential therapeutic value in clinical settings (Fig. 4).

### Epigenetics-targeted drugs and DNA methylation

This critical regulatory mechanism of gene expression involves adding methyl groups to DNA, primarily at cytosine bases in CpG dinucleotides, which generally leads to gene silencing. The process is dynamically regulated by DNMT and TET enzymes. The aberrant activity of these enzymes is closely linked with the pathogenesis of a wide range of diseases, not only cancers but also metabolic, inflammatory, and neurological disorders, underscoring the therapeutic relevance of targeting these pathways.^269–272^ Therefore, DNA methylation provides a promising platform for developing epigenetics-targeted drugs in clinical practice (Table 3).

**Table 3:** Summary of DNA methylation-targeted drugs for different diseases in clinical trials

Type	Drug	Target(s)	Condition(s)	Status/outcome(s)	Phase(s)	Other intervention(s)/drug(s)	Study ID/reference(s)
DNMT inhibitor	Guadecitabine	DNMT1	Platinum refractory germ cell cancer	Completed (exhibits tolerable safety and satisfied activity)	Phase I	In combination with Cisplatin	NCT02429466^1013^
	Guadecitabine	DNMT1	Liver cancer, pancreatic cancer, bile duct cancer, gallbladder cancer	Active, not recruiting	Phase I	In combination with Durvalumab	NCT03257761
	Guadecitabine	DNMT1	Lung cancer	Active, not recruiting	Phase I	In combination with Pembrolizumab and Mocetinostat	NCT03220477
	Guadecitabine	DNMT1	AML	Completed (exhibits an overall unfavorable benefit-risk profile at the investigated dose levels)	Phase I	In combination with Atezolizumab	NCT02892318^276^
	Guadecitabine	DNMT1	Colorectal cancer	Completed (no significant clinical activity of the Guadecitabine with Cy/GVAX is observed)	Phase I	CY/GVAX (active comparator/followed by Guadecitabine)	NCT01966289^277^
	Guadecitabine	DNMT1	Castration-resistant prostatic cancer, NSCLC	Recruiting (helps to reverse resistance to immune checkpoint inhibitors according to early clinical data)	Phase I	ASTX727 (active comparator); in combination with Pembrolizumab	NCT02998567^1014^
	Guadecitabine	DNMT1	AML	Completed (subcutaneous administration of large doses may be beneficial for improving treatment efficacy while increases the risk of adverse events)	Phase I	—	NCT02293993
	Guadecitabine	DNMT1	Melanoma	Completed (helps to achieve long-term clinical benefits)	Phase I	In combination with Ipilimumab	NCT02608437^281,282^
	Guadecitabine	DNMT1	SCLC	Completed (unpublished)	Phase I	In combination with platinum-based first-line chemotherapy, Durvalumab, and Tremelimumab	NCT03085849
	Guadecitabine	DNMT1	AML, MDS	Terminated (not due to patient safety)	Phase II	—	NCT03603964
	Guadecitabine	DNMT1	MDS	Active, not recruiting	Phase II	—	NCT02131597
	Guadecitabine	DNMT1	AML, MDS	Unknown	Phase II	ASCT	NCT03454984
	Guadecitabine	DNMT1	Paraganglioma, GIST, RCC, pheochromocytoma	Terminated (exhibits manageable toxicity with low objective response rates)	Phase II	—	NCT03165721^278^
	Guadecitabine	DNMT1	SCLC	Completed (exhibits good efficacy but with the possibility of adverse events)	Phase II	In combination with Cisplatin	NCT03913455^1015^
	Guadecitabine	DNMT1	Urothelial carcinoma	Active, not recruiting (possibly prolongs patient survival)	Phase II	In combination with Atezolizumab	NCT03179943^1016^
	Guadecitabine	DNMT1	Philadelphia-negative myeloproliferative neoplasms	Completed (helps improve quality of life and exhibits acceptable adverse events)	Phase II	—	NCT03075826
	Guadecitabine	DNMT1	HCC	Completed (high incidence of treatment-related adverse events)	Phase II	—	NCT01752933
	Guadecitabine	DNMT1	AML	Completed (exhibits comparable clinical response rates and safety)	Phase II	With or without Cladribine or Idarubicin	NCT02096055
	Guadecitabine	DNMT1	Fallopian tube carcinoma, peritoneal carcinoma	Completed (exhibits clinical benefit and possibly activates antitumor immunity)	Phase II	In combination with Pembrolizumab	NCT02901899^1017^
	Guadecitabine	DNMT1	MDS	Completed (exhibits potential therapeutic effects on high-risk patients who failed azacitidine)	Phase II	—	NCT02197676^1018^
	Guadecitabine	DNMT1	AML, CMML, MDS	Active, not recruiting	Phase II	In combination with donor lymphocytes	NCT02684162
	Guadecitabine	DNMT1	Melanoma, NSCLC	Not yet recruiting	Phase II	With or without Ipilimumab plus Nivolumab	NCT04250246
	Guadecitabine	DNMT1	Central chondrosarcoma	Active, not recruiting	Phase II	In combination with Belinostat or ASTX727	NCT04340843^1019^
	Guadecitabine	DNMT1	Ovarian cancer	Completed (helps to increase progression-free survival within six months)	Phase II	In combination with Carboplatin	NCT01696032^1020^
	Guadecitabine	DNMT1	Colorectal cancer	Withdrawn (due to the insufficient funding)	Phase I/II	In combination with Nivolumab	NCT03576963
	Guadecitabine	DNMT1	RCC	Active, not recruiting (exhibits satisfied safety and tolerability)	Phase I/II	In combination with Durvalumab	NCT03308396^1021^
	Guadecitabine	DNMT1	AML, MDS, CMML	Active, not recruiting (exhibits manageable adverse events and typical cytopenia-related safety concerns)	Phase I/II	In combination with Atezolizumab	NCT02935361^1022^
	Guadecitabine	DNMT1	AML, MDS, CMML	Completed (exhibits well clinically active and acceptable tolerability)	Phase I/II	—	NCT01261312^1023–1026^
	Guadecitabine	DNMT1	Colorectal cancer	Completed (exhibits comparable efficacy and safety profiles)	Phase I/II	In combination with Irinotecan; Regorafenib or TAS-102 (active comparator)	NCT01896856^280,1027^
	Guadecitabine	DNMT1	Platinum-resistant fallopian tube carcinoma, platinum-resistant ovarian carcinoma, platinum-resistant primary peritoneal carcinoma	Active, not recruiting	Phase I/II	Atezolizumab (active comparator/followed by Guadecitabine); with or without CDX-1401 vaccine	NCT03206047
	Guadecitabine	DNMT1	MDS, CMML	Completed (exhibits comparable therapeutic effects and safety profiles)	Phase III	Low-dose Cytarabine/standard IC/BSC (active comparator)	NCT02907359
	Guadecitabine	DNMT1	AML	Completed (exhibits higher clinical response rates and comparable safety)	Phase III	High-dose Cytarabine/low-dose Cytarabine/BSC(active comparator)	NCT02920008^279^
	Guadecitabine	DNMT1	AML	Completed (no significant clinical activity of the Guadecitabine and active comparators is observed)	Phase III	Low-dose Cytarabine/high-dose Cytarabine (active comparator)	NCT02348489^283^
	MG98	DNMT1	Solid tumors	Completed (exhibits early evidence of clinical activity with good tolerability)	Phase I	—	NCT00003890^297^
	MG98	DNMT1	Metastatic renal carcinoma	Terminated (exhibits no antitumor activities)	Phase II	—	^298^
	Hydralazine	DNMT1/3a/3b	Lung cancer	Completed (unpublished)	Phase I	In combination with Valproic acid	NCT00996060
	Hydralazine	DNMT1/3a/3b	Refractory solid tumors	Completed (exhibits the potential to overcome chemotherapy resistance)	Phase II	In combination with Magnesium valproate	NCT00404508^304^
	Hydralazine	DNMT1/3a/3b	Cervical cancer	Completed (unpublished)	Phase II	In combination with Magnesium valproate and Cisplatin chemoradiation	NCT00404326
	Hydralazine	DNMT1/3a/3b	BC	Terminated (treatment is well-tolerated)	Phase II	In combination with Magnesium valproate	NCT00395655^301^
	Hydralazine	DNMT1/3a/3b	HCC	Completed (exhibits good efficacy and manageable toxicities)	Phase II	In combination with Valproic acid	TPVGH97-07-07^1028^
	Hydralazine	DNMT1/3a/3b	MDS	Unknown	Phase II	In combination with Valproic acid; BSC (active comparator)	NCT01356875
	Hydralazine	DNMT1/3a/3b	BC	Withdrawn (IRB request)	Phase I/II	—	NCT00575978
	Hydralazine	DNMT1/3a/3b	Rectal cancer	Withdrawn (No enrollment)	Phase I/II	—	NCT00575640
	Hydralazine	DNMT1/3a/3b	Ovarian cancer	Unknown	Phase III	In combination with Magnesium valproate; placebo-controlled	NCT00533299
	Hydralazine	DNMT1/3a/3b	Cervical cancer	Completed (exhibits advantages in progression-free survival)	Phase III	In combination with Magnesium valproate; placebo-controlled	NCT00532818
	Hydralazine	DNMT1/3a/3b	Cervical cancer	Unknown	Phase III	In combination with Magnesium valproate, Carboplatin, and Paclitaxel; placebo-controlled	NCT02446652
TET agonist	Vitamin C/Ascorbate	TET1//2/3	MDS, AML	Completed (enhances the biological effects of DNMT inhibitors)	Pilot trial	In combination with Azacitidine	NCT02877277^335^
	Vitamin C/Ascorbate	TET1//2/3	MDS, AML	Completed (identifies an appropriate dose of the drug combination for phase II studies)	Phase I	In combination with Decitabine and Arsenic trioxide	NCT00671697^336^
	Vitamin C/Ascorbate	TET1//2/3	TET2-mutant MDS, TET2-mutant AML	Completed (unpublished)	Phase II	In combination with Azacitidine	NCT03397173
	Vitamin C/Ascorbate	TET1//2/3	TET2-mutant MDS	Completed (exhibits good safety profiles and tolerability)	Phase I/II	—	NCT03433781

*AML* acute myeloid leukemia, *ASCT* allogeneic stem cell transplant, *BC* breast cancer, *BSC* best supportive care, *CMML* chronic myelomonocytic leukemia, *CY* Cyclophosphamide/Cytoxan, *DNMT* DNA methyltransferase, *GVAX* colon cancer tumor vaccine, *GIST* gastrointestinal stromal tumor, *HCC* hepatocellular carcinoma, *IC* intensive chemotherapy, *IRB* institutional review board, *MDS* myelodysplastic syndrome, *NSCLC* non-small cell lung cancer, *RCC* renal cell carcinoma, *SCLC* small cell lung cancer, *TET* ten-eleven translocation

#### Targeting the writer of DNA methylation: DNMT

Current therapeutic strategies primarily involve DNMT inhibitors, which suppress the expression or enzymatic activities of DNMTs, thereby counteracting improper DNA methylation patterns. These inhibitors are crucial in correcting the abnormal addition of methyl groups to DNA, a common feature in many pathologies.

##### DNMT inhibitors

Most DNMT inhibitors under investigation are employed in treating hematological or solid tumors, with a smaller portion used for inflammatory or proliferative benign diseases.^153,273^ DNMT inhibitors fall into two principal categories based on their mechanisms of action: nucleoside DNA methylation inhibitors and non-nucleoside DNA methylation inhibitors.

Among the nucleoside analogs, the FDA-approved drugs azacitidine and decitabine are noteworthy. These compounds integrate into the DNA structure and are recognized by DNMTs during DNA replication, thereby obstructing normal DNA methylation processes.^274^ Another significant compound, guadecitabine (SGI110, an antimetabolite of decitabine), represents a second-generation DNA methylation inhibitor. It is an antinucleotide molecule that resists degradation by cytidine deaminase.^275^ Research primarily focuses on its application against various malignant tumors. However, although guadecitabine has demonstrated good tolerance and favorable outcomes in many clinical trials, there is still clinical evidence indicating that its application may cause serious treatment-related adverse effects, such as pneumonia, sepsis, aspiration pneumonia, metabolic disorders, neutropenia, leukopenia, and pruritis.^276,277^ Furthermore, a recent phase II clinical trial conducted among patients with succinate dehydrogenase-deficient tumors was terminated due to low objective response rates.^278^ Additionally, when combined with traditional antitumor agents such as chemotherapeutic drugs and immune checkpoint blockade agents, guadecitabine demonstrates a potent synergistic effect, enhancing long-term clinical benefits.^279–282^ Promisingly, its use in treating patients with AML has advanced to phase III clinical trials, indicating high response rates and comparable safety, positioning it as a promising future alternative.^279,283^ Other nucleoside DNA methylation inhibitors include CP-4200, with cellular uptake less dependent on the nucleoside transporters involved in azacytidine uptake;^284^ F-aza-T-dCyd (NSC801845), optimized structurally from T-dCyd, F-T-dCyd, and Aza-T-dCyd;^285^ DHDAC, which is less cytotoxic and more stable;^286^ NPEOC-DAC, a decitabine derivative modified at the N4 position of the azacitidine ring, displaying significantly reduced potency at low doses in inhibiting DNA methylation;^287^ and zebularine, known for its high selectivity and better biocompatibility towards pathological cells,^288^ demonstrating significant therapeutic effects not only on tumors but also on non-tumor diseases like renal fibrosis,^289^ T2DM,^290^ and NAFLD.^291^ Additionally, clofarabine, an FDA-approved purine nucleoside analog for treating pediatric AML, primarily inhibits DNA biosynthesis and the ribonucleotide reductase enzyme and has shown potential in early-stage carcinogenesis through DNMT1 inhibition.^292^

Beyond these, various non-nucleoside DNMT inhibitors have been identified. These include specific artificially synthesized inhibitors and naturally occurring agents with a demethylation function.^293,294^ Enhancements to the physical properties of these natural compounds, such as solubility and stability, benefit the development of more effective DNMT-targeted inhibitors.^295,296^ Non-nucleoside DNMT inhibitors are categorized based on their diverse mechanisms of action into competitors of S-adenosylmethionine (SAM), competitive or non-competitive inhibitors of DNMT, regulators of DNMT expression, and binders of DNA substrates.^53^ MG98 and hydralazine are the only two drugs currently in clinical trials. MG98, an antisense oligodeoxynucleotide, reduces DNMT1 mRNA levels by targeting its 3’ untranslated region. However, several clinical trials are far from satisfactory.^297^ A two-stage phase II clinical trial evaluated the antitumor efficacy of MG98 in seventeen patients with metastatic renal carcinoma.^298^ However, it failed to detect a decrease in DNMT1 activity caused by MG98, urging caution against potential side effects like transaminase elevation and fatigue from excessive dosages during intravenous administration.^298,299^ Hydralazine, a low molecular weight molecule, interacts with DNMT through a network of hydrogen bonds with arginine and glutamic acid residues.^300^ Its combination with traditional chemotherapeutic agents has been found to mitigate the progression of both hematological malignancies and solid tumors.^301–304^ In a completed phase II clinical trial, 15 patients with solid tumors qualified for the assessment of the therapeutic response to hydralazine and magnesium valproate. The majority of patients (80%), benefited from treatment and exhibited satisfactory clinical efficacy and tolerability.^304^ These findings underpin the hypothesis that epigenetic aberrations induced by chemotherapeutic agents are a primary cause of chemoresistance, providing a theoretical basis for the combined use of epigenetics-targeted drugs and chemotherapy in tumor therapy.

#### Targeting the eraser of DNA methylation: TET

Due to the significant heterogeneity in the roles that TET enzymes play across various diseases, the effectiveness of targeting TET enzymes as a therapeutic strategy depends on the specific disease or even different stages within a disease.^305^ While there are currently no epigenetic-targeted drugs that modulate TET available on the market, experimental studies and clinical trials suggest that reshaping methylation landscapes through TET inhibitors and agonists may be a viable approach to treating diseases.

##### TET inhibitors

To date, numerous small molecules have been identified that inhibit TET enzymes. Research into these TET inhibitors primarily focuses on elucidating their underlying molecular mechanisms. We categorize these inhibitors into three groups based on their distinct mechanisms of action, which will be discussed in detail below.

Auranofin, C35, and eltrombopag specifically target and bind to the catalytic domains of TET proteins, directly inhibiting their enzymatic activities. C35 exhibits potent inhibitory effects on all members of the TET family.^306,307^ In contrast, the effects of auranofin and eltrombopag are specific to TET1 and TET2, respectively.^308,309^ Notably, eltrombopag, a nonpeptidyl thrombopoietin receptor agonist approved by the US FDA for use in patients with aplastic anemia as an iron chelator.^310^ Recently, Guan et al.^309^ reported the negative effects of eltrombopag on TET2. Intriguingly, this mechanism is independent of its iron chelation properties, presenting it as a potential TET2-targeted epigenetic agent and providing new insights for epigenetics-oriented therapy.^309^ Further, well-designed studies are essential to evaluate the clinical application potential of these molecular-level discoveries.

Itaconic acid, fumarate, and succinate are in vivo synthesized metabolites that indirectly impair TET catalytic activity by competitively binding to TET2 alongside α-KG, a crucial cofactor.^311–313^ These metabolites are promising precursors for developing TET-targeted epigenetic drugs, as they are well tolerated in vivo.^314,315^ However, considerable work is necessary before clinical application, such as designing appropriate carriers that can deliver these agents directly to pathological cells, given their potential impact on the vital activities of normal cells. Additionally, synthetic compounds like dimethyloxallylglycine and TETi76, which mimic the properties of α-KG, serve as competitive inhibitors of TET.^316,317^ These agents represent a novel approach to developing TET inhibitors.

Bobcat339,^318,319^ NSC-311068,^320^ NSC-370284,^320^ and UC-514321,^320^ inhibit DNA methylation by reducing intracellular TET levels. Bobcat339 induces the degradation of TET3 directly,^318^ and its inhibitory effects on TET1 and TET2 are observed only in the presence of coordinating copper(II).^321^ NSC-370284 and UC-514321 bind directly to the DNA-binding domain of signal transducer and activator of transcription 3 (STAT3) or STAT5, transcriptional activators of TET1, leading to suppressed expression of TET1 in vivo.^320^ This mechanism has been confirmed in mouse models of AML and medulloblastoma, showing synergistic effects with standard chemotherapy.^320,322^ The therapeutic potential of these compounds in additional diseases is an exciting area for future research.

##### TET agonists

As previously discussed, TET inhibitors are highly valued for treating diseases. Conversely, research into TET agonists is also anticipated to yield promising breakthroughs and pave the way for clinical applications. Most TET agonists currently under investigation are drugs that upregulate cofactors of TET, such as vitamin C and enzymes involved in α-KG metabolism; other small molecules, including 3-nitroflavanones,^295^ retinoic acid,^323^ ioperamide hydrochloride,^324^ and mitoxantrone,^325^ are reported to directly upregulate TET expression.

Vitamin C, or ascorbate, uniquely interacts with the C-terminal catalytic domain of TET, positioning it as a novel epigenetic-modifying agent.^326,327^ As an antioxidant, it also helps maintain the divalent state of iron ions, indirectly supporting TET activity.^323^ Characterized by TET repair and increased 5hmC levels, vitamin C administration can exert therapeutic roles in various tumors and non-tumor diseases.^328–330^ Furthermore, it has been used as an adjuvant, synergizing with other immunotherapeutic or chemotherapeutic agents.^331–334^ The synergistic treatment of vitamin C with azacitidine or decitabine in clinical trials has shown positive outcomes for patients with myeloid tumors.^335,336^ However, the optimal doses, frequency, and duration of vitamin C administration remain debated, with long-term treatment and follow-up required for further investigation.^337^ Therefore, the full exploration of the therapeutic role of vitamin C as an epigenetic-modifying drug is crucial for its future clinical applications.

Inhibitors of IDH and α-KG dehydrogenase elevate α-KG levels. In tumor cells, IDH1/2 mutations lead to the production of the oncometabolite 2-HG, which competes with α-KG for binding sites on TET, potentially leading to reversible inhibition of TET proteins and dysregulation of DNA methylation levels.^338,339^ The administration of enasidinib and siRNA against IDH2 has been shown to restore the low methylated state of the genome, consistent with the reactivation of TET enzymatic activities.^340–342^ However, observations suggest that other α-KG-dependent enzymes, such as histone demethylases and prolyl hydroxylases, might play a more dominant role in the progression of IDH-mutant diseases.^343^ These findings highlight the potential for developing TET agonists based on IDH inhibitors to reshape the epigenetic landscape, warranting further investigation. Additionally, inhibiting α-KG dehydrogenase enhances α-KG levels and TET activities, restoring DNA demethylation and ameliorating the progression of T2DM and breast cancer.^344,345^ Similarly, IOX1, an inhibitor of α-KG oxygenases and a potent inhibitor of lysine demethylase 3 A (KDM3A) and KDM4A, has been found to reduce TET enzymatic activities in helper T cells, emerging as a potential epigenetic drug for various autoimmune diseases.^346^ In conclusion, α-KG represents a promising target for TET-targeted drug development that should be further explored in clinical trials.

#### Targeting the reader of DNA methylation: MBD and UHRF1

With the discovery of proteins that read methylated DNA sites, burgeoning research has aimed to identify small molecules targeting these enzymes, sparking considerable enthusiasm for developing novel therapeutic targets.

##### MBD inhibitors

The MBD protein family, critical readers of DNA methylation, consists of six members: methyl-CpG-binding protein 1 (MeCP1), MeCP2, MBD1, MBD2, MBD3, and MBD4.^347^ The aberrant activities and expression of these proteins observed in various diseases have recently positioned them as potential targets for epigenetic drugs. Current research predominantly focuses on MBD2 inhibitors, which are rapidly progressing.

The development of MBD2 inhibitors hinges on two prerequisite factors. Firstly, the knockdown of MBD2 or the application of targeted siRNA has demonstrated positive effects in tumor treatment, underscoring the therapeutic potential of targeting MBD2.^348^ Secondly, successfully elucidating MBD2’s molecular structure and associated mechanisms lays the scientific groundwork for identifying and designing inhibitory molecules. MBD2 inhibitors can be categorized into three groups based on their mechanisms of action. The first group disrupts the binding of the N-terminal MBD to methylated DNA.^349^ Through docking analysis, molecules such as CID3100583 and 8,8-ethylenebistheophylline have been identified to target the interaction between MBD2 and DNA.^350^ The second category aims to block the interaction between the C-terminal coiled-coil domain and the GATA zinc finger domain containing 2A, which has shown potent inhibitory effects on MBD2-dependent DNA methylation.^351^ However, no drugs based on this mechanism are currently in use, highlighting a gap in research that demands further exploration. The third group prevents the interaction with HDAC and the formation of the nucleosome remodeling and deacetylase complex via an intrinsically disordered region.^352–354^ Utilizing this concept, Na et al.^355^ developed a novel technique that efficiently discriminates potential compounds interacting with intrinsically disordered proteins through expanded virtual screening. This approach led to identifying two MBD2 inhibitors, ABA and APC. These findings provide a sound basis for a therapeutic strategy targeting MBD2 and advocate for more comprehensive in vivo studies to assess their efficacy and safety.

##### UHRF1 inhibitors

Ubiquitin-like with plant homeodomain (PHD) and RING finger domains 1 (UHRF1) plays a pivotal role in recruiting DNMT1 during replication, primarily through the recognition of hemimethylated DNA and the subsequent flipping of hemimethylated CpG sites via the SET and RING associated domain (SRA).^356–358^ UHRF1 also interacts with HDAC1 and facilitates the di- and tri-methylation of H3, contributing to the ubiquitination of histones and the formation of heterochromatin.^359^ The upregulation of UHRF1 has been observed in various pathologies, particularly in tumors, making it a promising target for therapeutic intervention.^360,361^

The initial identification of small inhibitors targeting the SRA domain of UHRF1 was based on structure-based screening and computational analyses. Compounds such as NSC232003,^362^ UM63,^363^ UF146,^364^ chicoric acid,^365^ have been shown to block the interaction between UHRF1 and 5mC sites, effectively preventing the proliferation of diverse cancer cell lines. Advanced screening techniques, such as nonequilibrium capillary electrophoresis of the equilibrium mixture, have facilitated the identification of proanthocyanidins and baicalein as promising inhibitors.^366^ Furthermore, Ciaco et al.^367^ reported the development of novel UHRF1 inhibitors, AMSA2 and MPB7, based on the structure of UM63. These inhibitors suppress SRA-mediated base-flipping activities without DNA intercalation and demonstrate minimal effects on non-cancer cells, offering a basis for further optimization. In addition to the SRA domain, the tandem Tudor domain and PHD domain of UHRF1, which are involved in recognizing methylated lysine and arginine residues on H3, have also become targets for inhibitor design.^368,369^ While some inhibitors targeting these domains have been reported, their effects have only been validated in vitro, and more evidence is needed before proceeding to clinical trials.^370–372^ Current research suggests that inhibiting UHRF1 alone may not be sufficient to restore gene silencing affected by hypermethylation. However, combining UHRF1 inhibitors with other epigenetic inhibitors, such as HDAC inhibitors, can lead to synergistic effects and improved therapeutic outcomes.^373,374^ Consequently, multi-target inhibitors have been developed and are emerging as clinical candidates for tumor therapy.^375–378^ Additionally, the use of small molecules that act as UHRF1 degraders, such as diosgenin and MK2206, has been explored for prostate cancer treatment, representing a novel therapeutic approach.^379,380^ Natural substances like hinokitiol have shown therapeutic effects in mouse models in a UHRF1 depletion-dependent manner although the underlying mechanisms remain to be fully elucidated and represent a direction for future research.^381^

### Epigenetics-targeted drugs and histone acetylation

The dynamic equilibrium and normal function of histone acetylation and deacetylation are regulated by the cooperative actions of the lysine acetyltransferase (KAT) and HDAC families, along with various reader proteins. Acetylation of lysine residues at the N-terminus of histones induces negative charges that trigger gene transcription, while decreased acetylation downregulates gene expression. Conversely, an imbalance in histone acetylation/deacetylation disrupts normal gene expression patterns, leading to the onset and progression of diseases. The development of related epigenetic drugs is ongoing, offering new therapeutic options for treating these conditions (Table 4).

**Table 4:** Summary of histone acetylation-targeted drugs for different diseases in clinical trials

Type	Drug	Target(s)	Condition(s)	Status/outcome(s)	Phase(s)	Other intervention(s)/drug(s)	Study ID/reference(s)
KAT inhibitor	CCS1477	P300/CBP (KAT3A/KAT3B)	NHL, MM, AML, MDS, PTCL	Recruiting	Phase I/II	With or without Pomalidomide plus Dexamethasone, or Azacitidine plus Venetoclax	NCT04068597
	CCS1477	P300/CBP (KAT3A/KAT3B)	CRPC, BC, NSCLC	Recruiting	Phase I/II	With or without Abiraterone acetate or Enzalutamide or Darolutamide or Olaparib or Atezolizumab	NCT03568656
	FT-7051	P300/CBP (KAT3A/KAT3B)	CRPC	Terminated (due to sponsors’ decision)	Phase I	—	NCT04575766
	NEO2734	P300/CBP (KAT3A/KAT3B), BET	CRPC, NUT carcinoma	Recruiting	Phase I	—	NCT05488548
	PRI-724	CBP/β-catenin	HCV-induced cirrhosis	Completed (causes liver injury in the high-dose cohort)	Phase I	—	NCT02195440^397^
	PRI-724	CBP/β-catenin	PDAC	Completed (unpublished)	Phase I	—	NCT01764477
	PRI-724	CBP/β-catenin	Solid tumors	Terminated (due to low enrollment)	Phase I	—	NCT01302405
	PRI-724	CBP/β-catenin	HIV/HCV co-induced cirrhosis	Completed (unpublished)	Phase I	—	NCT04688034
	PRI-724	CBP/β-catenin	PBC	Completed (unpublished)	Phase I	—	NCT04047160
	PRI-724	CBP/β-catenin	HIV/HCV co-induced cirrhosis	Recruiting	Phase II	—	NCT06144086
	PRI-724	CBP/β-catenin	AML, CML	Completed (unpublished)	Phase I/II	—	NCT01606579
	PRI-724	CBP/β-catenin	HCV-induced cirrhosis, HBV-induced cirrhosis	Completed (exhibits insufficient evidence of improvement in hepatic function)	Phase I/II	—	NCT03620474^396^
	PF-07248144	KAT6	HR-positive, HER2-negative BC, CRPC, NSCLC	Recruiting	Phase I	With or without Fulvestrant, or Letrozole plus Palbociclib, or Fulvestrant plus PF-07220060	NCT04606446
HDAC inhibitor	Ivaltinostat	Pan-HDAC	Malignant tumors	Active, not recruiting	Phase I	Placebo-controlled	NCT05716919
	Ivaltinostat	Pan-HDAC	Healthy volunteers	Completed (the oral formulation of Ivaltinostat is well tolerated)	Phase I	Placebo-controlled	NCT05345912^1029^
	Ivaltinostat	Pan-HDAC	PDAC	Unknown (exhibits good efficacy and an acceptable safe profile according to disclosed data)	Phase I/II	In combination with Gemcitabine and Erlotinib	NCT02737228^401^
	Ivaltinostat	Pan-HDAC	PDAC	Recruiting	Phase I/II	Capecitabine (active comparator/in combination with Ivaltinostat)	NCT05249101
	Abexinostat	Pan-HDAC	High-grade glioma	Recruiting	Phase I	In combination with Temozolomide	NCT05698524
	Abexinostat	Pan-HDAC	DLBCL, MCL	Active, not recruiting	Phase I	In combination with Ibrutinib	NCT03939182
	Abexinostat	Pan-HDAC	Melanoma, squamous cell carcinoma of head and neck, urothelial carcinoma, NSCLC	Completed (unpublished)	Phase I	In combination with Pembrolizumab	NCT03590054
	Abexinostat	Pan-HDAC	NHL, HL, MM	Completed (unpublished)	Phase I	—	NCT01149668
	Abexinostat	Pan-HDAC	Solid tumors	Active, not recruiting (exhibits good tolerability and antitumor effects according to disclosed data)	Phase I	In combination with Pazopanib	NCT01543763^404^
	Abexinostat	Pan-HDAC	NHL, HL, MM	Completed (unpublished)	Phase I	—	NCT00562224
	Abexinostat	Pan-HDAC	Malignant tumors	Completed (unpublished)	Phase I	—	NCT00473577
	Abexinostat	Pan-HDAC	MDS, AML, ALL	Terminated (due to limited clinical benefit)	Phase I	—	ISRCTN 99680465^1030^
	Abexinostat	Pan-HDAC	FL	Active, not recruiting	Phase II	—	NCT03600441
	Abexinostat	Pan-HDAC	DLBCL	Recruiting	Phase II	—	NCT03936153
	Abexinostat	Pan-HDAC	FL	Recruiting	Phase II	—	NCT03934567
	Abexinostat	Pan-HDAC	NHL	Active, not recruiting	Phase I/II	—	NCT04024696
	Abexinostat	Pan-HDAC	NHL, HL	Completed (exhibits tolerable safety and significant clinical activity in FL)	Phase I/II	—	NCT00724984^406^
	Abexinostat	Pan-HDAC	Sarcoma	Completed (exhibits manageable toxicities and tumor responses)	Phase I/II	In combination with Doxorubicin and GCSF	NCT01027910^1031^
	Abexinostat	Pan-HDAC	B cell lymphoma, CML	Completed (exhibits manageable toxicity and partial responses)	Phase I/II	—	EudraCT 2009-013691-47^405,1032^
	Abexinostat	Pan-HDAC	RCC	Recruiting	Phase III	Pazopanib (active comparator/in combination with Abexinostat)	NCT03592472
	AR-42	Pan-HDAC	Vestibular schwannoma, meningioma, acoustic neuroma, neurofibromatosis type 2	Terminated (due to a lack in drug supply)	Phase I	—	NCT02282917
	AR-42	Pan-HDAC	RCC, soft tissue sarcoma	Terminated (due to a lack in drug supply)	Phase I	In combination with Pazopanib	NCT02795819
	AR-42	Pan-HDAC	AML	Completed (exits possibilities of serious treatment-associated adverse events)	Phase I	In combination with Decitabine	NCT01798901^1033^
	AR-42	Pan-HDAC	Hematologic malignancies	Completed (exhibits tolerable safety)	Phase I	—	NCT01129193^402,1034^
	AR-42	Pan-HDAC	Plasma cell myeloma	Completed (unpublished)	Phase I	In combination with Dexamethasone and Pomalidomide	NCT02569320
	AR-42	Pan-HDAC	Neurofibromatosis type 2	Recruiting	Phase II/III	Placebo-controlled	NCT05130866
	Pracinostat	HDAC class I/II/IV	Healthy volunteers	Completed (unpublished)	Phase I	—	NCT03495934
	Pracinostat	HDAC class I/II/IV	Healthy volunteers	Completed (unpublished)	Phase I	Fasted or fed conditions	NCT02058784
	Pracinostat	HDAC class I/II/IV	Healthy volunteers	Completed (unpublished)	Phase I	In combination with Ciprofloxacin or Itraconazole	NCT02118909
	Pracinostat	HDAC class I/II/IV	Solid tumors, leukemia	Completed (unpublished)	Phase I	—	NCT01184274
	Pracinostat	HDAC class I/II/IV	Solid tumors, hematologic malignancies	Completed (exhibits safety and modest single-agent activity in hematologic malignancies)	Phase I	With or without Azacitidine	NCT00741234^1035^
	Pracinostat	HDAC class I/II/IV	AML	Completed (unpublished)	Phase I	Gemtuzumab Ozogamicin (active comparator/in combination with Pracinostat)	NCT03848754
	Pracinostat	HDAC class I/II/IV	Solid tumors	Completed (unpublished)	Phase I	—	NCT00504296
	Pracinostat	HDAC class I/II/IV	Solid tumors	Completed (exhibits good tolerability and inhibitory effects)	Phase I	—	SCS-PN0022^1036^
	Pracinostat	HDAC class I/II/IV	Solid tumors	Completed (exhibits good tolerability)	Phase I	—	^1037^
	Pracinostat	HDAC class I/II/IV	Solid tumors	Completed (exhibits good tolerability and inhibitory effects)	Phase II	—	NCT01912274^1038^
	Pracinostat	HDAC class I/II/IV	MDS	Terminated (due to sponsors’ decision)	Phase II	In combination with Azacitidine	NCT03151304
	Pracinostat	HDAC class I/II/IV	Myelofibrosis	Completed (worsening anemia and other adverse events do not support the continued development)	Phase II	In combination with Ruxolitinib and Questionnaire	NCT02267278^1039^
	Pracinostat	HDAC class I/II/IV	MDS	Completed (reduced doses exhibit improved tolerability and efficacy)	Phase II	In combination with Azacitidine and Decitabine	NCT01993641^1040^
	Pracinostat	HDAC class I/II/IV	CRPC	Completed (exhibits insufficient activity as a single agent)	Phase II	—	NCT01075308^1041^
	Pracinostat	HDAC class I/II/IV	MDS	Completed (fails to improve outcomes at the available dosing regimen)	Phase II	Azacitidine (active comparator/in combination with Pracinostat)	NCT01873703^1042^
	Pracinostat	HDAC class I/II/IV	Myeloproliferative disorders	Completed (exhibits reasonable tolerability and modest activity in myelofibrosis)	Phase II	—	NCT01200498^1043^
	Pracinostat	HDAC class I/II/IV	Sarcoma	Completed (premature stop due to the prolonged unavailability)	Phase II	—	NCT01112384^1044^
	Pracinostat	HDAC class I/II/IV	AML	Terminated (due to a lack of efficacy)	Phase III	Azacitidine (active comparator/in combination with Pracinostat)	NCT03151408^1045^
	Resminostat	HDAC class I/IIb/IV	CTCL, MF	Completed (unpublished)	Phase I	—	NCT04955340
	Resminostat	HDAC class I/IIb/IV	Biliary tract cancer, pancreatic cancer	Completed (exhibits acceptable tolerability)	Phase I	In combination with chemotherapy	JapicCTI-152864^1046^
	Resminostat	HDAC class I/IIb/IV	Solid tumors	Completed (exhibits on-target pharmacodynamic activity at dose levels ≥400 mg and signs of antitumor efficacy)	Phase I	—	^1047^
	Resminostat	HDAC class I/IIb/IV	CTCL, MF	Active, not recruiting	Phase II	Placebo-controlled	NCT02953301
	Resminostat	HDAC class I/IIb/IV	HL	Completed (exhibits acceptable safety and efficacy)	Phase II	—	NCT01037478^1048^
	Resminostat	HDAC class I/IIb/IV	HCC	Completed (exhibits early signs of efficacy and good tolerability)	Phase II	With or without Sorafenib	NCT00943449^1049^
	Resminostat	HDAC class I/IIb/IV	Biliary tract cancer	Completed (exhibits no significant improve in clinical activity)	Phase II	In combination with chemotherapy	JapicCTI-183883^1050^
	Resminostat	HDAC class I/IIb/IV	HCC	Completed (no significant efficacy advantage over sorafenib monotherapy)	Phase I/II	Sorafenib (active comparator/in combination with Resminostat)	NCT02400788^1051^
	Resminostat	HDAC class I/IIb/IV	NSCLC	Completed (fails to improve progression-free survival and increases toxicity)	Phase I/II	In combination with Docetaxel	JapicCTI-132123^1052^
	Resminostat	HDAC class I/IIb/IV	Colorectal carcinoma	Completed (unpublished)	Phase I/II	Chemotherapy (active comparator/in combination with Resminostat)	NCT01277406
	Tacedinaline	HDAC class I/II/III	Solid tumors	Completed (exhibits antitumor activity)	Phase I	In combination with Carboplatin and Paclitaxel	^1053^
	Tacedinaline	HDAC class I/II/III	Solid tumors	Completed (thrombocytopenia is the main principal dose-limiting toxicity)	Phase I	In combination with Capecitabine	^1054^
	Tacedinaline	HDAC class I/II/III	Solid tumors	Completed (exhibits preliminary efficacy and potential adverse events)	Phase I	In combination with Gemcitabine hydrochloride	^1055^
	Tacedinaline	HDAC class I/II/III	Solid tumors	Completed (exhibits preliminary efficacy and potential adverse events)	Phase I	—	^1056^
	Tacedinaline	HDAC class I/II/III	MM	Completed (unpublished)	Phase II	—	NCT00005624
	Tacedinaline	HDAC class I/II/III	Pancreatic cancer	Completed (exhibits no evidence for improving efficacy)	Phase II	In combination with Gemcitabine hydrochloride; placebo-controlled	NCT00004861^1057^
	Tacedinaline	HDAC class I/II/III	NSCLC	Completed (unpublished)	Phase III	In combination with Gemcitabine hydrochloride; placebo-controlled	NCT00005093
	FRM-0334	HDAC class I/II	Frontotemporal dementia with granulin mutation	Unknown (exhibits tolerable safety while insufficient efficacy according to disclosed data)	Phase II	Placebo-controlled	NCT02149160^1058^
	Trichostatin A	HDAC class I/II	Hematologic malignancies	Unknown	Phase I	—	NCT03838926
	Quisinostat	HDAC class I/II	Leukemia, MDS	Terminated (due to sponsors’ decision)	Phase I	—	NCT00676728
	Quisinostat	HDAC class I/II	Solid tumors, lymphomas	Completed (intermittent schedules exhibit better tolerated than continuous schedules)	Phase I	—	NCT00677105^1059^
	Quisinostat	HDAC class I/II	NSCLC, ovarian cancer	Completed (unpublished)	Phase I	In combination with Cisplatin plus Gemcitabine, or Paclitaxel plus Carboplatin	NCT02728492
	Quisinostat	HDAC class I/II	MM	Completed (exhibits efficacy and tolerable safety)	Phase I	In combination with Dexamethasone and Bortezonib	NCT01464112^1060^
	Quisinostat	HDAC class I/II	CTCL	Completed (exhibits an acceptable safety profile)	Phase II	—	NCT01486277^1061^
	Quisinostat	HDAC class I/II	Ovarian cancer	Completed (unpublished)	Phase II	In combination with Paclitaxel and Carboplatin	NCT02948075
	CXD101	HDAC class I	Malignant tumors	Completed (exhibits acceptable tolerability with efficacy in HL, T cell lymphoma, and FL)	Phase I	—	NCT01977638^1062^
	CXD101	HDAC class I	HCC	Recruiting	Phase II	In combination with Geptanolimab, Lenvatinib and Sorafenib (active comparator)	NCT05873244
	CXD101	HDAC class I	Colorectal carcinoma	Unknown (exhibits good tolerability and efficacy according to disclosed data)	Phase I/II	In combination with Nivolumab	NCT03993626^1063^
	CXD101	HDAC class I	DLBCL	Withdrawn (due to insufficent funds)	Phase I/II	In combination with Pembrolizumab	NCT03873025
	Magnesium valproate	HDAC class I	Solid tumors	Completed (exhibits the potential to overcome chemotherapy resistance)	Phase II	In combination with Hydralazine	NCT00404508^304^
	Magnesium valproate	HDAC class I	Cervical cancer	Completed (unpublished)	Phase II	In combination with Hydralazine	NCT00404326
	Magnesium valproate	HDAC class I	Colorectal carcinoma	Recruiting	Phase II	With or without Panitumumab and Cetuximab	NCT05694936
	Magnesium valproate	HDAC class I	BC	Terminated (treatment is well-tolerated)	Phase II	In combination with Hydralazine	NCT00395655^301^
	Magnesium valproate	HDAC class I	Ovarian cancer	Unknown	Phase III	In combination with Hydralazine, placebo-controlled	NCT00533299
	Magnesium valproate	HDAC class I	Cervical cancer	Completed (exhibits advantages in progression-free survival)	Phase III	In combination with Hydralazine; placebo-controlled	NCT00532818
	Magnesium valproate	HDAC class I	Cervical cancer	Unknown	Phase III	In combination with Hydralazine, Carboplatin, and Paclitaxel; placebo-controlled	NCT02446652
	OBP-801	HDAC class I	Solid tumors	Unknown (large-scale trials should be hold according to disclosed data)	Phase I	—	NCT02414516^1064^
	Nanatinostat	HDAC class I	Malignant tumors (excluding gastrointestinal tumors)	Recruiting	Phase I	With or without Valganciclovir	NCT06302140
	Nanatinostat	HDAC class I	EBV-associated lymphoma, PTCL, PTLD	Recruiting	Phase II	In combination with Valganciclovir	NCT05011058
	Nanatinostat	HDAC class I	EBV-associated lymphoma	Completed (exhibits encouraging efficacy)	Phase I/II	In combination with Valganciclovir	NCT03397706^1065^
	Nanatinostat	HDAC class I	EBV-associated solid tumors	Recruiting	Phase I/II	In combination with Valganciclovir, with or without Pembrolizumab	NCT05166577
	Entinostat	HDAC class 1	TNBC	Terminated (due to funding withdrawn)	Early phase I	—	NCT03361800
	Entinostat	HDAC class 1	Healthy volunteers	Completed (unpublished)	Phase I	—	NCT02922946
	Entinostat	HDAC class 1	Healthy volunteers; renal impairment	Completed (unpublished)	Phase I	—	NCT03192111
	Entinostat	HDAC class 1	Solid tumors	Completed (unpublished)	Phase I	Placebo-controlled	NCT02897778
	Entinostat	HDAC class 1	CRPC	Completed (exhibits an acceptable safety profile)	Phase I	In combination with Enzalutamide	NCT03829930^1066^
	Entinostat	HDAC class 1	HR-positive HER2-negative BC	Completed (exhibits reasonable safety, tolerability, and encouraging efficacy)	Phase I	In combination with Exemestane	NCT02833155^1067^
	Entinostat	HDAC class 1	MM, MDS, myeloproliferative diseases	Completed (exhibits effective inhibition on HDAC in vivo)	Phase I	—	NCT00015925^1068^
	Entinostat	HDAC class 1	Solid tumors, lymphomas	Completed (exhibits good tolerability at the studied doses)	Phase I	—	NCT00020579^1069^
	Entinostat	HDAC class 1	Healthy volunteers; renal impairment	Completed (unpublished)	Phase I	In combination with Midazolam	NCT03187015
	Entinostat	HDAC class 1	Solid tumors	Completed (unpublished)	Phase I	In combination with Pembrolizumab	NCT02909452
	Entinostat	HDAC class 1	MDS	Active, not recruiting (exhibits limited clinical efficacy and substantial toxicity according to disclosed data)	Phase I	In combination with Pembrolizumab	NCT02936752^1070^
	Entinostat	HDAC class 1	BC	Completed (unpublished)	Phase I	In combination with Capecitabine	NCT03473639
	Entinostat	HDAC class 1	Ovarian cancer, peritoneal cancer, fallopian tube cancer	Terminated (due to changes in participant landscape and other treatment availability)	Phase I	In combination with Olaparib	NCT03924245
	Entinostat	HDAC class 1	HR-positive BC	Completed (unpublished)	Phase I	In combination with Exemestane	NCT02820961
	Entinostat	HDAC class 1	HR-positive BC, NSCLC	Completed (results published along with phase II studies)	Phase I	In combination with Erlotinib and Exemestane	NCT01594398
	Entinostat	HDAC class 1	Lymphoma	Completed (exhibits tolerable safety)	Phase I	In combination with Isotretinoin	NCT00098891^1071^
	Entinostat	HDAC class 1	AML, MDS, CMML	Completed (increases toxicity in treating myeloid neoplasms)	Phase I	In combination with Azacitidine	NCT00101179^1072–1074^
	Entinostat	HDAC class 1	Healthy volunteers	Completed (unpublished)	Phase I	Dietary supplements (Omeprazole and Famotidine)	NCT02922933
	Entinostat	HDAC class 1	HR-positive BC	Completed (exhibits no additional safety concerns)	Phase I	In combination with KHK2375	NCT02623751^1075^
	Entinostat	HDAC class 1	Colorectal carcinoma	Completed (the combination is poorly tolerated without evident activity)	Phase I	In combination with Hydroxychloroquine and Regorafenib	NCT03215264^413^
	Entinostat	HDAC class 1	SCLC	Completed (further exploration should not be applied)	Phase I	In combination with Atezolizumab, Carboplatin, and Etoposide	NCT04631029^414^
	Entinostat	HDAC class 1	CNS tumors, lymphoma	Completed (exhibits good tolerability)	Phase I	—	NCT02780804^1076^
	Entinostat	HDAC class 1	Endometrial endometrioid adenocarcinoma	Completed (no immediate effect on the regulation of progesterone receptor)	Phase I	With or without Medroxyprogesterone acetate	NCT03018249^415^
	Entinostat	HDAC class 1	HER2-positive BC, TNBC	Terminated (due to slow accrual and company reasons)	Phase I	M7824 and Ado-trastuzumab emtansine (active comparator/in combination with Entinostat)	NCT04296942
	Entinostat	HDAC class 1	HER2-positive BC	Completed (exhibits acceptable tolerability and antitumor activity)	Phase I	In combination with Lapatinib ditosylate	NCT01434303^1077^
	Entinostat	HDAC class 1	ALL, ABL	Completed (exhibits less activities in relapsed/refractory patients)	Phase I	In combination with Clofarabine	NCT01132573^416^
	Entinostat	HDAC class 1	Solid tumors	Terminated (exhibits good tolerability according to disclosed data)	Phase I	In combination with Sorafenib	NCT01159301^1078^
	Entinostat	HDAC class 1	NSCLC	Terminated	Phase I	In combination with Azacitidine	NCT01886573
	Entinostat	HDAC class 1	RCC	Active, not recruiting (exhibits acceptable safety and efficacy according to disclosed data)	Phase I	In combination with Aldesleukin	NCT01038778
	Entinostat	HDAC class 1	BC	Terminated	Phase I	—	NCT00754312
	Entinostat	HDAC class 1	HR-positive BC, TNBC	Active, not recruiting (exhibits good efficacy according to disclosed data)	Phase I	In combination with Ipilimumab and Nivolumab	NCT02453620^1079^
	Entinostat	HDAC class 1	BC	Completed (exhibits acceptable safety)	Phase II	Exemestane (active comparator/in combination with Entinostat); placebo-controlled	NCT03291886^1080^
	Entinostat	HDAC class 1	Uveal melanoma	Completed (exhibits durable responses in a subset of patients)	Phase II	In combination with Pembrolizumab	NCT02697630^1081,1082^
	Entinostat	HDAC class 1	TNBC	Active, not recruiting (exhibits good tolerability but fails to meet primary endpoint according to disclosed data)	Phase II	In combination with Azacitidine	NCT01349959
	Entinostat	HDAC class 1	HL	Terminated (due to corporate decision)	Phase II	—	NCT00866333^1083^
	Entinostat	HDAC class 1	MDS, AML	Completed (increases toxicity in treating myeloid neoplasms)	Phase II	Azacitidine (active comparator/in combination with Entinostat)	NCT00313586^1072,1084^
	Entinostat	HDAC class 1	HR-positive BC	Completed (exhibits good tolerability and clinical activity)	Phase II	Exemestane (active comparator); placebo-controlled	NCT00676663^1085^
	Entinostat	HDAC class 1	Neuroendocrine tumors	Terminated (due to a lack of funding and drug supply)	Phase II	—	NCT03211988
	Entinostat	HDAC class 1	Cholangiocarcinoma, PDAC	Completed (exhibits promising efficacy)	Phase II	In combination with Nivolumab	NCT03250273
	Entinostat	HDAC class 1	Melanoma	Completed (unpublished)	Phase II	—	NCT00185302
	Entinostat	HDAC class 1	Lymphomas	Active, not recruiting	Phase II	In combination with Pembrolizumab	NCT03179930
	Entinostat	HDAC class 1	RCC	Active, not recruiting	Phase II	Interleukin-2 (active comparator/in combination with Entinostat)	NCT03501381
	Entinostat	HDAC class 1	Melanoma	Completed (exhibits preliminary antitumor effects)	Phase II	In combination with Pembrolizumab	NCT03765229
	Entinostat	HDAC class 1	Bladder cancer	Active, not recruiting	Phase II	Pembrolizumab (active comparator/in combination with Entinostat)	NCT03978624
	Entinostat	HDAC class 1	RCC	Active, not recruiting	Phase II	In combination with Nivolumab and Ipilimumab	NCT03552380
	Entinostat	HDAC class 1	AML, ALL	Completed (exhibits preliminary antitumor effects)	Phase II	In combination with Sargramostim	NCT00462605
	Entinostat	HDAC class 1	NSCLC	Terminated (due to business reasons)	Phase II	In combination with Erlotinib	NCT00750698
	Entinostat	HDAC class 1	AML	Active, not recruiting	Phase II	In combination with Azacitidine	NCT01305499
	Entinostat	HDAC class 1	NSCLC	Terminated (due to slow accrual)	Phase II	In combination with Azacitidine	NCT01207726
	Entinostat	HDAC class 1	Colon cancer, rectal cancer	Completed (exhibits preliminary antitumor effects)	Phase II	In combination with Azacitidine	NCT01105377
	Entinostat	HDAC class 1	HR-positive BC	Completed (risks of treatment-associated adverse events are high)	Phase II	In combination with Aromatase inhibitor	NCT00828854
	Entinostat	HDAC class 1	NSCLC	Completed (combination is a promising tool in future exploration)	Phase II	In combination with Azacitidine and Nivolumab; Nivolumab with or without CC-486 300 (active comparator)	NCT01928576
	Entinostat	HDAC class 1	TNBC	Terminated (due to slow accrual)	Phase II	In combination with Anastrozole	NCT01234532
	Entinostat	HDAC class 1	NSCLC	Terminated	Phase II	In combination with Azacitidine and chemotherapy; chemotherapy (active comparator)	NCT01935947
	Entinostat	HDAC class 1	NSCLC	Completed (exhibits clinically meaningful benefit)	Phase I/II	In combination with Pembrolizumab	NCT02437136^1086^
	Entinostat	HDAC class 1	CNS tumors	Recruiting	Phase I/II	In combination with Nivolumab; placebo-controlled	NCT03838042^1087^
	Entinostat	HDAC class 1	RCC	Active, not recruiting (exhibits promising clinical activities according to disclosed data)	Phase I/II	In combination with Aldesleukin	NCT01038778^1088^
	Entinostat	HDAC class 1	NSCLC	Completed (exhibits improvement in progression-free rates and overall survival)	Phase I/II	With or without Azacitidine	NCT00387465^1089^
	Entinostat	HDAC class 1	NSCLC	Completed (the combination fails to improve the outcomes)	Phase I/II	Erlotinib (active comparator/in combination with Entinostat); placebo-controlled	NCT00602030^1090^
	Entinostat	HDAC class 1	Ovarian cancer, peritoneal cancer, fallopian tube cancer	Completed (exhibits comparable efficacy and tolerability)	Phase I/II	Avelumab (active comparator/in combination with Entinostat); placebo-controlled	NCT02915523
	Entinostat	HDAC class 1	BC	Completed (exhibits clinical activity)	Phase I/II	Atezolizumab (active comparator/in combination with Entinostat); placebo-controlled	NCT02708680
	Entinostat	HDAC class 1	RCC	Suspended (due to major review underway)	Phase I/II	In combination with Atezolizumab and Bevacizumab	NCT03024437
	Entinostat	HDAC class 1	HPV-associated malignancies, small bowel cancer,colon cancer	Recruiting	Phase I/II	In combination with Bintrafusp Alfa/NHS-IL12, or NHS-IL12	NCT04708470
	Entinostat	HDAC class 1	Solid tumors	Recruiting	Phase I/II	In combination with ZEN-3694	NCT05053971
	Entinostat	HDAC class 1	Esophageal cancer	Suspended (due to revisions to design)	Phase I/II	In combination with Nivolumab, Montanide(R) ISA-51 VG Adjuvant, and H1299 Cell Lysates	NCT05898828
	Entinostat	HDAC class 1	ALL	Terminated (due to low accrual)	Phase I/II	In combination with Imatinib mesylate	NCT01383447
	Entinostat	HDAC class 1	BC	Active, not recruiting	Phase I/II	Umbrella study	NCT03280563
	Entinostat	HDAC class 1	HR-positive HER2-negative BC	Active, not recruiting (the combination fails to improve survival according to disclosed data)	Phase III	Exemestane/Goserelin/Goserelin acetate (active comparator/in combination with Entinostat); placebo-controlled	NCT02115282^1091,1092^
	Entinostat	HDAC class 1	HR-positive BC	Unknown (exhibits encouraging outcomes according to disclosed data)	Phase III	Exemestane (active comparator/in combination with Entinostat); placebo-controlled	NCT03538171^412^
	Mocetinostat	HDAC class 1	CRPC, BC, NSCLC	Terminated (due to terminated collaboration)	Phase I	In combination with Docetaxel	NCT00511576
	Mocetinostat	HDAC class 1	MDS, lymphomas	Completed (unpublished)	Phase I	Given twice weekly	NCT00324194
	Mocetinostat	HDAC class 1	MDS, lymphomas	Completed (dose-limiting toxicities of fatigue, nausea, vomiting, and diarrhea observed at higher doses)	Phase I	Given three-times weekly	NCT00324129^1093^
	Mocetinostat	HDAC class 1	NHL	Completed (unpublished)	Phase I	Given twice weekly	NCT00323934
	Mocetinostat	HDAC class 1	Squamous cell carcinoma of head and neck, squamous cell carcinoma of oral cavity	Withdrawn (due to a change in internal prioritization)	Phase I	In combination with Durvalumab	NCT02993991
	Mocetinostat	HDAC class 1	Rhabdomyosarcoma	Recruiting	Phase I	In combination with Vinorelbine	NCT04299113
	Mocetinostat	HDAC class 1	Lung cancer	Active, not recruiting	Phase I	In combination with Pembrolizumab and Guadecitabine	NCT03220477
	Mocetinostat	HDAC class 1	Melanoma	Terminated (exhibits favorable response rates but with high levels of toxicity according to disclosed data)	Phase I	In combination with Ipilimumab and Nivolumab	NCT03565406^1094^
	Mocetinostat	HDAC class 1	Urothelial carcinoma	Completed (exhibits modest clinical activity)	Phase II	—	NCT02236195^1095^
	Mocetinostat	HDAC class 1	HL	Terminated (exhibits single-agent clinical activity with manageable toxicity according to disclosed data)	Phase II	—	NCT00358982^1096^
	Mocetinostat	HDAC class 1	Lymphoma	Completed (exhibits limited single-agent activity in DLBCL and FL but long-term clinical benefit)	Phase II	—	NCT00359086^1097^
	Mocetinostat	HDAC class 1	AML, MDS	Terminated (due to terminated collaboration)	Phase II	Azacitidine (active comparator/in combination with Mocetinostat)	NCT00666497
	Mocetinostat	HDAC class 1	NHL, HL	Terminated (due to terminated collaboration)	Phase II	In combination with Azacitidine	NCT00543582
	Mocetinostat	HDAC class 1	CLL	Completed (exhibits limited activity)	Phase II	—	NCT00431873^1098^
	Mocetinostat	HDAC class 1	AML, MDS	Terminated (due to the re-evaluation of clinical development program)	Phase II	—	NCT00374296
	Mocetinostat	HDAC class 1	Leiomyosarcoma	Completed (exhibits insufficient activity)	Phase II	In combination with Gemcitabine	NCT02303262
	Mocetinostat	HDAC class 1	NSCLC	Terminated (due to sponsors’ decision)	Phase II	In combination with Nivolumab; Nivolumab with Sitravatinib or Glesatinib (active comparator)	NCT02954991
	Mocetinostat	HDAC class 1	Malignant tumors	Completed (exhibits significant toxicities in advanced pancreatic cancer)	Phase I/II	In combination with Gemcitabine	NCT00372437^1099^
	Mocetinostat	HDAC class 1	DLBCL,lymphomas	Terminated (due to slow accrual)	Phase I/II	—	NCT02282358^1100^
	Mocetinostat	HDAC class 1	NSCLC, solid tumors	Terminated (due to sponsors’ decision)	Phase I/II	In combination with Durvalumab	NCT02805660^1101^
	Mocetinostat	HDAC class 1	MDS, AML	Completed (unpublished)	Phase I/II	—	NCT00324220
	Mocetinostat	HDAC class 1	MDS	Completed (unpublished)	Phase I/II	In combination with Azacitidine	NCT02018926
	Mocetinostat	HDAC class 1	HL	Completed (exhibits preliminary clinical activity)	Phase I/II	In combination with Brentuximab vedotin	NCT02429375
	Domatinostat	LSD1/HDAC	Hematologic malignancies	Completed (exhibits safety and early signs of antitumor activity)	Phase I	—	NCT01344707^1102^
	Domatinostat	LSD1/HDAC	GastrointEstinal cancer	Unknown (exhibits an acceptable safety profile according to disclosed data)	Phase II	In combination with Avelumab	NCT03812796^1103^
	Domatinostat	LSD1/HDAC	Merkel cell carcinoma	Withdrawn (due to sponsors’ decision)	Phase II	In combination with Avelumab	NCT04874831
	Domatinostat	LSD1/HDAC	Merkel cell carcinoma	Completed (unpublished)	Phase II	In combination with Avelumab	NCT04393753
	Domatinostat	LSD1/HDAC	Melanoma	Active, not recruiting (Domatinostat addition fails to increase treatment efficacy according to disclosed data)	Phase I/II	Nivolumab (active comparator/in combination with Domatinostat); in combination with Nivolumab and Ipilimumab	NCT04133948^424^
	CUDC101	EGFR/HER2/HDAC	Solid tumors	Terminated	Phase I	—	NCT01702285
	CUDC101	EGFR/HER2/HDAC	Squamous cell carcinoma of head and neck, gastric cancer, BC, HCC, NSCLC	Completed (exhibits acceptable safety)	Phase I	—	NCT01171924
	CUDC101	EGFR/HER2/HDAC	Squamous cell carcinoma of head and neck	Completed (the combination exhibits promising feasibility)	Phase I	In combination with Cisplatin and radiation therapy	NCT01384799^420^
	CUDC101	EGFR/HER2/HDAC	Solid tumors	Completed (exhibits good tolerability and antitumor activity)	Phase I	—	NCT00728793^421^
	CUDC-907	PI3K/HDAC	Lymphoma	Completed (exhibits tolerable safety profile and durable antitumor activity)	Phase I	With or without Rituximab or Venetoclax	NCT01742988^422,1104^
	CUDC-907	PI3K/HDAC	Diffuse intrinsic pontine glioma, medulloblastoma, high-grade glioma	Active, not recruiting	Phase I	—	NCT03893487
	CUDC-907	PI3K/HDAC	TNBC, ovarian cancer, NUT carcinoma	Completed (unpublished)	Phase I	—	NCT02307240
	CUDC-907	PI3K/HDAC	CNS tumors, lymphoma	Active, not recruiting	Phase I	—	NCT02909777
	CUDC-907	PI3K/HDAC	DLBCL	Completed (exhibits preliminary antitumor effects)	Phase II	—	NCT02674750^423^
	CUDC-907	PI3K/HDAC	Cushing disease	Not yet recruiting	Phase II	—	NCT05971758
	CUDC-907	PI3K/HDAC	Thyroid cancer	Terminated (due to investigator’s reasons)	Phase II	—	NCT03002623
	Sodium phenylbutyrate	PRKCA/HDAC	MCAD deficiency	Recruiting	Phase II	—	NCT06069375
	Tinostamustine	DNA/HDAC	Melanoma	Unknown	Phase I	—	NCT03903458
	Tinostamustine	DNA/HDAC	Glioblastoma multiforme	Active, not recruiting	Phase I	—	NCT05432375
	Tinostamustine	DNA/HDAC	MM, HL, CTCL	Active, not recruiting	Phase I	—	NCT02576496
	Tinostamustine	DNA/HDAC	MGMT-promoter unmethylated glioblastoma	Completed (unpublished)	Phase I	With or without radiation therapy	NCT03452930
	Tinostamustine	DNA/HDAC	DLBCL	Withdrawn (given the safety data on drug)	Phase I	In combination with Pembrolizumab and Rituximab	NCT04279938
	Tinostamustine	DNA/HDAC	MM	Terminated (due to sponsors’ decision based on adverse events)	Phase I/II	ASCT	NCT03687125
	Tinostamustine	DNA/HDAC	SCLC, soft tissue sarcoma, TNBC, ovarian cancer, endometrial cancer	Completed (unpublished)	Phase I/II	—	NCT03345485
	GSK3117391	Esterase-sensitive motif	RA	Terminated (due to development portfolio)	Phase II	Placebo-controlled	NCT02965599
	Tefinostat	Esterase-sensitive motif	Hematologic malignancies	Completed (exhibits early signs of efficacy and absence of significant toxicity)	Phase I	—	NCT00820508^1105^
	Tefinostat	Esterase-sensitive motif	HCC	Unknown	Phase I/II	—	NCT02759601
HDAC agonist	Theophylline	Pan-HDAC	COPD	Completed (exhibits an increase in total HDAC activity and potential clinical benefit)	Phase II	With or without Fluticasone propionate	NCT00241631^1106^
	Theophylline	Pan-HDAC	COPD	Completed (fails to enhance the anti-inflammatory properties of ICS)	Phase III	In combination with ICS; placebo-controlled	NCT01599871^426^
	Theophylline	Pan-HDAC	Bronchiectasis	Completed (unpublished)	Phase IV	With or without Formoterol-budesonide	NCT01769898
	Theophylline	Pan-HDAC	COPD	Completed (exhibits an increase in total HDAC activity and potential clinical benefit)	Not applicable	With or without standard therapy	NCT00671151^1107^
Sirtuin agonist	Resveratrol	SIRT1	T2DM	Completed (H3K56ac is located in the key position between SIRT1 and T2DM)	Phase III	Placebo-controlled	NCT02244879^1108^
BET inhibitor	ZEN-3694	Pan-BET	Colorectal carcinoma	Recruiting	Phase I	In combination with Capecitabine	NCT05803382
	ZEN-3694	Pan-BET	Endometrial carcinoma	Recruiting	Phase I	In combination with Tuvusertib	NCT05950464
	ZEN-3694	Pan-BET	Platinum-resistant ovarian carcinoma	Recruiting	Phase I	In combination with Nivolumab or Nivolumab plus Ipilimumab	NCT04840589
	ZEN-3694	Pan-BET	Colorectal carcinoma	Recruiting	Phase I	In combination with Cetuximab and Encorafenib	NCT06102902
	ZEN-3694	Pan-BET	BC, NUT carcinoma	Recruiting	Phase I	In combination with Abemaciclib	NCT05372640
	ZEN-3694	Pan-BET	Ovarian cancer, solid tumors	Recruiting	Phase I	In combination with Niraparib	NCT06161493
	ZEN-3694	Pan-BET	Ovarian cancer, solid tumors	Recruiting	Phase I	In combination with Binimetinib	NCT05111561
	ZEN-3694	Pan-BET	CRPC	Completed (unpublished)	Phase I	—	NCT02705469
	ZEN-3694	Pan-BET	CRPC	Recruiting	Phase II	In combination with Enzalutamide and Pembrolizumab	NCT04471974
	ZEN-3694	Pan-BET	Solid tumors	Recruiting	Phase II	In combination with Talazoparib	NCT05327010
	ZEN-3694	Pan-BET	CRPC	Recruiting	Phase II	Enzalutamide (active comparator/in combination with ZEN-3694)	NCT04986423
	ZEN-3694	Pan-BET	Squamous cell lung cancer	Recruiting	Phase II	—	NCT05607108
	ZEN-3694	Pan-BET	Ovarian cancer, peritoneal cancer, fallopian tube cancer	Recruiting	Phase II	In combination with Talazoparib	NCT05071937
	ZEN-3694	Pan-BET	TNBC	Terminated (based on results from an interim futility analysis and not due to safety concerns)	Phase II	In combination with Talazoparib	NCT03901469^485^
	ZEN-3694	Pan-BET	NUT carcinoma	Recruiting	Phase I/II	In combination with Cisplatin and Etoposide	NCT05019716
	ZEN-3694	Pan-BET	Solid tumors, lymphomas	Recruiting	Phase I/II	In combination with Entinostat	NCT05053971
	ZEN-3694	Pan-BET	CRPC	Completed (exhibits acceptable safety and efficacy)	Phase I/II	In combination with Enzalutamide	NCT02711956^486^
	ZEN-3694	Pan-BET	CRPC	Enrolling by invitation	Phase I/II	In combination with Enzalutamide	NCT04145375
	Trotabresib	Pan-BET	HER2-positive BC with CNS metastasis and leptomeningeal disease	Withdrawn (due to sponsors’ decision)	Phase I	In combination with Vinorelbine and radiation therapy	NCT06137651
	Trotabresib	Pan-BET	Astrocytoma, glioblastoma	Terminated (due to a change in business objectives)	Phase I	—	NCT04047303^1109^
	Trotabresib	Pan-BET	Solid tumors, NHL	Active, not recruiting (exhibits good tolerability and single-agent activity in advanced solid tumors according to disclosed data)	Phase I	—	NCT03220347^1110,1111^
	Trotabresib	Pan-BET	Pediatric cancer	Active, not recruiting	Phase I	BMS-986158 (active comparator)	NCT03936465
	Trotabresib	Pan-BET	Solid tumors	Withdrawn (due to a change in business objectives)	Phase I	—	NCT05678283
	Trotabresib	Pan-BET	Glioblastoma	Active, not recruiting	Phase I	In combination with Temozolomide and radiation therapy; radiation therapy (active comparator)	NCT04324840
	Alobresib	Pan-BET	Solid tumors, lymphomas, HR-positive BC	Completed (reports pharmacokinetics and pharmacodynamics)	Phase I	With or without Exemestane or Fulvestrant	NCT02392611
	Alobresib	Pan-BET	CRPC	Terminated (exhibits acceptable tolerability)	Phase I/II	With or without Enzalutamide	NCT02607228
	GSK3358699	Pan-BET	Healthy volunteers	Terminated (due to strategic reasons)	Phase I	Placebo-controlled	NCT03426995^1112^
	TEN-010	Pan-BET	AML, MDS	Completed (exhibits insufficient activity as a single agent)	Phase I	—	NCT02308761^1113^
	TEN-010	Pan-BET	Solid tumors	Completed (exhibits evidence of target engagement and preliminary single-agent activity)	Phase I	—	NCT01987362^1114^
	TEN-010	Pan-BET	Ovarian cancer, TNBC	Terminated (due to development portfolio)	Phase I	In combination with Atezolizumab	NCT03292172
	TEN-010	Pan-BET	MM	Completed (exhibits infrequent and short duration of clinical response rates)	Phase I	With or without Daratumumab	NCT03068351^1115^
	TEN-010	Pan-BET	DLBCL	Completed (unpublished)	Phase I	In combination with Venetoclax and Rituximab	NCT03255096^1116^
	ODM-207	Pan-BET	Solid tumors	Completed (exhibits safety at doses up to 2 mg/kg but has a narrow therapeutic window)	Phase I/II	—	NCT03035591^1117^
	ABBV-744	Pan-BET	Myelofibrosis	Terminated (due to strategic reasons)	Phase I	—	NCT03360006
	ABBV-744	Pan-BET	AML	Active, not recruiting	Phase I	With or without Ruxolitinib or Navitoclax	NCT04454658
	Birabresib	BRD2/3/4	Solid tumors	Completed (exhibits a favorable safety profile with clinical activity in NUT carcinoma)	Phase I	—	NCT02259114^1118^
	Birabresib	BRD2/3/4	AML, ALL, DLBCL, MM	Completed (exhibits evidence of clinical activity though does not meet objective response criteria in non-leukemia cohort)	Phase I	—	NCT01713582^1119,1120^
	Birabresib	BRD2/3/4	AML, DLBCL	Terminated (due to limited efficacy)	Phase I	—	NCT02698189
	Birabresib	BRD2/3/4	NUT carcinoma, TNBC, NSCLC, CRPC	Terminated (due to limited efficacy)	Phase I	—	NCT02698176
	Birabresib	BRD2/3/4	GBM	Terminated (due to limited efficacy)	Phase II	—	NCT02296476
	Birabresib	BRD2/3/4	AML	Withdrawn	Phase I/II	Azacitidine (active comparator/in combination with Birabresib)	NCT02303782
	Molibresib	BRD2/3/4	Solid tumors, lymphomas	Withdrawn (due to disapproved protocal)	Phase I	In combination with Entinostat	NCT03925428
	Molibresib	BRD2/3/4	NUT carcinoma, solid tumors	Completed (exhibits acceptable safety)	Phase I	—	NCT01587703^1121,1122^
	Molibresib	BRD2/3/4	HR-positive HER2-negative BC	Terminated (due to meeting protocol-defined futility)	Phase I	In combination with Fulvestrant; placebo-controlled	NCT02964507^1123^
	Molibresib	BRD2/3/4	CRPC	Terminated (due to meeting protocol-defined futility)	Phase I	In combination with Abiraterone plus Prednisone or Enzalutamide	NCT03150056
	Molibresib	BRD2/3/4	Healthy volunteers	Completed (CYP3A enzymes play a major role in the elimination of Molibresib)	Phase I	In combination with Itraconazole or Rifampicin	NCT02706535^1124^
	Molibresib	BRD2/3/4	Hematologic malignancies	Completed (exhibits antitumor activity but is limited by gastrointestinal and thrombocytopenia toxicities)	Phase II	—	NCT01943851^1125^
	Molibresib	BRD2/3/4	SCLC, solid tumors	Withdrawn (due to reevaluation)	Phase II	In combination with Trametinib	NCT03266159
	Molibresib	BRD2/3/4	NUT carcinoma	Withdrawn (due to disapproved protocal)	Phase I/II	In combination with Cisplatin, Etoposide, and Etoposide phosphate	NCT04116359
	Mivebresib	BRD2/4/T	Myelofibrosis	Terminated (due to strategic reasons)	Phase I	WIth or without Ruxolitinib or Navitoclax	NCT04480086
	Mivebresib	BRD2/4/T	BC, NSCLC, AML, MM PC, SCLC, NHL	Completed (exhibits good tolerability and potential efficacy in advanced solid tumors)	Phase I	With or without Venetoclax	NCT02391480^1126,1127^
	BAY1238097	BRD4-BD1; BRD2/3	Malignant tumors	Terminated	Phase I	—	NCT02369029
	PLX-2853	BRD4	AML, MDS	Completed (unpublished)	Phase I	—	NCT03787498
	PLX-2853	BRD4	Malignant tumors	Completed (unpublished)	Phase I	—	NCT03297424
	PLX-2853	BRD4	CRPC	Terminated (due to business realignment)	Phase I/II	In combination with Abiraterone acetate plus Prednisone, or Olaparib	NCT04556617
	PLX-2853	BRD4	Uveal melanoma	Withdrawn (drug company has withdrawn support)	Phase I/II	In combination with Trametinib	NCT05677373
	PLX-2853	BRD4	Platinum-resistant ovarian carcinoma	Terminated (due to business realignment)	Phase I/II	With or without Carboplatin	NCT04493619
	INCB054329	BRD4	Solid tumors, hematologic malignancies	Terminated (due to interindividual pharmacokinetic variability)	Phase I/II	—	NCT02431260^1128^
	SYHA1801	BRD4	Solid tumors	Unknown	Phase I	—	NCT04309968
	CC-95775	BRD4	AML, MDS, NHL	Completed (unpublished)	Phase I	With or without Azacitidine	NCT02543879
	CC-95775	BRD4	Solid tumors, NHL	Completed (unpublished)	Phase I	—	NCT04089527
	PLX51107	BRD4	Solid tumors, hematologic malignancies	Terminated (due to business reasons)	Phase I	—	NCT02683395
	PLX51107	BRD4	AML, MDS	Completed (unpublished)	Phase I	In combination with Azacitidine	NCT04022785
	PLX51107	BRD4	Acute GVHD	Terminated (due to sponsors’ decision)	Phase I/II	—	NCT04910152
	BMS-986158	BRD4	Pediatric Cancer	Active, not recruiting	Phase I	BMS-986378 (active comparator)	NCT03936465
	BMS-986158	BRD4	Myelofibrosis	Active, not recruiting	Phase I/II	In combination with Ruxolitinib or Fedratinib	NCT04817007
	BMS-986158	BRD4	Solid tumors, hematologic malignancies	Completed (exhibits insufficient evidence of improvement)	Phase I/II	With or without Nivolumab	NCT02419417
	BMS-986158	BRD4	MM	Recruiting	Phase I/II	In combination with Tazemetostat plus Dexamethasone or BMS-986158 plus Dexamethasone or Trametinib plus Dexamethasone, or Dexamethasone	NCT05372354
	AZD5153	BRD4	Solid tumors, lymphomas	Completed (exhibits tolerable safety as monotherapy and in combination)	Phase I	With or without Olaparib	NCT03205176^1129^
	AZD5153	BRD4	NHL, DLBCL	Completed (unpublished)	Phase I	In combination with Acalabrutinib; Acalabrutinib, Rituximab, plus Hu5F9-G4 (active comparator)	NCT03527147
	AZD5153	BRD4	AML	Recruiting	Phase I/II	Umbrella study	NCT03013998
	BI894999	BRD4-BD1/BD2	Malignant tumors, NUT carcinoma	Completed (exhibits preliminary antitumor effects and reports the maximum tolerated dose at different cohorts)	Phase I	—	NCT02516553^1130^
	Apabetalone	BRD4-BD2	PAH	Completed (exhibits good tolerability and clinical benefits)	Early phase I	—	NCT03655704^1131^
	Apabetalone	BRD4-BD2	PAH	Not yet recruiting	Phase II	Placebo-controlled	NCT04915300
	Apabetalone	BRD4-BD2	Atherosclerosis, CAD	Completed (exhibits good tolerability)	Phase II	Placebo-controlled	NCT01058018^1132^
	Apabetalone	BRD4-BD2	T2DM	Completed (exhibits potential to against the disease development)	Phase II	Placebo-controlled	NCT01728467^1133^
	Apabetalone	BRD4-BD2	Dyslipidemia, CAD	Completed (exhibits good tolerability)	Phase II	Placebo-controlled	NCT01423188^1134^
	Apabetalone	BRD4-BD2	CAD	Completed (exhibits no significant improvement)	Phase II	Placebo-controlled	NCT01067820^1134–1137^
	Apabetalone	BRD4-BD2	Dyslipidemia, CAD	Terminated	Phase II	In combination with Rosuvastatin or Atorvastatin	NCT01863225
	Apabetalone	BRD4-BD2	Chronic kidney failure	Not yet recruiting	Phase I/II	Placebo-controlled	NCT03160430
	Apabetalone	BRD4-BD2	Fabry disease	Withdrawn (due to changed development priorities)	Phase I/II	—	NCT03228940
	Apabetalone	BRD4-BD2	Healthy volunteers, dyslipidemia, atherosclerosis, CAD	Completed (unpublished)	Phase I/II	Placebo-controlled	NCT00768274
	Apabetalone	BRD4-BD2	T2DM, CAD	Completed (fails to reduce the risk of major adverse cardiovascular events)	Phase III	In combination with Rosuvastatin or Atorvastatin; placebo-controlled	NCT02586155^477,1137–1142^
	Apabetalone	BRD4-BD2	COVID-19 infection	Terminated (fails to recruit subjects)	Phase II/III	Standard of care (active comparator/in combination with Apabetalone)	NCT04894266
	NUV-868	BRD4-BD2	Solid tumors	Recruiting	Phase I/II	With or without Olaparib or Enzalutamide	NCT05252390
	Pelabresib	BRD4-BD1	Malignant tumors	Completed (unpublished)	Phase I	—	NCT05391022
	Pelabresib	BRD4-BD1	Lymphoma	Completed (exhibits good tolerability and inhibitory effects)	Phase I	—	NCT01949883^1143^
	Pelabresib	BRD4-BD1	MM	Completed (unpublished)	Phase I	—	NCT02157636
	Pelabresib	BRD4-BD1	Peripheral nerve tumor	Withdrawn (due to a lack of enrollment)	Phase II	—	NCT02986919
	Pelabresib	BRD4-BD1	Myelofibrosis, AML, MDS, myeloproliferative disorders	Active, not recruiting (exhibits potential disease-modifying activity in myelofibrosis according to disclosed data)	Phase I/II	With or without Ruxolitinib	NCT02158858^484,1144^
	Pelabresib	BRD4-BD1	Malignant tumors	Not yet recruiting	Phase III	Placebo-controlled	NCT06401356
	Pelabresib	BRD4-BD1	Myelofibrosis	Active, not recruiting	Phase III	In combination with Ruxolitinib, placebo-controlled	NCT04603495
	TQB3617	BET	Malignant tumors	Unknown	Phase I	—	NCT05110807
	TQB3617	BET	Myelofibrosis	Recruiting	Phase I/II	In combination with TQ05105	NCT06122831
	TQB3617	BET	Esophageal squamous cell carcinoma	Not yet recruiting	Phase I/II	In combination with TQB2618, Paclitaxel, and Cisplatin, or Paclitaxel plus Cisplatin, or TQB2618 plus Penpulimab	NCT05834543
	EP31670	P300(CBP)/BET	CRPC, NUT carcinoma	Recruiting	Phase I	—	NCT05488548^1145^

*ABL* acute biphenotypic leukemia, *ALL* acute lymphoblastic leukemia, *AML* acute myeloid leukemia, *ASCT* autologous stem cell transplant, *BC* breast cancer, *BD* bromodomain, *BET* bromodomain and extraterminal domain, *BRD* bromodomain containing protein, *CAD* coronary artery disease, *CBP* cyclic adenosine monophosphate-responsive element-binding protein (CREB)-binding protein, *CLL* chronic lymphocytic leukemia, *CML* chronic myeloid leukemia, *CMML* chronic myelomonocytic leukemia, *CNS* central nervous system, *COPD* chronic obstructive pulmonary disease, *COVID-19* corona virus disease 2019, *CRPC* Castration-resistant prostate cancer, *CTCL* cutaneous T cell lymphoma, *CYP3A* cytochrome P4503A, *DLBCL* diffuse large B cell lymphoma, *EBV* Epstein-Barr virus, *EGFR* epidermal growth factor receptor, *FL* follicular lymphoma, *GBM* glioblastoma multiforme, *GCSF* granulocyte colony-stimulating factor, *GVHD* graft versus host disease, *HCC* hepatocellular carcinoma, *HDAC* histone deacetylase, *HCV* hepatitis C virus, *HER2* human epidermal growth factor receptor 2, *HIV* human immunodeficiency virus, *H3K56ac* histone 3 acetylation at the 56 lysine residue, *HL* Hodgkin lymphoma, *HPV* human papilloma virus, *HR* hormone receptor, *ICS* inhaled corticosteroid, *KAT* lysine acetyltransferase, *LSD1* lysine specific demethylase 1, *MCL* mantle cell lymphoma, *MCAD* medium-chain acyl-CoA dehydrogenase, *MDS* myelodysplastic syndrome, *MF* mycosis fungoides, *MGMT* O^6^-methylguanine-DNA methyltransferase, *MM* multiple myeloma, *NHL* non-Hodgkin lymphoma, *NSCLC* non-small cell lung cancer, *NUT* nuclear protein in testis carcinoma, *PAH* pulmonary arterial hypertension, *PBC* primary biliary cholangitis, *PDAC* pancreatic ductal adenocarcinoma, *PI3K* phosphoinositide 3-kinase, *PRKCA* protein kinase C alpha, *PTCL* peripheral T cell lymphoma, *PTLD* post-transplant lymphoproliferative disorder, *RA* rheumatoid arthritis, *RCC* renal cell carcinoma, *SIRT1* Sirtuin1, *T2DM* type 2 diabetes, *TNBC* triple-negative breast cancer

#### Targeting the writer of histone acetylation: KAT

Histone acetylation serves various functions within cells. However, aberrant acetylation catalyzed by KAT can trigger the pathogenesis of various human diseases, including neurodegenerative diseases, metabolic diseases, and tumors.^382–385^ Developing epigenetic drugs that regulate KAT activity is a promising avenue for treating these diseases.

##### KAT inhibitors

Numerous inhibitors targeting KAT have been developed, primarily focusing on the P300/CBP, GNAT/PCAF, and MYST classes.^386–388^ These enzymes contain two accessible domains: the acetyl-lysine binding BD and the catalytic domain, utilizing acetyl CoA as a cofactor to transfer acetyl groups. Thus, designed inhibitors can target the enzymatic activity and the binding sites for acetyl CoA. Moreover, research into KAT degraders is advancing with the advent of proteolysis-targeting chimeras (PROTACs). These degraders are ternary complexes comprising ligands for targeted proteins and E3 ubiquitin ligase, along with a connecting linker, allowing targeted degradation via a ubiquitination-dependent method.^389^

Five KAT inhibitors have entered clinical practice, including CCS1477, FT-7051, NEO2734, PRI-724, and PF-07248144. CCS1477 targets the P300/CBP (KAT3A/KAT3B) via interaction with the BD fragment, exhibiting potent antitumor effects in cancer cell lines and animal models.^390,391^ This has led to its application in monotherapy and in combination with chemotherapeutic drugs in phase I and II clinical trials (NCT04068597, NCT03568656), with the potential to improve therapeutic strategies for both advanced solid tumors and hematological malignancies. FT-7051, another P300/CBP inhibitor targeting the BD domain, has been shown to reduce H3K27Ac at specific promoter sites and is currently under study in a phase I clinical trial for patients with hormone receptor-positive prostate cancer.^392^ NEO2734, a dual P300/CBP and BET inhibitor, demonstrates therapeutic potential comparable to the combination of a BET inhibitor and a P300/CBP inhibitor in treating certain cancers.^393,394^ It is currently being evaluated in a phase I clinical trial focusing on castration-resistant prostate cancer and other advanced solid tumors to assess its maximum tolerated oral dose (NCT05488548). PRI-724 effectively disrupts the interaction between β-catenin and CBP, ameliorating various diseases by inhibiting the Wnt/β-catenin signaling pathway.^395^ Its safety, tolerability, and antifibrotic effects have been further evaluated in two completed clinical trials among patients with hepatitis C virus (HCV)- and HBV-induced cirrhosis.^396,397^ However, PRI-724 fails to exhibit sufficient evidence of improvement in hepatic function according to existing data.^396^ Lastly, PF-07248144, a selective inhibitor of KAT6 (a member of the MOZ/MORF family), is currently under clinical investigation for the treatment of advanced breast cancer (NCT04606446). In summary, KATs represent compelling targets for therapeutic strategies, and developing novel and high-quality inhibitors with improved safety and efficacy is reaching an exciting phase.

#### Targeting the eraser of histone acetylation: HDAC

Zn^2+^-dependent classical HDACs and nicotinamide adenine dinucleotide (NAD)^+^-dependent HDACs (sirtuins) are crucial for dynamic deacetylation modifications on histones and non-histone proteins, playing significant roles in ontogeny and tumorigenesis. Despite the HDAC family’s broad substrate range in vitro, their specific subcellular localization restricts their biological functions and target proteins. Using inhibitors and agonists of HDACs and sirtuins to correct abnormal acetylation patterns is a promising therapeutic strategy.^398,399^ Notably, the therapeutic effectiveness of these interventions, in an epigenetic-dependent manner, hinges on the participation of target enzymes in histone deacetylation.

##### HDAC inhibitors

HDAC inhibitors are designed based on the spatial structure of their targets, characterized by highly conserved and homologous catalytic domains, including a catalytic channel, a zinc cation, and secondary pockets. Most HDAC inhibitors consist of a surface binding region, binding to the catalytic channel, and a zinc-binding group along with the linker, chelating the zinc ion.^400^ Four main categories of HDAC inhibitors are extensively studied: pan-inhibitors, selective inhibitors, multitarget agents, and PROTACs-based HDAC degraders.^398^ We will now discuss the recent applications of these HDAC inhibitors in clinical trials.

Four FDA-approved HDAC inhibitors—vorinostat, romidepsin, belinostat, and panobinostat—demonstrate a pan-inhibitory effect on almost all HDAC members and have made significant progress in treating some hematological malignancies. This success has fueled enthusiasm for developing additional pan-inhibitors to expand the clinical indications of these drugs. Currently, several pan-inhibitors, including ivaltinostat (CG200745), AR-42, abexinostat (PCI-24781), bisthianostat (CF-367), and sodium valproate, are under clinical trials for various tumors. The phase II study on ivaltinostat for advanced pancreatic ductal adenocarcinoma reports enhanced sensitivity of tumor cells to gemcitabine and erlotinib, presenting it as a potential treatment option.^401^ Another phase II study aims to determine the maximum tolerated dose and dose-limiting toxicity of ivaltinostat in combination with gemcitabine and erlotinib in patients with advanced pancreatic cancer, although clinical data have not been publicly disclosed, suggesting potential challenges (NCT02737228). In phase I trials, single-agent AR-42 has shown promise in treating type 2-associated meningiomas and schwannomas, with patients exhibiting good tolerance and therapeutic potential.^402,403^ However, a phase I trial focusing on advanced sarcoma and kidney cancer was terminated early due to observed dose-limiting toxicities in six patients (NCT02795819). Abexinostat, an oral small pan-inhibitor, whether used as monotherapy or in combination with chemotherapeutic agents, has shown promising therapeutic potential and acceptable safety profiles in solid tumors and hematological malignancies.^404–406^ Notably, a phase III study on abexinostat for locally advanced or metastatic renal cell carcinoma is ongoing in various regions, highlighting its potential as a clinical candidate (NCT03592472). Bisthianostat, a novel bisthiazole-derived pan-HDAC inhibitor, was studied in phase 1a clinical trial.^407^ Although preliminary data suggested modest efficacy and tolerability as a single agent in patients with R/R MM, this study has been terminated for undisclosed reasons (NCT03618602).

The non-selective inhibition characteristic of pan-HDAC inhibitors often leads to a broad spectrum of adverse effects and off-target toxicities, which restrict their widespread clinical application.^408^ Given the diverse roles of different HDAC classes, there is increasing interest in developing selective HDAC inhibitors, viewed as promising alternatives with better tolerance.^409,410^ However, due to a lack of evidence supporting the involvement of HDAC5/6/7/8/10 in histone deacetylation, selective inhibitors targeting these enzymes are not typically included in summaries of epigenetic-targeted drugs.^411^ Chidamide and givinostat, both FDA-approved selective inhibitors, have shown superior therapeutic efficacy and safety profiles. Givinostat, in particular, has promisingly expanded the clinical indications of HDAC inhibitors to include non-tumor diseases. Beyond these marketed drugs, several selective inhibitors have entered clinical practice. Notably, four such inhibitors are undergoing phase III clinical trials: pracinostat (NCT03151408), entinostat,^412^ magnesium valproate (NCT00533299), and tacedinaline (NCT00005093). Among these, only the phase III trial of entinostat combined with exemestane in treating hormone receptor-positive advanced breast cancer has shown satisfactory efficacy and manageable toxicities.^412^ However, among patients with other types of tumors such as colorectal carcinoma, lung cancer, endometrial endometrioid adenocarcinoma, and hematologic malignancies, entinostat fails to improve survival despite exhibiting good clinical efficacy.^413–416^ Importantly, according to an early-terminated, phase I clinical trial that evaluated the combination of entinostat, hydroxychloroquine, and regorafenib, the drug regimen among patients with metastatic colorectal carcinoma was poorly tolerated, with higher risks of weight loss, fatigue, and anorexia.^413^ Despite these advancements, selective inhibitors still face significant developmental challenges as they emerge as the next generation of HDAC inhibitors.

Recently, multitarget agents-based HDAC inhibitors have gained attention and have been posited to perform versatile roles in disease treatment.^417–419^ Various such agents, including those dual-targeting HDACs and kinases, receptors, DNA, transcriptional factors, and apoptosis-related proteins, are under preclinical investigation. Examples include curcumin (previously mentioned as a DNMT/METTL3 inhibitor), CUDC-101, tinostamustine (EDO-S101), fimepinostat (CUDC-907), domatinostat (4SC-202), and dacinostat (NVP-LAQ824, LAQ824), all of which are involved in various clinical trials.^398^ It has been widely reported that these multitarget agents enhance safety and reduce drug resistance in various diseases, both as monotherapy and in combination with radiotherapy or chemotherapy.^420–423^ However, emerging research offers a contrasting perspective. In a recent phase Ib clinical trial focusing on domatinostat (a dual inhibitor of LSD1/HDAC) in patients with advanced melanoma, the drug failed to enhance the efficacy of treatments targeting anti-PD-1 and cytotoxic T lymphocyte-associated antigen-4 (CTLA-4) while unexpected severe skin toxicity was observed.^424^ Furthermore, as current knowledge about these multitarget agents is still primarily derived from early-stage clinical trials, extensive investigations are necessary to validate their therapeutic value in a broader population.

##### HDAC agonists

While HDAC inhibitors have been extensively studied, research on HDAC agonists has been less prevalent. However, the therapeutic value of these agents in specific diseases has been demonstrated. Theophylline, used initially as an inhibitor of phosphodiesterase and adenosine receptors in treating asthma and chronic obstructive pulmonary disease, has recently shown activated effects on HDAC in low doses. These effects synergistically enhance the anti-inflammatory properties of cortisol in asthma and chronic obstructive pulmonary disease treatments.^425^ Nonetheless, a phase III clinical study revealed that additional administration of low-dose theophylline, along with inhaled long-acting β_2_-agonists and corticosteroids, failed to enhance HDAC activity in vivo. This left no significant difference from the anti-inflammatory properties of standard therapy.^426^

##### SIRT inhibitors and agonists

The sirtuin family’s role in developing various diseases, including inflammation, cardiovascular diseases, metabolic disorders, neurodegenerative diseases, and cancer, underscores the importance of exploring molecules that modulate their activity. Notably, SIRT2 is involved in the deacetylation of histone H4 during the G2/M transition and mitosis but is predominantly found in the cytosol, where it participates in non-histone deacetylation.^427,428^ SIRT3-5 are mainly located in mitochondria and possess a mitochondrial targeting sequence,^429^ while SIRT7 is primarily found in the nucleus, though fewer studies have addressed molecules that regulate its activity.^430^ Consequently, the inhibitors and agonists of SIRT1 and SIRT6 are highlighted as promising epigenetics-targeted drugs with significant potential.

From a mechanistic perspective, five classes of inhibitors of SIRT1 have been identified: First, competitive inhibitors that vie for acylated substrates at the binding sites, exemplified by natural products such as sirtinol, splitomicin, and cambinol analogs;^431,432^ Second, competitive inhibitors that challenge NAD^+^ for binding sites, including selisistat (EX-527) and Sosbo;^433–435^ Third, adenosine analogs such as Ro 31-8220;^436,437^ Further, binary inhibitors that compete with substrates or cofactors at separate binding sites, represented by ELT-31, a non-selective SIRT1-3 inhibitor;^438^ And the last, non-competitive inhibitors, including nicotinamide and its analogs, tenovins, thioacetyl-lysine peptides, and other small peptides.^439–441^ EX527, one of the few sirtuin inhibitors in clinical use, has demonstrated antitumor effects in vitro and potential as an adjunct in tumor therapy.^442^ Additionally, it has proven safe and well-tolerated within the therapeutic concentration range for treating neurodegenerative diseases.^443^ However, minimal therapeutic effects were observed in a phase I clinical trial focusing on early-stage HD, with further large-scale trials needed to explore its clinical potential.^444^ Ongoing research also investigates EX-527’s potential roles in improving other metabolic diseases, including endotoxemia,^445^ diabetic nephropathy,^446^ and infertility (NCT04184323) remains ongoing. Utilizing computational tools to predict potential allosteric sites has led to the identification of some allosteric SIRT6 inhibitors, including JYQ-42,^447^ compound 11e,^448^ and a pyrrole-pyridinimidazole derivative.^449,450^ Given that histone deacetylation catalyzed by SIRT6 promotes both tumor and non-tumor diseases, designing and in-depth study of these allosteric SIRT6 inhibitors represent a promising research field for human disease treatment.^451,452^

The agonists of the sirtuin family have been widely studied since the discovery of the first SIRT1 agonist, resveratrol, in 2003.^453^ The initially discovered sirtuin agonists mainly upregulate target enzyme activity through allosteric effects and are classified into two primary categories based on their origins. The first category comprises natural products extracted from plants, including resveratrol and other polyphenolic molecules.^454–459^ The second category consists of synthesized agonists that exhibit greater selectivity, focusing particularly on SIRT1, such as SRT1460, SRT1720, SRT2104, SRT2183, and SRT3025, as well as those targeting SIRT6, including UBCS039 and MDL-800.^460^ Additionally, compounds that are suggested to upregulate SIRT1 expression have been identified. These are predominantly involved in the activation of the mitogen-activated protein kinase pathway, including DDIT3,^461^ phloretin,^462^ puerarin,^463^ and atractylenolide III.^464^ Other reported agonists include include astragaloside intravenous,^465^ hesperidin,^466^ caffeic acid phenethyl ester,^467^ agomelatine,^468^ ligustilide,^469^ tanshinone IIA,^470^ and farnesol.^471^ These studies emphasize the role of SIRT1 activation in the deacetylation of various non-histones. However, the potential of these molecules as epigenetics-targeted drugs requires further exploration. Moreover, NAD^+^-enhancing molecules, which promote NAD^+^ generation or rescue their levels, represent a novel class of sirtuin agonists. These molecules may activate all sirtuin members with a single compound, attracting considerable attention.^472^ Given the diverse roles of NAD^+^ in multiple signaling pathways, additional discussion is needed to determine whether drugs that increase NAD^+^ levels have therapeutic effects in a sirtuin-dependent manner.

#### Targeting the reader of histone acetylation: BET, YEATS, and PHD

BET, YAF9, eleven-nineteen-leukemia protein (ENL), acute lymphocytic leukemia 1-fused gene from chromosome 9 protein (AF9), TAF14, and SAS5 (YEATS) domain, and PHD finger proteins are critical “readers” of acetylated residues and play essential roles as epigenetics-modifying enzymes in the transcription of downstream target genes. Drugs that target aberrant levels or activities of acetyl-recognition domain-containing proteins represent an emerging class of therapies for various diseases.

##### BET inhibitors

In recent years, a substantial number of BET inhibitors have been identified, encompassing pan-inhibitors, BD1/BD2 selective inhibitors, dual inhibitors of kinases and BET, and PROTACs-based inhibitors.^60,473,474^ From a therapeutic standpoint, BET inhibitors are primarily developed for treating tumors, with some also showing potential in non-tumor diseases, such as VYN-201 and VYN-202, among other BD2 selective inhibitors.^475^

More than twenty BET inhibitors have progressed to clinical trials, with several undergoing advanced phase evaluations. Notably, apabetalone (RVX-208) stands out as the sole BD2-selective inhibitor in phase III trials for addressing cardiovascular diseases and metabolic disorders such as T2DM.^476–478^ Apabetalone demonstrates significant anti-inflammatory properties, providing a robust scientific basis for its ongoing clinical evaluation.^479–481^ Another BET inhibitor, pelabresib (CPI-0610), has also reached phase III trials and shows promise as a treatment for myelofibrosis.^482^ In earlier phase II studies, pelabresib combined with ruxolitinib surpassed the efficacy of Janus kinase inhibitor monotherapy in treating symptomatic myelofibrosis while maintaining a manageable safety profile.^483,484^ ZEN-3694, a leading pan-BET inhibitor, has advanced to phase II trials, demonstrating efficacy when used with cyclin-dependent kinases (CDKs) inhibitors and conventional chemotherapy in cancer treatment.^485^ Preliminary phase Ib/IIa trials indicate that ZEN-3694, in combination with enzalutamide, is beneficial for patients with metastatic castration-resistant prostate cancer.^486^ An increasing number of trials focusing on ZEN-3694 are currently underway, which will provide further data to evaluate its therapeutic promise. Furthermore, recent reports highlight dinaciclib, a well-known CDK inhibitor, now recognized for its novel activity in BET suppression.^487^ The dual inhibitory capability of dinaciclib presents a potential strategy to counteract BET resistance in AML treatment.^487^ These developments underscore the potential of these drugs to achieve market approval for broad clinical use. However, some BET inhibitors as single-agent therapies have shown mixed outcomes in clinical trials for distinct cancer settings, despite their excellent results in preclinical models.^488^ For example, several phases 1 and 2 clinical trials investigating the therapeutic effect of birabresib on solid or hematological malignancies were terminated prematurely because of limited efficacy (NCT02698176, NCT02698189, NCT02698176, NCT02296476). Therefore, combining BET inhibitors with other traditional drugs may open new possibilities for the development of antitumor strategies.

Numerous novel BET inhibitors have been identified recently, enhancing the landscape of therapeutic options. These include OPN-51107, a pan-BET inhibitor that mitigates T cell dysfunction in chronic lymphocytic leukemia;^489^ XL-126, a BD1-selective inhibitor noted for its potent anti-inflammatory effects;^490^ and DW-71177, another BD1-selective inhibitor geared towards AML treatment.^491^ Additional developments involve brain-permeable BD1-selective inhibitors for multiple sclerosis treatment,^492^ compounds with dual HDAC/BET inhibitory action for challenging tumors,^493^ phenoxyaryl pyridone derivatives as BD2-selective inhibitors for AML,^494^ and SRX3177, a potent triple-action CDK4/6-phosphoinositide 3-kinase-BET inhibitor for respiratory diseases linked to β-coronavirus.^495^ These advancements significantly contribute to understanding BET-targeted drug development, designing small molecule inhibitors tailored to the diverse pathological characteristics of human diseases.

##### YEATS domain inhibitors

Identified in 2014, the YEATS domain-comprising YAF9, ENL, AF9, TAF14, and SAS5—serves as a novel reader for histone acetylation. This domain also recognizes histone crotonylation and benzoylation, which are critical in regulating gene expression.^496–498^ The human genome encodes four YEATS domain-containing proteins: ENL, YEATS domain-containing 2 (YEATS2), AF9, and glioma amplified sequence 41 (GAS41). These proteins are primarily implicated in the pathogenesis of tumors, particularly hematologic malignancies, and represent promising targets for epigenetic therapies.^499–502^ Research has shown that the YEATS domain binds to acylated lysine side chains through a common binding pocket and engages in π-π-π stacking interactions, providing a structural and theoretical foundation for developing targeted inhibitors.^503^ A significant milestone was the identification of the first small-molecule chemical probe, SGC-iMLLT, which targets ENL and its paralog AF9. This probe’s inhibitory effects were validated in biological assays.^504^ Furthermore, another approach involves blocking the protein-protein interaction (PPI) between YEATS domain proteins and disruptor of telomeric silencing 1-like (DOT1L), effectively suppressing the activity of YEATS domain proteins.^505,506^ Current research is focused on developing selective inhibitors for various YEATS domain proteins, with the deepest insights into ENL inhibitors. In 2022, Liu et al.^507^ highlighted the promising potential of the oral ENL inhibitor TDI-11055 in treating AML in mouse models, advancing the clinical application of ENL inhibitors for AML treatment. Additionally, combination therapies involving ENL inhibitors with KAT or BET inhibitors have been emphasized.^508,509^ In 2020, Jiang et al.^510^ introduced the first selective inhibitor targeting the AF9 YEATS domain, presenting a novel cyclopeptide for in-depth exploration of the functional similarities and differences between AF9 and ENL, thereby laying the groundwork for novel YEATS domain inhibitor development. An optimized method for the solid-phase synthesis of these inhibitory cyclopeptides has since been proposed, significantly reducing preparation time and enhancing yield.^510^ Moreover, the study of amide-π interactions between histone acyl-lysine and the AF9 YEATS domain has led to the development of chemical compounds that disrupt this noncovalent interaction, notably those incorporating urea or aromatic rings.^511,512^ In 2021, the first selective GAS41 inhibitor was reported; this synthesized molecule binds to dimerized GAS41 YEATS domains and blocks interaction with acetylated histone H3 in cancer cell lines.^513^

##### PHD finger domain inhibitors

BD and PHD finger-containing protein (BRPF) and BD and PHD finger transcription factor (BPTF) are crucial targets involved in tumor progression and the development of resistance to molecularly targeted therapy drugs, such as kinase inhibitors and poly ADP-ribose polymerase (PARP) inhibitors.^514–516^ To date, an array of BRPF inhibitors featuring distinctive scaffolds—such as 3-acetyl-indole, 1,3-dimethylquinolin-2-one, 1,3-dimethyl benzimidazole, 1-(indolin-1-yl)ethan-1-one, 1,3-dimethylquinolin-2-one, and 2,3-dioxo-quinoxaline—has been identified. These compounds represent novel avenues for therapeutic innovation.^517–522^ However, as the inhibitory effects of these agents have primarily been confirmed in vitro, extensive efforts are required to advance these drugs to clinical trials. BPTF inhibitor development has not kept pace with those targeting other proteins with BD motifs, primarily remaining within fragment-based drug discovery. Only a handful have been tested in vivo or in vitro to demonstrate their inhibitory actions and therapeutic potential. AU1, the first small molecule selective for BPTF, has shown effectiveness in mouse models of gastric cancer and neuroblastoma.^516,523,524^ Bromosporine has exhibited significant antitumor effects in breast cancer and melanoma, suggesting promising therapeutic strategies for solid tumors.^525,526^ The novel selective inhibitor C620-0696 has shown cytotoxic effects in non-small-cell lung cancer cells overexpressing BPTF.^527^ The continued exploration of these inhibitors in oncology is highly anticipated.

### Epigenetics-targeted drugs and histone methylation

Histone methylation is a highly dynamic regulator crucial for activating or suppressing gene transcription. Histone methyltransferases, demethylases, and reader proteins modify and maintain epigenetic signals that influence chromatin structure and cellular functions. Their dysregulation is linked to a variety of diseases, particularly malignant tumors. Recent advances in biochemistry and understanding of pathogenesis have led to identifying and developing small-molecule inhibitors that target aberrant demethylation patterns (Table 5).

**Table 5:** Summary of histone methylation-targeted drugs for different diseases in clinical trials

Type	Drug	Target(s)	Condition(s)	Status/outcome(s)	Phase(s)	Other intervention(s)/drug(s)	Study ID/reference(s)
KMT inhibitor	CPI-1205	EZH2	B cell lymphoma	Completed (unpublished)	Phase I	—	NCT02395601
	CPI-1205	EZH2	Melanoma, NSCLC, RCC, urothelial carcinoma	Completed (unpublished)	Phase I	In combination with Ipilimumab	NCT03525795
	CPI-1205	EZH2	Castration-resistant prostate cancer	Unknown	Phase I/II	In combination with Enzalutamide or Abiraterone/Prednisone	NCT03480646
	CPI-0209	EZH2/EZH1	Ovarian cancer	Recruiting	Phase I	In combination with Carboplatin	NCT05942300
	CPI-0209	EZH2/EZH1	MF/Sezary syndrome	Recruiting	Phase I	—	NCT05944562
	CPI-0209	EZH2/EZH1	Urothelial carcinoma, ovarian cancer, endometrial carcinoma, DLBCL, PTCL, mesothelioma	Recruiting	Phase I/II	—	NCT04104776
	SHR2554	EZH2	Healthy volunteers	Completed (metabolizing enzymes in vivo regulates the plasma concentration of SHR2554)	Phase I	In combination with Itraconazole	NCT04627129^542^
	SHR2554	EZH2	Healthy volunteers	Unknown	Phase I	—	NCT05049083
	SHR2554	EZH2	Healthy volunteers	Completed (unpublished)	Phase I	—	NCT06010680
	SHR2554	EZH2	Healthy volunteers	Completed (unpublished)	Phase I	In combination with Fluconazole	NCT05661591
	SHR2554	EZH2	Healthy volunteers	Completed (unpublished)	Phase I	In combination with Omeprazole	NCT06093945
	SHR2554	EZH2	Healthy volunteers	Completed (drug exposures are essentially the same in fasted and fed states)	Phase I	Following a high-fat diet or fasting status	NCT04335266
	SHR2554	EZH2	Healthy volunteers	Completed (metabolizing enzymes in vivo regulates the plasma concentration of SHR2554)	Phase I	In combination with Rifampin	NCT04577885
	SHR2554	EZH2	Mature lymphoid neoplasms	Unknown (exhibits satisfied efficacy and acceptable adverse effects according to available data)	Phase I	—	NCT03603951^540,541^
	SHR2554	EZH2	FL	Not yet recruiting	Phase II	—	NCT06368167
	SHR2554	EZH2	HR-positive, HER2-negative, endocrine-resistant advanced BC	Recruiting	Phase II	Umbrella study	NCT04355858
	SHR2554	EZH2	PTCL	Recruiting	Phase I/II	Umbrella study	NCT05559008
	SHR2554	EZH2	TNBC	Recruiting	Phase I/II	Umbrella study	NCT03805399
	SHR2554	EZH2	B cell lymphoma, solid tumors	Recruiting	Phase I/II	SHR1701 (active comparator/followed by SHR2554)	NCT04407741
	SHR2554	EZH2	HL	Recruiting	Phase I/II	SHR1701 (active comparator/followed by SHR2554)	NCT05896046
	SHR2554	EZH2	PTCL	Recruiting	Phase I/II	In combination with CHOP	NCT06173999
	SHR2554	EZH2	Castration-resistant prostate cancer	Completed (unpublished)	Phase I/II	With or without SHR3680	NCT03741712
	SHR2554	EZH2	PTCL	Recruiting	Phase III	Chidamide (active comparator)	NCT06122389
	PF-06821497	EZH2	Castration-resistant prostate cancer, SCLC, FL	Recruiting	Phase I	—	NCT03460977
	GSK126	EZH2	DLBCL, FL, MM, solid tumors	Terminated (the maximal dose and schedule shows insufficient evidence of clinical activity)	Phase I	—	NCT02082977^546^
	XNW5004	EZH2	Squamous cell carcinoma of head and neck, urothelial carcinoma, prostate cancer, SCLC, NSCLC, cervical cancer	Recruiting	Phase I/II	In combination with Pembrolizumab	NCT06022757
	AXT-1003	EZH2	NHL	Recruiting	Phase I	—	NCT05965505
	EPZ-5676	DOT1L	AML, ALL	Completed (unpublished)	Phase I	—	NCT02141828
	EPZ-5676	DOT1L	AML, ALL, MDS, myeloproliferative disorders	Completed (exhibits good safety profiles while unsatisfied efficacy)	Phase I	—	NCT01684150^1146^
	EPZ-5676	DOT1L	AML with an 11q23 translocation or partial tandem duplication	Completed (large-scale trials should be hold)	Phase I/II	In combination with Azacitidine	NCT03701295
	EPZ-5676	DOT1L	ALL	Terminated (due to the study agent is no longer available)	Phase I/II	In combination with Cytarabine and Daunorubicin	NCT03724084
	EZM0414	SETD2	MM, DLBCL	Recruiting	Phase I	—	NCT05121103
	KTX-1001	NSD2	MM	Recruiting	Phase I	—	NCT05651932
PRMT inhibitor	GSK3368715	PRMT1	DLBCL, PDAC, bladder cancer, NSCLC	Terminated (due to a lack in observed clinical efficacy and the unfavorable risk/benefit analysis)	Phase I	—	NCT03666988^561^
	CTS-2190	PRMT1	PDAC, NSCLC, TNBC	Recruiting	Phase I/II	—	NCT06224387
	GSK3326595	PRMT5	TNBC, TCC, GBM, NHL, ACC, HR-positive BC, HPV-positive solid tumors, NSCLC	Completed (unpublished)	Phase I	With or without Pembrolizumab	NCT02783300
	GSK3326595	PRMT5	BC	Completed (unpublished)	Phase II	Blank-controlled	NCT04676516
	GSK3326595	PRMT5	MDS, AML	Terminated (due to an internal review of clinical data)	Phase I/II	With or without Azacitidine	NCT03614728
	JNJ64619178	PRMT5	NHL, MDS, solid tumors	Active, not recruiting (clinical benefit is limited)	Phase I	—	NCT03573310^568^
	JNJ64619178	PRMT5	Solid tumors	Completed (exhibits manageable dose-dependent toxicity with limited clinical benefit)	Phase I	—	^1147^
	PF06939999	PRMT5	NSCLC, urothelial carcinoma, squamous cell carcinoma of head and neck	Terminated (exhibits tolerable safety profiles and objective clinical responses in a subset of patients)	Phase I	With or without Docetaxel	NCT03854227^569,1148^
	TNG908	PRMT5	NSCLC, mesothelioma, PDAC, sarcoma, GBM	Recruiting	Phase I/II	—	NCT05275478
	MRTX1719	PRMT5	Mesothelioma, PDAC, NSCLC, malignant peripheral nerve sheath tumor	Recruiting	Phase I/II	—	NCT05245500^571^
	PRT543	PRMT5	DLBCL, myelodysplasia, myelofibrosis, ACC, MCL, AML, CMML	Completed (exhibits limited efficacy in ACC)	Phase I	—	NCT03886831^572^
	PRT811	PRMT5	Solid tumors, CNS lymphoma, gliomas	Completed (unpublished)	Phase I	—	NCT04089449
	SKL27969	PRMT5	Solid tumors	Terminated (due to portfolio prioritization)	Phase I/II	—	NCT05388435
	AMG193	PRMT5	Biliary tract cancer, PDAC	Recruiting	Phase I	In combination with Gemcitabine/Cisplatin/Pembrolizumab, or Gemcitabine/Nab-paclitaxel, or modified FOLFIRINOX	NCT06360354
	AMG193	PRMT5	NSCLC	Recruiting	Phase I	With or withought Carboplatin/Paclitaxel/Pembrolizumab, or Carboplatin/Pembrolizumab/Pemetrexed, or Pembrolizumab, or Sotorasib	NCT06333951
	AMG193	PRMT5	MTAP-null solid tumors	Recruiting	Phase I/II	With or without Docetaxel	NCT05094336
	AMG193	PRMT5	MTAP-null solid tumors	Recruiting	Phase I/II	In combination with IDE397	NCT05975073
	SH3765	PRMT5	Advanced malignant tumors	Not yet recruiting	Phase I	—	NCT05015309
	TNG462	PRMT5	MTAP-null solid tumors	Recruiting	Phase I/II	—	NCT05732831
	SCR6920	PRMT5	Solid tumors, NHL	Recruiting	Phase I	—	NCT05528055
	SYHX-2001	PRMT5	Solid tumors	Recruiting	Phase I	—	NCT05407909
KDM inhibitor	Tranylcypromine	LSD1	Non-APL AML subtypes, MDS	Completed (unpublished)	Phase I	In combination with ATRA	NCT02273102
	Tranylcypromine	LSD1	Non-APL AML subtypes	Unknown	Phase I/II	In combination with ATRA	NCT02261779
	Tranylcypromine	LSD1	Non-APL AML subtypes	Unknown	Phase I/II	In combination with ATRA and Cytarabine	NCT02717884
	ORY-1001	LSD1	AML, MDS	Recruiting	Phase I	In combination with Azacitidine and Venetoclax	NCT06357182
	ORY-1001	LSD1	AML	Recruiting	Phase I	In combination with Gilteritinib	NCT05546580
	ORY-1001	LSD1	SCLC	Not yet recruiting	Phase I/II	Atezolizumab and Durvalumab (active comparator/followed by ORY-1001)	NCT06287775
	ORY-1001	LSD1	AML	Completed (exhibits a good safety profile without significant extra-hematologic toxicity)	Phase I	—	EudraCT 2013-002447-29
	ORY-1001	LSD1	AML	Completed (unpublished)	Phase II	In combination with Azacitidine	EudraCT 2018-000482-36^624^
	ORY-2001	LSD1	Healthy volunteers	Completed (exhibits good safety and tolerability)	Phase I	Placebo-controlled	EUDRACT 2015-003721-33^627^
	ORY-2001	LSD1	MS	Ongoing (exhibits safety and tolerability according to early clinical data)	Phase II	Placebo-controlled	EudraCT 2017-002838-23
	ORY-2001	LSD1	AD	Completed (exhibits good efficacy and tolerability)	Phase II	Placebo-controlled	EudraCT 2017-004893-32
	ORY-2001	LSD1	ADHD, BPD, ASD	Completed (exhibits good efficacy and tolerability)	Phase II	—	EudraCT 2018-002140-88
	ORY-2001	LSD1	AD	Completed (exhibits good efficacy and tolerability)	Phase II	Placebo-controlled	EudraCT 2019-001436-54
	ORY-2001	LSD1	ARDS	Completed (exhibits good efficacy and tolerability)	Phase II	In combination with standard care treatment	EudraCT 2020-001618-39
	ORY-2001	LSD1	AD	Completed (unpublished)	Phase II	Placebo-controlled	NCT03867253
	ORY-2001	LSD1	BPD	Completed (unpublished)	Phase II	Placebo-controlled	NCT04932291
	GSK-2879552	LSD1	SCLC	Terminated (due to the unfavorable risk/benefit analysis)	Phase I	—	NCT02034123
	GSK-2879552	LSD1	AML	Terminated (due to the unfavorable risk/benefit analysis)	Phase I	In combination with ATRA	NCT02177812
	GSK-2879552	LSD1	MDS	Terminated (due to the unfavorable risk/benefit analysis)	Phase I/II	With or without Azacitidine	NCT02929498
	IMG-7289	LSD1	AML	Recruiting	Phase I	In combination with Venetoclax	NCT05597306
	IMG-7289	LSD1	AML, MDS	Completed (exhibits a good safe profile)	Phase I/II	With or without ATRA	NCT02842827
	IMG-7289	LSD1	SCLC	Active, not recruiting	Phase I/II	In combination with Atezolizumab	NCT05191797
	INCB059872	LSD1	Ewing sarcoma	Terminated (due to business decision)	Phase I	—	NCT03514407
	INCB059872	LSD1	AML, MDS, SCLC, myelofibrosis, Ewing sarcoma, poorly differentiated neuroendocrine tumors	Terminated (due to business decision)	Phase I/II	With or without ARTA, Azacitidine, and Nivolumab	NCT02712905
	INCB059872	LSD1	NSCLC, colorectal cancer	Terminated (due to sponsors’ decision)	Phase I/II	In combination with Pembrolizumab and Epacadostat	NCT02959437
	SP-2577	LSD1	Solid tumors	Completed (unpublished)	Phase I	—	NCT03895684
	SP-2577	LSD1	Ewing sarcoma, myxoid liposarcoma, desmoplastic small round cell tumor	Active, not recruiting	Phase I	With or without Cyclophosphamide and Topotecan	NCT03600649
	SP-2577	LSD1	Ovarian cancer, endometrial cancer	Withdrawn (due to salaries discontinued support)	Phase I	In combination with Pembrolizumab	NCT04611139
	SP-2577	LSD1	CMML, MDS	Recruiting	Phase I/II	In combination with Azacytidine	NCT04734990
	SP-2577	LSD1	Ewing sarcoma, myxoid liposarcoma, desmoplastic small round cell tumor	Enrolling by invitation	Phase I/II	—	NCT05266196
	CC-90011	LSD1	AML	Terminated (due to business decision)	Phase I	Azacitidine and Venetoclax (active comparator/followed by CC-90011)	NCT04748848
	CC-90011	LSD1	Castration-resistant prostate cancer	Completed (unpublished)	Phase I	In combination with Abiraterone and Prednisone	NCT04628988
	CC-90011	LSD1	NHL, solid tumors	Terminated (due to business decision)	Phase I	In combination with Rifampicin	NCT02875223^1149,1150^
	CC-90011	LSD1	SCLC	Active, not recruiting	Phase I	In combination with Cisplatin and Etoposide	NCT03850067
	CC-90011	LSD1	SCLC, NSCLC	Completed (unpublished)	Phase II	In combination with Nivolumab	NCT04350463
	4SC-202	LSD1	AML, ALL, MDS, CLL, MM	Completed (exhibits a good safety profile and antitumor activities)	Phase I	—	NCT01344707^1102^
	4SC-202	LSD1	Oesophagogastric adenocarcinoma, colorectal cancer	Unknown (oesophagogastric adenocarcinoma cohort meets the criteria to expand to stage 2 according to disclosed data)	Phase II	In combination with Avelumab	NCT03812796^1103^
	4SC-202	LSD1	Melanoma	Completed (unpublished)	Phase I/II	In combination with Pembrolizumab	NCT03278665
	4SC-202	LSD1	Melanoma	Active, not recruiting (4SC-202 addition does not increase treatment efficacy according to early clinical data)	Phase I/II	Nivolumab (active comparator/in combination with 4SC-202); in combination with Nivolumab/Ipilimumab	NCT04133948^424^
	JBI-802	LSD1, HDAC6	SCLC and other neuroendocrine-derived cancers	Recruiting	Phase I/II	—	NCT05268666
	TAK-418	LSD1	Healthy volunteers	Completed (exhibits good tolerability, pharmacokinetic and pharmacodynamic effects)	Phase I	Placebo-controlled	NCT03228433^638^
	TAK-418	LSD1	Healthy volunteers	Terminated (due to business decision)	Phase I	Placebo-controlled	NCT03501069^638^
	TAK-418	LSD1	Healthy volunteers	Terminated (due to administrative reasons)	Phase I	In combination with [18 F]MNI-1054 (radiotracer)	NCT04202497
	LH-1802	LSD1	AML, MDS	Ongoing	Phase I	—	CTR20222026
	SYHA1807	LSD1	SCLC	Unknown	Phase I	—	NCT04404543
WDR domain inhibitor	MAK683	EED	DLBCL	Active, not recruiting	Phase I	—	NCT02900651

*ACC* adenoid cystic carcinoma, *AD* Alzheimer’s disease, *ADHD* attention deficit hyperactivity disorder, *ALL* acute lymphoblastic leukemia, *AML* acute myeloid leukemia, *APL* acute promyelocytic leukemia, *ARDS* acute respiratory distress syndrome, *ASD* autism spectrum disorder, *ATRA* all-trans-retinoicacid, *BC* breast cancer, *BPD* borderline personality disorder, *CHOP* Cyclophosphamide, Hydroxydoxorubicin, Oncovin, and Prednisone, *CLL* chronic lymphocytic leukemia, *CMML* chronic myelomonocytic leukemia, *CNS* central nervous system, *DLBCL* diffuse large B cell lymphoma, *DOT1L* disruptor of telomeric silencing 1-like, *EED* embryonic ectoderm development, *EZH2* enhancer of zeste homolog 2, *FL* follicular lymphoma, *GBM* glioblastoma multiforme, *HDAC* histone deacetylase, *HER2* human epidermal growth factor receptor 2, *HL* Hodgkin lymphoma, *HPV* human papillomavirus, *HR* hormone receptor, *KDM* lysine demethylase, *KMT* lysine methyltransferase, *LSD1* lysine specific demethylase 1, *MCL* mantle cell lymphoma, *MDS* myelodysplastic syndrome, *MF* mycosis fungoides, *MM* multiple myeloma, *MS* multiple sclerosis, *MTAP* methyl-thioadenosine phosphorylase, *NHL* non-Hodgkin lymphoma, *NSD* nuclear receptor binding SET domain protein, *NSCLC* non-small cell lung cancer, *PDAC* pancreatic ductal adenocarcinoma, *PRMT* protein arginine methyltransferase, *PTCL* peripheral T cell lymphoma, *RCC* renal cell carcinoma, *SCLC* small cell lung cancer, *SETD2* SET domain-containing histone lysine methyltransferase 2, *TCC* transitional cell carcinoma of the urinary system, *TNBC* triple-negative breast cancer, *WDR* WD40 repeat

#### Targeting the writer of histone methylation: KMT and PRMT

Histone methyltransferases (HMTs), including KMTs and protein arginine methyltransferases (PRMTs), are central to regulating histone methylation and are implicated in numerous biological and pathological processes. Inhibitors of HMTs are extensively researched as potential therapeutic agents. Notably, innovative drug discovery strategies for HMT proteins—such as covalent inhibition independent of SAM-competitive or substrate-competitive mechanisms, dual-target inhibition, and targeted degradation strategies—have received considerable attention and have rapidly progressed.^528–530^ These inhibitors, in addition to marketed drugs, are being advanced to clinical practice for further evaluation and oversight.

##### EZH2 inhibitors

Since the identification of the suppressor of variegation 3-9 homolog 1 (SUV39H1), the inaugural histone KMT8 discovered in 2000, numerous proteins mediating histone methylation have been reported. These include EZH1/2, euchromatic histone-lysine N-methyltransferase 2 (G9a/EHMT2), G9a-like protein (GLP/EHMT1), DOT1L, and various SET domain-containing histone lysine methyltransferase (SETD) and nuclear receptor binding SET domain protein (NSD) families.^531–533^ Over recent decades, considerable efforts have focused on developing efficient and selective inhibitors targeting various histone KMT subfamilies with potential therapeutic applications in disease treatment.^534–537^

In addition to the two marketed drugs summarized in the previous section, tazemetostat (EPZ-6438) and valemetostat (DS-3201b), numerous novel EZH2 inhibitors are under investigation, with several advances in clinical studies, particularly compounds featuring the 2-pyridone moiety which encompass both bicyclic heteroaromatic and monocyclic aromatic rings.^538^ CPI-1205 (lirametostat) has undergone evaluation in three clinical trials (NCT03480646, NCT03525795, NCT02395601) to assess its tolerance and therapeutic potential. Although CPI-1205 has shown good tolerability in phase I stages, phase II trials have yet to provide sufficient data to confirm its antitumor efficacy. CPI-0209 (tulmimetostat), an oral, next-generation dual EZH2/EZH1 inhibitor developed by the same company, is currently under clinical trial for treating both solid tumors and hematological malignancies (NCT05944562, NCT05942300, NCT04104776). SHR2554, a highly selective EZH2 inhibitor, has demonstrated potent efficacy both in vitro and in vivo.^539^ Its first-in-human, dose-escalation, and dose-expansion phase 1 trial conducted at 13 hospitals in China in 2018 indicated good tolerance and promising antitumor activity in patients with R/R lymphomas.^540,541^ A pharmacokinetic study revealed that combining itraconazole, an inhibitor of CYP3A4-metabolizing enzymes, with SHR2554 improves its plasma concentration while maintaining a favorable safety profile, suggesting a new therapeutic strategy.^542^ PF-06821497, a lactam-derived EZH2 inhibitor, was optimized from a series of similar compounds using ligand-based and physicochemical-property-based strategies, showing optimal inhibitory and therapeutic effects in mouse models.^543^ It also has demonstrated synergistic effects in combination with HDAC inhibitors, inhibiting proliferation and inducing apoptosis in cancer cell lines.^544^ Currently, two clinical studies are exploring appropriate administration methods for PF-06821497, such as intravenous injection or oral intake, and whether it can be consumed with food (NCT06392230, NCT05767905). In addition to demonstrating a strong therapeutic effect in tumor treatment in animal models, GSK126 has also achieved significant breakthroughs in enhancing β-like cell regeneration among patients with T1DM.^111,545^ However, a terminated phase I clinical trial revealed insufficient evidence of clinical activity for GSK126 at tolerable doses.^546^ XNW5004 (NCT06022757) and AXT-1003 (NCT05965505) are innovative EZH2 inhibitors currently in clinical trials, reflecting ongoing advancements in this therapeutic area. Moreover, astemizole, originally an antiallergy medication inhibiting histamine receptor H1, has recently been shown to disrupt the EZH2-embryonic ectoderm development (EED) PPI within the PRC2, offering new perspectives in developing EZH2/PRC2 inhibitors.^547^

##### DOT1L inhibitors

EPZ-5676 (pinometostat), EPZ004777, and SGC0946 are three selective inhibitors of DOT1L that are currently under extensive research. EPZ-004777 was the first SAM-competitive inhibitor of DOT1L to demonstrate in vivo efficacy.^548^ Despite showing promising therapeutic effects in various subtypes of AML through cell experiments and animal models, EPZ-004777’s preclinical application has been largely constrained by its pharmacokinetic characteristics.^549,550^ EPZ-5676 has been developed to improve selectivity and inhibition effects, showing potential as a therapeutic agent for mixed lineage leukemia (MLL).^551^ Early investigations using patient-derived xenografts and mouse models have indicated that EPZ-5676 exhibits potent antileukemic activities, facilitating further evaluation.^552,553^ In three completed clinical trials (NCT01684150, NCT02141828, NCT03701295), EPZ-5676 has been assessed for safety, tolerability, and preliminary antitumor activity in pediatric patients with MLL, with the combination of EPZ-5676 and azacytidine in a phase Ib/II study expected to show synergistic antiproliferative activities (NCT03701295). SGC0946, a brominated analog, serves as another selective inhibitor of DOT1L. Its therapeutic potential, either as monotherapy or in combination with other inhibitors such as HDACs and the mitogen-activated protein kinase pathway, has been observed in various solid tumors, setting the groundwork for clinical trials of SGC0946.^554–556^

Beyond EZH2 and DOT1L, several inhibitors targeting other subfamilies are being investigated in clinical studies, including EZM0414 and KTX-1001. EZM0414, a novel inhibitor of SETD2 derived from the optimization of EPZ-719, exhibits improved pharmacokinetic properties and potent pharmacodynamic activity in mouse xenograft models.^557^ A phase I/Ib clinical trial is currently underway to explore the safety, tolerability, and therapeutic efficacy of EZM0414 in patients with R/R MM and R/R diffuse large B-cell lymphoma (NCT05121103). KTX-1001, a selective NSD2 inhibitor, has been FDA-approved for clinical trials since 2022 and is being studied in a phase I trial to treat patients with R/R MM (NCT05651932). These meticulously organized clinical trials focusing on KMTs are drawing increasing attention, leading to significant breakthroughs in understanding the relationship between human diseases and aberrant histone methylation.

##### PRMT inhibitors

Significant progress has been made in developing inhibitors for type I PRMTs (PRMT1-4, 6, and 8) and a selective inhibitor targeting PRMT5, with several agents entering the early phases of clinical trials.

Two type I PRMTs inhibitors are already in clinical stages, including GSK3368715 and CTS-2190. GSK3368715 (EPZ019997), an oral, reversible inhibitor of PRMT1/6/8 developed for treating tumors and pulmonary disorders.^558–560^ GSK3368715 underwent a phase 1 clinical trial for treating solid tumors and diffuse large B-cell lymphoma in 2018. However, the first clinical application of a PRMT1 inhibitor did not meet expectations and was terminated early in 2022 due to its ineffectivenes.^561^ Given the adverse events potentially caused by high and sustained concentrations of the inhibitor in vivo, research into PROTAC-based degraders of GSK3368715 has intensified, potentially offering therapeutic benefits at lower doses and reducing adverse effects.^562^ CTS-2190, another inhibitor targeting PRMT1/3/4/6, received clinical trial approvals from the US FDA and China NMPA in February and April 2023, respectively. A phase I/II clinical trial is being conducted to evaluate its tolerability and preliminary antitumor activity in healthy participants and patients with solid tumors (NCT06224387).

Thirteen PRMT5 inhibitors have advanced to phase I and II clinical trials. Among these, GSK3326595, JNJ64619178, and PF06939999 were the earliest selective PRMT5 inhibitors to receive clinical trial approvals. GSK3326595 is a substrate-competitive inhibitor, while JNJ64619178 and PF-06939999 function as SAM-competitive agents.^563–565^ The efficacy and understanding of GSK3326595 primarily rely on animal model data due to a lack of published results from completed clinical trials. This inhibitor has been shown to induce DNA damage in cancer cells and enhance the antiproliferative effects of poly ADP-ribose polymerase inhibitors, such as niraparib;^566^ however, long-term or chronic use of GSK3326595 is associated with potential liver-related adverse effects.^567^ A completed phase I clinical trial of JNJ-64619178 determined that a daily dose of 0.5 mg was better tolerated by participants with R/R B cell non-Hodgkin lymphoma, though it demonstrated limited therapeutic effects.^568^ Conversely, PF-06939999 has shown an acceptable safety profile and clinical efficacy in its phase I trial.^569^ TNG908 and MRTX1719, both brain-penetrant PRMT5 inhibitors, have shown promise in selectively targeting cancer cells deficient in methylthioadenosine phosphorylase in both preclinical models and clinical trials.^570,571^ Phase I/II clinical trials for these drugs recruit participants to assess their therapeutic effects on various solid tumors (NCT05245500). Prelude Therapeutics has developed PRT543 and PRT811, leading oral PRMT5 inhibitors whose safety profiles and preliminary therapeutic potential have been evaluated in phase I clinical trials (NCT04089449, NCT03886831). PRT543 has demonstrated good tolerance and efficacy among patients with adenoid cystic carcinoma, warranting further advanced clinical testing.^572^ A phase I/II clinical trial of SKL27969 began in 2022 to evaluate its safety, tolerability, pharmacokinetics, pharmacodynamics, and preliminary efficacy in patients with advanced solid tumors. However, this study was terminated in 2024 due to portfolio prioritization, with no significant safety trends or issues identified during its execution (NCT05388435). Other PRMT5 inhibitors, such as AMG193, SH3765, TNG462, SCR6920, and SYHX-2001, are currently under investigation in clinical trials and are in the “recruiting” phase. Given that most of the small molecules or core scaffolds of PRMT5 inhibitors have been examined only in cellular experiments, there remains a significant gap in knowledge regarding their efficacy and therapeutic effects in vivo. Therefore, it is imperative to bridge the crucial divide between fundamental research and clinical application.

#### Targeting the eraser of histone methylation: KDM

KDMs are enzymes that remove histone and nonhistone methylation. They can be divided into two categories based on their molecular structures: flavin adenine dinucleotide-dependent KDM (KDM1) and Fe(II)- and α-KG-dependent KDM (KDM2-7), also called Jumonji C (JmjC)-KDMs.^573^ Both upregulation and downregulation of KDMs can affect the expression of pathological genes in cancers or other disorders. Currently, representative inhibitors of diverse KDM proteins are being investigated. Based on catalytic mechanisms, lysine specific demethylase 1 (LSD1/KDM1A) inhibitors can be divided into irreversible and reversible inhibitors; KDM2-7 inhibitors are classified into four types: α-KG cofactor mimics or inhibitors of α-KG oxygenases (such as N-oxalylglycine), metal cofactor disruptors, histone substrate competitive inhibitors, and other substrate- and cofactor-independent inhibitors.^574^

##### KDM2/7 inhibitors

KDM2 and KDM7 proteins, which belong to the JmjC-KDM subfamilies, share high similarity in their Fe(II)- and α-KG-binding residues.^575^ The development of inhibitors for KDM7 and KDM2 has typically co-occurred. In 2013, a series of hydroxamate analogs featuring an alkyl chain were identified. These compounds demonstrated antiproliferative activity in cancer cells by inhibiting KDM2A, KDM7A, and KDM7B.^576^ Similarly, Gerken et al.^577^ developed a series of novel KDM2A/7A inhibitors characterized by saturated indoline ring systems. These indoline-containing compounds exhibited potent and selective effects on KDM2A/7A at low micromolar concentrations, with notable cellular activity. Nonetheless, addressing limitations such as cytotoxicity and off-target effects remains challenging for future research. Other selective inhibitors have also been identified, including a cyclic peptide inhibitor, OC9, designed to target the PHD finger domain of KDM7. This inhibitor results in the inhibition of KDM7B and the activation of KDM7A.^578^ Through virtual screening of α-KG oxygenases, daminozide, a plant growth regulator, was found to selectively inhibit KDM2A. The therapeutic potential of daminozide was observed in mouse models of osteoarthritis, pointing to new directions for developing 2KG-competed inhibitors with enhanced selectivity, although its use in humans is unlikely.^579,580^

##### KDM3 inhibitors

In addition to IOX1, various inhibitors of the KDM3 family have been identified, most exhibiting a pan-inhibitory effect across all family members. Through virtual screening of natural products and traditional Chinese medicine components, compounds JDI-4, JDI-12, and JDI-16 selectively bind to the JmjC domains of KDM3B and KDM3C.^581^ Subsequent in vitro and in vivo studies confirmed the inhibitory effect and antitumor potential of JDI-16 in a KDM3-dependent manner.^581^ Another compound, JDM-7, also identified from this screening, inhibits KDM3A and KDM3B in AML cell lines, although initial observations indicated limited effects on the KDM3 family.^582^ Additionally, through high-throughput screening of benzhydryl amine derivatives, CBA-1 was found to be a potent inhibitor of KDM3A, exhibiting antiproliferative effects on colorectal cancer cell lines.^583^ The use of CBA-1 in zebrafish models also showed minimal toxicity, suggesting its potential as a promising drug for clinical application.^583^

##### KDM4 inhibitors

Given the critical roles of KDM4s in cancers and the inherent complexity of the KDM4 subfamily, significant efforts have been dedicated to developing KDM4 inhibitors. These inhibitors are categorized into four previously reviewed classes: α-KG cofactor mimics, Metal cofactor disruptors, histone substrate competitive inhibitors, and inhibitors targeted reader domains, having been summarized extensively in previous work.^584,585^ In addition to these established categories, we emphasize the progress in novel inhibitors that have not yet been summarized, further expanding the scope of therapeutic options against KDM4-related cancers.

TACH101 is a novel pan-inhibitor of KDM4A-D, competitively inhibiting α-KG without affecting other KDM subfamilies. The therapeutic effects of TACH101 have been demonstrated in organoids and xenograft models, suggesting its potential as an anticancer agent worthy of further investigation in animal studies.^586^ SD49-7, a derivative of SD70, is another novel KDM4 inhibitor. It has shown a stronger effect than SD70 in suppressing the proliferation of AML cell lines and enhancing the progression of resistant tumors in mouse models.^587^ Based on virtual screening, 2-(methylcarbamoyl)isonicotinic acid has been identified as an initial active fragment specifically inhibiting KDM4A by preventing its binding to H3K9me3 in a substrate-competitive manner.^588^ Molecular docking and dynamics approaches have recently revealed that a series of natural products containing sugars, aromatic rings, and OH or O^−^ groups can interact with KDM4 and inhibit its activities.^589^ However, the mechanisms of these interactions remain unclear, underscoring the need for further development of these potential drugs.

##### KDM5 inhibitors

KDM5 inhibitors have shown significant therapeutic potential, though many compounds still lack sufficient evidence to confirm their efficacy and safety in vivo.

A prevailing approach in KDM5 inhibition involves designing small molecules that compete with α-KG for binding sites.^590^ Among these, KDOAM-25 is a potent and selective inhibitor affecting MM and triple-negative breast cancer cells, with minimal adverse effects observed in vivo applications. Nevertheless, its poor cell membrane permeability hinders its efficacy.^591^ RS3195 exhibits inhibitory effects on KDM5B and KDM5D in vitro. Due to potential toxicity, RS5033 was developed as an alternative, featuring a phenyl ring instead of a pyrrole nucleus to improve tolerance.^592^ KDM5-C49, an analog of 2,4-PDCA, binds to KDM5B in vitro and inhibits its enzymatic activities.^593^ To enhance cell membrane permeability and selectivity, derivatives KDM5-C48 and KDM5-C70 have been developed.^593–595^ Through high-throughput virtual screening, a series of cyclopenta[c]chromen derivatives targeting KDM5A have been identified as promising drugs due to their potent inhibitory effects and low toxicity.^596^ N70, a thienopyridine-based selective KDM5A inhibitor, displays α-KG-competitive inhibition, while its analog, N71, binds irreversibly to KDM5A through covalent modifications.^597^

Numerous compounds, identified through virtual screening and optimization of reported inhibitory molecules, employ different mechanisms of action.^598,599^ Among these, KDM5-inh1 and CPI-455 are broadly studied pan-inhibitors of KDM5. Using either KDM5-inh1 or CPI-455 has demonstrated therapeutic effects on cancer cell lines and has facilitated synergistic interactions with conventional antitumor agents.^600,601^ Further research should explore the potential for this synergy in animal models. GS-5801, designed from GS-080—one of the most potent KDM5 inhibitors—shows significant anti-HBV activity. Despite its promise, the in vivo effects of GS-5801 have not met expectations, underscoring the need for additional studies to enhance its efficacy.^602^ Utilizing the AlphaScreen method, ryuvidine was identified as an inhibitor of KMD5A/B/C, exhibiting substantial therapeutic impact on drug-tolerant cells.^603^ Dexmedetomidine, recently identified as a KDM5 inhibitor, is utilized to manage acute kidney injury in a KDM5-dependent manner.^604^ A novel approach was introduced by Yang et al.,^605^ who reported the first selective metal-based KDM5A inhibitor, rhodium(III) complex1. This compound disrupts the interaction between KDM5A and H3K4me2/3, offering a new scaffold for optimizing KDM5A-targeted drugs. The screening of imidazopyridine-analogs of zolpidem led to the discovery of O4I3, a novel chemical inhibitor of KDM5A. O4I3 generates and sustains patient-specific induced pluripotent stem cells in vitro.^606^ Additionally, TK-129, a pyrazole-based KDM5B inhibitor, is applied in treating cardiovascular diseases.^607^ High-throughput screening technology has facilitated the identification of PBIT, another novel KDM5B inhibitor. Despite its promising attributes, PBIT exhibits unstable therapeutic effects across different cell lines, necessitating careful consideration of its application in treatment.^608^ Similarly, several pyrazole derivatives that inhibit KDM5B have been recognized, with several demonstrating potent activity in cells, suggesting new therapeutic strategies.^609^ Furthermore, Iida et al.^610^ designed a selective KDM5C inhibitor with a triazole scaffold and subsequently synthesized a KDM5C degrader using PROTAC techniques. This selective degrader shows enhanced inhibitory effects on prostate cancer cell lines compared to its prodrug, thus expanding the possibilities for anticancer agent design.

##### KDM6 inhibitors

The KDM6 subfamily has gained attention as a therapeutic target for various diseases. GSK-J1 and GSK-J4 are two well-studied classical inhibitors of KDM6B, showing significant potential in treating autoimmune diseases, metabolic disorders, and tumors, and enhancing the effectiveness of traditional antitumor agents.^611–614^ Using optimized delivery systems for GSK-J1 has further advanced the development of effective in vivo strategies.^615^ Beyond these compounds, novel inhibitors have been introduced. For instance, KDOBA67, a hydroxyl derivative of GSK-J4, demonstrates favorable cell permeability in chordoma cell lines and inhibits the progression of chordoma.^616^ Employing a virtual fragment screening approach, Giordano et al.^617^ identified a series of benzoxazole scaffold compounds that bind to the KDM6B subfamily with high affinity, showing therapeutic promise in melanoma cell lines. Zhang et al.^618^ developed a simple capillary electrophoresis method for screening KDM6B inhibitors, leading to the identification of salvianic acid A and puerarin 6”-O-xyloside as effective agents. Additionally, Jones et al.^619^ used computational methods to develop an optimized peptide derived from the H3 C-terminus, which may enhance selectivity when linked with known inhibitors.

##### LSD1 inhibitors

Extensive research has been conducted on the biological and pathological functions of LSD1 and its inhibitors. Compared to other KDM subfamilies, LSD1 inhibitors have seen significant advances.^620^ Currently, several LSD1 inhibitors such as tranylcypromine (TCP), ORY‐1001 (ladademstat), ORY‐2001, GSK‐2879552, IMG-7289 (bomedemstat), INCB059872, SP‐2577 (seclidemstat), CC-90011 (pulrodemstat), 4SC‐202 (domatinostat), JBI‐802, TAK-418, LH‐1802, and SYHA1807, are undergoing clinical trials.

TCP, an irreversible inhibitor, is being used in clinical practice among patients with AML and MDS, showing promising effects either alone or in combination with all-trans-retinoic acid in phase I/II clinical trials, with overall response rates exceeding 20%.^621,622^ Building on TCP’s structure, novel inhibitors like ORY-1001, ORY-2001, GSK-2879552, INCB059872, and IMG-7289 have been developed, which also bind irreversibly to LSD1.^623^ These advancements have broadened the spectrum of treatable diseases with LSD1 inhibitors. Notably, ORY-1001 and ORY-2001, both orally administered, have been evaluated for their effectiveness in R/R hematologic malignancies and neurological disorders such as borderline personality disorder and AD.^624–628^ In completed phase I clinical trials, ORY-1001 exhibited a good safety profile without significant extrahematologic toxicity among healthy volunteers and patients with AML, indicating good therapeutic potential.^624,627^ GSK2879552 has shown antitumor efficacy in animal models,^629^ yet several clinical trials have been terminated due to a high incidence of adverse events.^630,631^ Similarly, clinical trials for INCB059872 were halted due to business decisions, among other reasons (NCT02959437, NCT03514407, NCT03132324, NCT02712905). Greater attention must be dedicated to evaluating the tolerability and efficacy of novel treatments. According to completed clinical trials, IMG-7289 demonstrates potential in ameliorating several blood disorders (NCT04254978, NCT03136185, NCT02842827), with numerous recent registrations for novel clinical trials concerning this drug. SP-2577 and CC-9001 are reversible LSD1 inhibitors,^632^ which, compared to their irreversible counterparts, exhibit enhanced safety profiles and have been extensively studied in both solid tumors and hematological malignancies.^633,634^ Domatinostat and JBI-802, dual inhibitors targeting LSD1 and HDAC, selectively interact with class I HDAC isoenzymes and HDAC6, respectively.^620^ Although promising antitumor effects have been observed in cancer cell lines, the therapeutic potential of domatinostat requires further exploration due to its unfavorable toxicity.^424,635^ TAK-418, a novel LSD1 inhibitor noted for its effective brain penetration, is considered a potential treatment for central nervous system disorders.^636,637^ The administration of TAK-418 was well tolerated by healthy volunteers in a phase I clinical trial, laying a solid foundation for further investigation.^638^ LH-1802 and SYHA1807, novel inhibitors, are currently under clinical trial evaluation for metastatic prostate cancer and small-cell lung cancer, respectively (NCT03678025, NCT04404543). The encouraging outcomes from these clinical-stage applications have spurred greater interest in the development of LSD1 inhibitors, with ongoing efforts to discover effective and tolerable agents.

#### Targeting the reader of histone methylation: reader domains

The identification of histone lysine and arginine methylation is attributed to proteins possessing malignant brain tumor (MBT) domains, chromodomains, Tudor domains, proline-tryptophan-tryptophan-proline (PWWP) domains, PHD fingers, and WD40 repeat (WDR) domains.^639^ Notably, enzymes that serve as writers or erasers for histone methylation may also contain these reader modules, such as PHD fingers and Tudor domains, aiding in recognizing residues they catalyze.^640^ Although numerous inhibitors targeting reader domains have been discovered, nearly half of these originate from structure-based virtual screenings and lack in vivo evaluation of their inhibitory effects and therapeutic activity.^641^ Encouragingly, MAK683, an inhibitor targeting EED—a representative histone methylation reader containing the WDR domain—has entered clinical trials.^642^ Currently, MAK683 is in a phase I clinical trial for treating diffuse large B-cell lymphoma (NCT02900651). Inspired by this milestone, many potent and selective inhibitors of EDD and other molecules targeting reader domain proteins are expected to advance into clinical trials as promising therapeutic strategies.

### Epigenetics-targeted drugs and m6A

RNA m6A methylation, a prevalent and conserved modification in eukaryotic RNAs, is crucial in determining transcript fate at the post-transcriptional level through RNA processing, export, degradation, and translation. Dysregulated m6A regulators contribute to various pathological conditions, particularly in the pathogenesis of diverse tumors.^643^ With the identification of various enzymes involved in m6A modification—including writers, erasers, and readers—the reversibility of m6A modification has been increasingly recognized, providing a foundation for developing epigenetics-targeted drugs that regulate RNA m6A as a core mechanism.

#### Targeting the writer of m6A: METTL3

METTL3 plays a critical role in the m6A modification process by transferring methyl groups from SAM to target RNA, catalyzing the conversion of adenosine to methyladenosine. This function of METTL3, the most extensively studied m6A writer, has been linked to the development of various pathologies, notably various tumors.^644^ Recent research has highlighted a range of inhibitors and agonists targeting METTL3, with several epigenetic drugs demonstrating promising efficacy both in vitro and in vivo, thus reinforcing the significance of METTL3 regulation in disease pathology and its potential as a therapeutic target.

##### METTL3 inhibitors

The study of METTL3 inhibitors has attracted increasing attention due to their diverse roles in regulating gene expression across different diseases. These inhibitors are categorized into competitive and allosteric inhibitors and gene expression suppressors, each leveraging distinct mechanisms of action.

SAM analog is the dominant part of the competitive inhibitors, initially developed based on a fulfilled screening of compounds containing the adenosine moiety (the fragment responsible for the combination with METTL3 at the binding sites for SAM). In 2020, Bedi et al.^645^ performed a series of docking studies on over 4000 adenosine-moiety compounds, identifying seven molecules with potential binding affinity to METTL3; however, their inhibitory effects in vivo were minimal. Similarly, Moroz-Omori et al.^646^ and Dolbois et al.^647^ reported on adenine-based libraries, identifying UZH1a and UZH2 as compounds that occupy the catalytic pocket of METTL3, suggesting their role as potential competitive inhibitors in vitro. Cpd-564, an METTL3 inhibitor identified from ChemDiv and MCE screening libraries, has shown significant reno-protective effects in mouse models of acute kidney injury induced by cisplatin and ischemia-reperfusion.^648^ Coptisine chloride, identified via molecular docking-based virtual screening from the Vitas-M chemical library, displayed high affinity to METTL3, exerting competitive inhibitory effects by occupying the SAM binding pocket.^649^ STM2457 and STM3006 are novel small molecules that bind non-covalently to the catalytic center of METTL3, reducing its enzymatic activity.^650,651^ Specifically, STM2457 has demonstrated promising antitumor effects and tolerability in mouse models, improving drug resistance to chemotherapy.^650,652–654^ In comparison, although STM3006 exhibits enhanced cellular potency, its in vivo efficacy is constrained by its shorter half-life.^651^ In 2023, STC-15, an oral inhibitor optimized from STM2457, became the first and the only RNA m6A target drug to be applied in phase I clinical trials (NCT05584111). Through detailed studies on the spatial structure of the catalytic domain of METTL3, a series of branched, Y-shaped molecules are designed. These were synthesized by integrating chemical fragments from the most effective inhibitors, resulting in molecules with selectivity and binding affinities surpassing those of STM2457, the only commercially available METTL3 inhibitor.^655^ This advancement not only underscores the potential of METTL3 as a therapeutic target but also guides future drug design. Additionally, several natural products with METTL3-inhibitory capabilities have been identified. Quercetin, known as a DNMT inhibitor, has been found to interact with the adenosine moiety pocket in METTL3, forming a stable complex that reduces its catalytic activity.^656^ This interaction decreases METTL3 hyperactivation and lowers m6A levels in protein kinase D2 mRNA, improving insulin sensitivity under palmitic acid stimulation—a benefit in hyperinsulinemia conditions.^657^ Other natural compounds like berberine and curcumin, also noted for DNMT/HDAC inhibition, have shown METTL3 inhibitory activity, though their mechanisms require further clarification.^658,659^ Moreover, molecules F039-0002 and 7460-0250 have been designed to target METTL3’s catalytic pocket, showing potential in treating inflammatory bowel disease.^660^ Several candidates identified through silico analysis of South African natural products—SANCDB0370, SANCDB0867, and SANCDB1033—also exhibit METTL3 inhibitory properties, with further validation needed.^661^ More recently, Li et al.^662^ designed a stapled peptide inhibitor, RSM3, targeting the PPI at the METTL3-METTL14 interface. This inhibitor offers a unique approach compared to other small-molecule competitive inhibitors, providing a novel avenue for therapeutic intervention.

Allosteric inhibitors prevent METTL3/14-dependent m6A methylation in a non-competitive manner. To date, three allosteric inhibitors have been identified. The first two allosteric inhibitors are CDIBA and CDIBA-43n, which initially function as cytosolic phospholipase A2 inhibitors preventing inflammation. They show an inhibitory effect in the presence of METTL3/14 complex, instead of separate METTL3 and METTL14 subunits.^663^ The third compound, eltrombopag (previously mentioned as a TET agonist), is recently reported to bind with the METTL3 subunit at an allosteric site and has shown potential in treating AML.^663,664^

Metformin, traditionally used as a first-line treatment for T2DM, has recently been found to inhibit METTL3 expression, possibly contributing to its beneficial effects in patients with malignant tumors.^665^ The role of metformin in inhibiting METTL3 expression, at the post-transcriptional level, in breast tumors, is first reported in breast cancer.^666^ Subsequently, the application of metformin is also found to inhibit METTL3 expression at the transcriptional level, mediated by the recruitment of DNMT.^667^ This dual action of metformin, combined with chemotherapy, offers potential benefits for patients resistant to traditional chemotherapy, potentially mitigating poor prognoses.^667,668^ Given its safety profile, metformin is a promising candidate as an epigenetic drug targeting METTL3.

##### METTL3 agonists

While research has predominantly focused on METTL3 inhibitors, there is also interest in agonists, given their potential benefits in DNA damage repair, tumor therapies, and regenerative medicine.^669–672^ In 2019, Selberg et al.^673^ predicted interactions between four small-molecule ligands with METTL3 involving piperidine and piperazine rings, similar to SAM’s binding. These interactions enhanced cell viability and promoted proliferation, although differing onset times among the compounds suggest the need for further development of more effective METTL3 complex activators.^673^ Melatonin seems to act as an agonist of METTL3. Lv et al.^674^ proposed that melatonin pretreatment can upregulate the expression level of METTL3, restore m6A levels in spermatogonial stem cells, and help them resist the destructive effect of Cr(VI) on reproductive function. However, this viewpoint has recently been questioned. In mouse models with colon inflammation, melatonin inhibits METTL3 expression through melatonin receptor 1B.^675^ Further research is necessary to clarify melatonin’s role in METTL3 regulation.

#### Targeting the eraser of m6A: FTO and ALKBH5

FTO and alkB homolog 5 (ALKBH5) are established m6A erasers, each playing significant roles in epigenetic regulation. FTO is primarily involved in energy homeostasis, demethylating m6A in various RNA species, including cellular mRNA, which impacts multiple biological processes.^676^ ALKBH5 not only demethylates m6A-marked mRNA but also m6A-marked single-stranded DNA (ssDNA), influencing oncogenic or tumor-suppressive activities.^677^ Over the years, numerous small molecules targeting these m6A writers have been identified and designed, showing promising therapeutic efficacy in vitro and in vivo and advancing the development of epigenetic drugs.

##### FTO inhibitors

Current strategies for developing FTO inhibitors are multifaceted. Based on the spatial structure of FTO, competitive or non-competitive inhibitors that bind to FTO covalently or non-covalently have been developed. With a deeper understanding of FTO functionality, metabolites in vivo possibly related to FTO have been identified, represented by D-2-HG, a metabolite produced by mutant IDH.^678^ Furthermore, exploring the mechanisms underlying medical agents that treat FTO-related diseases provides a theoretical foundation for drug discovery.^679^ Subsequently, optimizing the molecular structures of these initially detected compounds will contribute to the development of FTO inhibitors with high selectivity and inhibitory effects, providing promise for the clinical application of FTO inhibitors in the future. Here, we summarize the typical drugs that inhibit FTO, which are the foundation for developing novel inhibitors through constant iterations.

In 2012, the first FTO inhibitor, rhein, was identified. Rhein impairs FTO activity by disrupting its interaction with ssDNA at the catalytic domain.^680^ Although rhein increases m6A levels in vitro, its weak selectivity and low inhibitory efficacy have limited its clinical potential, highlighting the need for more effective FTO inhibitors.^680^ New FTO inhibitors need to be designed to overcome these drawbacks. Meclofenamic acid (MA), an FDA-approved nonsteroidal anti-inflammatory drug, binds selectively to similar sites on FTO.^681^ MA and its prodrug, MA2, have shown promising results in reversing tumor progression and enhancing the efficacy of chemotherapeutic drugs, significantly prolonging survival.^681,682^ Inspired by MA, various compounds have been developed, such as the fluorescein derivative FL1, which retains the benzyl carboxylic acid structure critical for interaction with FTO. The complex formed between FTO and FL1 inhibits the enzyme’s activity and facilitates the study of FTO-related signaling pathways through fluorescein labeling.^683^ Other optimization molecules include GNPIPP12-MA,^684^ 13a,^685^ FB23/FB23-2,^686^ Dac-51/Dac-85,^687^ ZLD115,^688^ and FTO-02/FTO-04/FTO-43.^689,690^ These compounds significantly improve MA in inhibitory activity, cell permeability, and biosafety while reducing off-target effects and potential resistance. Another similar mechanism inhibitor is diacerein, a structural analog of rhein, which has shown antitumor effects in breast cell lines.^691^

In addition to interfering with the interaction between ssDNA and FTO, inhibitors of this enzyme also compete with cofactors such as α-KG and iron(II). For instance, fumarate hydrazide 2 and compounds with the aminohydroxyfuranone core exemplify this approach.^692,693^ Furthermore, the discovery of N-CDPCB, a competitive inhibitor that binds to non-conserved fragments of FTO, provides novel insights into the development of inhibitory agents. Mechanistically, compounds like benzene-1,3-diol and 4-Cl-1,3-diol are crucial in mediating and enhancing the specific interaction between FTO and N-CDPCB.^694^ Additional potential inhibitors, such as CHTB and radicicol, have been identified through virtual screening; these compounds have similar structures.^695,696^ However, related evidence is lacking to exhibit their efficacy. Moreover, Su et al.^697^ reported on CS1 and CS2, which tightly bind to the catalytic pocket of FTO, activating immune checkpoint genes and reversing immune evasion in tumor diseases. Clausine E, another FTO inhibitor, targets the enzyme’s hydrophobic cavity, exhibiting antitumor activity.^698^

These ongoing discoveries provide a deeper understanding of the diverse structures of molecules interacting with FTO and their mechanisms of action, promoting large-scale virtual screenings to identify more potential inhibitors. For example, mupirocin, entacapone, compounds “18,077” and “18,079”, several quinolone derivatives, and a series of 1,2,3-triazole analogs have been identified as potential FTO inhibitors.^699–703^ Notably, quinolone derivatives and their antitumor properties have shown the potential to improve symptoms in neurodegenerative diseases by inhibiting FTO activity.^702^ These findings broaden the potential clinical applications of FTO inhibitors.

##### FTO agonists

Recent studies have identified that certain tricyclic antidepressants, such as imipramine and amitriptyline, exert their antidepressant effects by activating FTO in N2a cells.^704^ This emerging area of research highlights the potential therapeutic benefits of FTO activators and calls for more attention to their development and evaluation.

##### ALKBH5 inhibitors

The RNA demethylase ALKBH5 is recognized as a pro-oncogene, playing a vital role in the post-transcriptional regulation of various targets in cancer biology.^677^ Interest in targeting ALKBH5 for therapeutic purposes has significantly increased. We classify the identified ALKBH5 inhibitors into three main categories based on their mechanisms of action. The first category comprises typical competitive inhibitors that compete with cofactors for binding sites. These agents consist of IOX1 (also known as a TET/KDM inhibitor), MV1035, and Ena21, which exhibit the therapeutic potential of targeting ALKBH5 in antitumor therapies.^705–707^ Compounds that non-covalently interact with the active pocket of ALKBH5 are classified as the second group. Through structure-based virtual screening and optimization, the current compounds include DDO-2728, 2-((1-hydroxy-2-oxo-2-phenylethyl)thio)acetic acid, and 4-((furan-2-ylmethyl)amino)tetrahydropyridazine-3,6-dione.^708,709^ The third category includes molecules that bind to the m6A-binding pocket of ALKBH5, directly disrupting the interaction between the enzyme and its substrates. For instance, compounds 20 m and TD19 are representative of this type.^710,711^ Some compounds still exist whose potential mechanisms for inhibiting ALKBH5 have not been elucidated, such as ALK-04, Ena15, ZINC78774792, and ZINC00546946, although their antiproliferative effects have been revealed in vitro and in vivo.^707,712,713^ In brief, the continued development and in-depth research into ALKBH5 inhibitors hold significant potential for disease treatment, necessitating further efforts.

#### Targeting the reader of m6A: IGF2BP and YTH domain family

The discovery of m6A readers with specific motifs has spurred significant interest in developing drugs targeting these proteins, expanding the possibilities for therapeutic interventions.

##### IGF2BP inhibitors

Insulin-like growth factor 2 mRNA-binding proteins (IGF2BPs) are newly identified m6A readers that enhance the stability and maintenance of their target mRNAs.^714^ IGF2BP plays an oncogenic role in various cancers, making its inhibition a promising strategy for antitumor therapy.^715^

Six IGF2BP inhibitors have been developed, demonstrating antitumor effects by disrupting IGF2BP-RNA interactions. BTYNB, the first identified IGF2BP1 inhibitor, suppresses melanoma and ovarian cancer cell proliferation by blocking IGF2BP1’s interaction with c-Myc mRNA.^716^ BTYNB’s therapeutic effects are being studied across various tumor models, including esophageal squamous carcinoma,^717^ neuroblastoma,^718^ and cholangiocarcinoma.^719^ CWI1-2 and JX5 are novel IGF2BP2 inhibitors with antileukemic activities that inactivate the Notch1 signaling pathway. CWI1-2 forms a hydrophobic interaction with IGF2BP2’s RNA-binding core, while JX5 binds tightly to the protein. Further research is needed to enhance their safety and reduce cytotoxicity.^720,721^ Cucurbitacin B, a natural product, exerts a pharmacological allosteric effect on IGF2BP1. In hepatocellular carcinoma mouse models, it modifies IGF2BP1’s configuration, reducing its efficacy.^722^ Another compound, “7773,” specifically disrupts the IGF2BP1-Kras mRNA interaction, effectively inhibiting IGF2BP1’s pro-oncogenic activity.^723^ Isoliquiritigenin is the only small molecule identified targeting IGF2BP3.^724^ Derived from the Chinese herb licorice, it downregulates IGF2BP3 expression, showing promise in treating non-small cell lung cancer.^724^

##### YTH domain family inhibitors

YTH domain family (YTHDF) comprises a group of readers featuring a YTH domain at the C-terminus. This domain forms a hydrophobic pocket essential for recognizing m6A modifications.^725^ Elevated levels of YTHDF proteins have been associated with the progression of various cancers.^726^ Conversely, reducing these proteins can synergistically enhance the effectiveness of ionizing radiation and anti-PD-L1 therapies in reducing cancer burdens.^727,728^ This underscores the potential of YTHDF inhibitors as a promising direction for improving antitumor treatments.

The binding sites between YTHDF proteins and m6A modifications are primary targets for most YTHDF inhibitors.^729^ The successful elucidation of the crystallographic structures of the YTH domains in YTHDF proteins has provided critical opportunities for drug design.^730^ High-throughput screening technology has identified three small molecules—ebselen, DC-Y13, and DC-Y13-27—as effective YTHDF inhibitors.^727,731^ Ebselen targets YTHDF1 and YTHDF2, either covalently or non-covalently binding to the YTH domain.^731^ DC-Y13 and DC-Y13-27, particularly the latter, act as selective inhibitors of YTHDF2, offering therapeutic benefits.^727^ Additionally, studies have shown that disrupting O-GlcNAcylation of YTHDF proteins can also decrease their stability and enzymatic activities, providing new avenues for identifying YTH-inhibiting small molecules.^732,733^

### Epigenetics-targeted drugs and chromatin remodeling

SWI/SNF complexes are intricate multimeric structures composed of diverse, variable subunits that play distinct roles, emphasizing the importance of personalized characteristics and frequent mutations in these subunits in various human diseases. Recently, the design of small molecules targeting different components of the SWI/SNF complex has expanded, yielding numerous potential therapeutic interventions. Those that have progressed to clinical trials are detailed in Table 6.

**Table 6:** Summary of chromatin remodeling-targeted drugs for different diseases in clinical trials

Drug	Target(s)/mechanisms(s)	Condition(s)	Status/outcome(s)	Phase(s)	Other intervention(s)/drug(s)	Study ID
FHD-286	SMARCA4/2 allosteric inhibitor	Metastatic uveal melanoma	Terminated (due to business reasons)	Phase I	—	NCT04879017
FHD-286	SMARCA4/2 allosteric inhibitor	AML, MDS, CMML	Recruiting	Phase I	With or without low-dose Cytarabine or Decitabine	NCT04891757
PRT3789	PROTACs-based SMARCA2 degrader	NSCLC and other solid tumors with SMARCA4 gene mutation	Recruiting	Phase I	With or without Docetaxel	NCT05639751
FHD-609	PROTACs-based BRD9 degrader	Advanced synovial sarcoma	Terminated (due to sponsors’ decision)	Phase I	—	NCT04965753
CFT8634	PROTACs-based BRD9 degrader	Synovial sarcoma and other SMARCB1-perturbed soft tissue sarcomas	Terminated (no significant clinical activity with CFT8634 as a single agent)	Phase I/II	—	NCT05355753

*AML* acute myeloid leukemia, *BRD9* bromodomain containing 9, *CMML* chronic myelomonocytic leukemia, *MDS* myelodysplastic syndrome, *NSCLC* non-small cell lung cancer, *PROTAC* proteolysis-targeting chimeras, *SMARCA4* SWI/SNF-related, matrix-associated, actin-dependent regulator of chromatin A4

The active DNA-dependent ATPase A domain inhibitor (ADAADi) was the first discovered inhibitor targeting the SWI/SNF complex. It was identified during studies on mammalian cell resistance to certain antibiotics in vitro.^734^ ADAADi binds to specific motifs in the enzyme complex, inducing conformational changes that inhibit SWI2/SNF2’s catalytic activities.^734^ Currently, ADAADi shows promising therapeutic effects in prostate cancer in preclinical studies, laying the groundwork for further development of SWI/SNF-targeted epigenetic drugs.^735^

Research has also focused on specific inhibitors targeting the SWI/SNF-related, matrix-associated, actin-dependent regulator of chromatin A4 (SMARCA4) and its paralog SMARCA2, which are DNA-stimulated ATPases within the SWI/SNF complexes. SMARCA4, commonly mutated in various tumors, is associated with reduced sensitivity to traditional cancer treatments.^736,737^ Inhibiting SMARCA4/2 is an effective strategy for curbing tumor growth and improving patient outcomes. Papillon et al.^738^ reported the earliest selective allosteric inhibitors of the SMARCA4/SMARCA2 subunits, with confirmed effects on pediatric H3K27M diffuse midline glioma and AML in both in vivo and in vitro settings.^739,740^ FHD-286, a novel orally bioavailable SMARCA4/SMARCA2 allosteric inhibitor, has shown preclinical efficacy. Combined treatment with FHD-286 and other epigenetic drugs, such as decitabine, BET inhibitors, and menin inhibitors, has demonstrated synergistic effects in reducing AML burden without significant toxicity.^741^ Notably, FHD-286 has entered clinical development for treating various malignant tumors, including metastatic uveal melanoma (NCT04879017) and several malignant hematological disorders (NCT04891757).

An alternative approach to inhibiting SMARCA4/SMARCA2 involves using specific inhibitors that target their BDs. This strategy extends to other BD-containing proteins within the SWI/SNF complexes, such as polybromo-1 (PBRM1), BD containing 7 (BRD7), and BRD9, also considered promising targets for epigenetic drug development. Notably, SMARCA4/SMARCA2 and BRD9/BRD7 each contain one BD, whereas PBRM1 contains six tandem BDs, providing numerous potential interaction points for inhibitors.^742^ Current research primarily focuses on inhibitors for family VIII BD in SMARCA4/SMARCA2 and PBRM1, with four major classes of inhibitors reported: salicylic acid fragment hits such as PFI-3;^743–745^ aminopyridazines represented by GNE-064;^746^ quinazolinones represented by LM146,^747,748^ and dihydroquinazolinones represented by compound16 and GNE-235.^749,750^ These inhibitors are categorized as either pan-inhibitors, affecting multiple proteins, or selective inhibitors, targeting specific proteins. PFI-3, its analogs, and GNE-064 are pan-inhibitors, whereas LM146 shows a higher affinity for SMARCA2, and compound16 and GNE-235 are selective for PBRM1. The therapeutic applications of these inhibitors, particularly the pan-inhibitors, have been extensively studied in various diseases.^751–755^ However, the efficacy of PFI-3 as a standalone treatment for malignancies has been less satisfactory. The application of compound16, on the other hand, demonstrates promising therapeutic effects in PBRM1-dependent prostate cancer, suggesting its potential as a foundational treatment for PBRM1-driven cancers.^749^ As for the other molecules, though their binding ability and inhibitory effects have been validated at the molecular level, sufficient evidence is still lacking in vivo or in vitro to demonstrate their clinical value. Furthermore, targeting family IV BD of BRD9 and BRD7 has led to the development of many selective inhibitors. Current research includes inhibitors like BI-7271,^756^ BI-7273,^756^ BI-9564,^756^ I-BRD9,^757^ iBRD9,^758^ GNE-375,^759^ and newly identified inhibitors developed through integrated computational approaches.^760^ Selective inhibitors for BRD7, such as 1-78 and 2-77,^761^ and molecules like LP99, TP-472, 4-acylpyrroles, and GSK6776, which inhibit both BRD9 and BRD7, are being evaluated for their therapeutic effects in various pathologies.^762–765^

PROTAC technology plays a significant role in developing SWI/SNF inhibitors, with novel agents such as AU-24118 and AU-15330 being tested in preclinical studies and clinical trials. AU-24118 and AU-15330 are degraders targeting family VIII BD in SMARCA4/SMARCA2 and PBRM1, which are valuable tools in castration-resistant prostate cancer treatment.^766,767^ AU-24118 has shown promise in inducing tumor regression at therapeutic doses.^766^ However, long-term treatment at high doses can lead to mutations in the BD and overexpression of ATP-binding cassette subfamily B member 1 (ABCB1), which contributes to drug resistance development.^766^ Combining these treatments with ABCB1 inhibitors could potentially mitigate resistance to SMARCA4/SMARCA2 inhibitors in vivo. Additionally, applying PROTACs to previously reported inhibitors can enhance their selectivity and reduce off-target effects. For instance, the linkage of BI-7273 with an E3 ubiquitin ligase has led to the design of dBRD9-A, the first BRD9-directed degrader, which is undergoing optimization.^768,769^ The current focus on dBRD9-A, which is being tested for its efficacy in AML, MM, and interferon-induced inflammation in animal models, highlights its potential as a promising therapeutic for both tumor and non-tumor conditions.^768,770,771^ Additional PROTACs-based SWI/SNF inhibitors, such as those derived from dihydropyrrolo-quinazolin scaffolds (targeting SMARCA4, SMARCA2, and PBRM1),^747^ A947 (targeting SMARCA2),^772^ VZ-185 (targeting BRD9 and BRD7),^773^ CFT8634 as well as FHD-609 (targeting BRD9), further illustrate the breadth of ongoing research.^774^ Notably, FHD-609 have recently advanced to clinical trials for synovial sarcoma (NCT04965753), underscoring the critical role of drug development targeting mutations in SWI/SNF complexes. Moreover, CFT8634 was originally planned for investigation in a phase 1/2 clinical trial of synovial sarcoma and other SMARCB1-perturbed soft-tissue sarcomas (NCT05355753). However, the clinical trial was terminated because of the less significant clinical activity of CFT8634 as a single agent. Considering the prevalence of mutations in SWI/SNF complexes in cancers, continued research into the in vivo therapeutic effects, potential applications, and long-term risks of these drugs is essential for assessing their clinical utility.

### Epigenetics-targeted drugs and non-coding RNA

A deep understanding of ncRNA’s role in disease progression, particularly in various cancers, has led to innovative epigenetic strategies for disease management.^775^ RNA interference (RNAi) technologies, which utilize small double-stranded RNA to selectively interact and degrade specific intracellular RNAs, mimic gene deletion phenotypes.^776,777^ RNAi-based therapies targeting ncRNA are categorized based on their action mechanisms and intended outcomes: silencing overexpressed ncRNAs to curb disease-related expressions, restoring downregulated ncRNAs to regain lost functions, and blocking ncRNA localization to prevent ncRNA from functioning by interfering with its subcellular localization. RNAi-oriented drugs altering ncRNA patterns have been widely studied and applied in clinical practice (Table 7). Herein, we summarize the emerging technologies for ncRNA-targeted agent development, aiming at supplementing the current understanding of drug design.

**Table 7:** Summary of non-coding RNA drugs for different diseases in clinical trials

Drug	Target/Mechanism	Condition(s)	Status/outcome(s)	Phase(s)	Other intervention(s)/drug(s)	Study ID/references
MRG-106	MiR-155 inhibitor	CTCL, MF, CLL, DLBCL, and ATCL	Completed (unpublished)	Phase I	Stable background therapy (simultaneously applied in few participants)	NCT02580552
MRG-106	MiR-155 inhibitor	CTCL, MF	Terminated (due to business reasons)	Phase II	Vorinostat (active comparator)	NCT03713320
MRG-106	MiR-155 inhibitor	CTCL, MF	Terminated (due to eligible subjects receiving treatment in a crossover arm of NCT03713320)	Phase II	—	NCT03837457
MRG-110	MiR-92a-3p inhibitor	Healthy volunteers	Completed (unpublished)	Phase I	Placebo-controlled	NCT03603431
MRG-110	MiR-92a-3p inhibitor	Healthy volunteers	Completed (significant inhibition on targeted miRNA in vivo)	Phase I	Placebo-controlled	NCT03494712^1151^
RG-012	MiR-21 inhibitor	AS	Completed (unpublished)	Phase I	—	NCT03373786
MRG-201	MiR-29b mimic	Healthy volunteers	Completed (unpublished)	Phase I	Placebo-controlled	NCT02603224
MRG-201	MiR-29b mimic	Keloid	Completed (exhibits therapeutic effects and manageable adverse events)	Phase II	Placebo-controlled	NCT03601052
MRX34	MiR-34a mimic	Liver cancer, SCLC, lymphoma, melanoma, MM, RCC, NSLCL	Terminated (due to serious immune-related adverse events)	Phase I	—	NCT01829971^804,1152^
MRX34	MiR-34a mimic	Melanoma	Withdrawn (due to immune-related serious adverse events in the phase I study)	Phase I/II	Dexamethasone premedication	NCT02862145
TargomiRs	MiR-16 mimic	MPM, NSCLC	Completed (exhibits acceptable safety profile and early signs of therapeutic activity)	Phase I	—	NCT02369198^1153^
INT-1B3	MiR-193a-3p mimic	Advanced solid tumors	Terminated (due to the insufficient funding)	Phase I	—	NCT04675996

*AS* Alport syndrome, *ATCL* adult T cell leukemia/lymphoma, *CLL* chronic lymphocytic leukemia, *CTCL* cutaneous T cell lymphoma, *DLBCL* diffuse large B cell lymphoma, *MF* mycosis fungoides, *Mi-R microRNA* MM multiple myeloma, *MPM* malignant pleural mesothelioma, *NSCLC* non-small cell lung cancer, *RCC* renal cell carcinoma, *SCLC* small cell lung cancer

Significant advancements in molecular editing and delivery systems have bolstered the clinical viability of these innovative ncRNA-targeted therapies.^778,779^ One pivotal development has been the chemical modification of synthetic nucleic acids to enhance their stability and delivery efficiency. The initial focus on replacing phosphodiester bonds with phosphorothioate has been a cornerstone in numerous FDA-approved oligonucleotide therapies,^780^ although concerns about inflammation and toxicity in vivo have prompted research into alternative modifications for RNA-targeted based on RNAi.^781^ Over the years, various modification strategies have emerged, including those based on 2’-O-methyl, 2’-O-methoxyethyl, 2’-fluoro, and n-acetylgalactosaminyl, aiming to preserve the therapeutic attributes of nucleic acids while enhancing their stability.^782^ Locked nucleic acids (LNAs) represent a notable innovation, linking the 2’ and 4’ carbons of ribose rings with methylene bridges, thus improving hybridization affinity and resistance to nucleases.^783^ Cobomarsen, an LNA-based inhibitor targeting miR-155, exemplifies this technology’s potential, having shown promising results in preclinical studies for hematological malignancies and solid tumors and exhibiting positive effects in mycosis fungoides patients.^784–788^ In addition, the therapeutic efficacy of cobomarsen has been further validated in patients with mycosis fungoides, indicating well-tolerated and positive clinical potentials (NCT03713320).

Furthermore, integrating nanomedicine-based delivery systems, such as lipid-based, polymeric, inorganic, and biomimetic nanoparticles, has significantly advanced the development of RNAi drugs. These delivery techniques enhance the stability and bioavailability of oligonucleotide drugs and improve their efficacy in modulating target ncRNA expression and functionality.^789^ The recent emphasis on nanoparticles designed for targeted delivery of therapeutic nucleic acids to specific subcellular organelles marks a significant advancement in ncRNA therapy. As ncRNAs are present not only in the cytoplasm but also the nucleus and various organelles, targeting these subcellular locations can enhance the efficacy of treatments.^790,791^ Researchers are exploring opportunities to integrate subcellular organelle-targeting signals into nanoparticle delivery systems. Current studies have reported RNAi nanoparticle systems designed to target the nucleus, mitochondria, endoplasmic reticulum, and Golgi apparatus.^792–795^ Though nucleus-targeted nanoparticles have been studied in regulating ncRNAs, only a few of them have proposed the incorporation of nucleus-targeting TAT peptide and nucleus-targeting peptide amphiphile into nanoparticle delivery systems to achieve active transportation.^792^ Many researchers have only reported the high concentrations of therapeutic oligonucleotides in the nucleus without elucidating the mechanisms involved in nucleus-targeting. This approach leaves a gap in our understanding of the complex and precise intracellular delivery processes that involve biomembrane systems and cytoskeletal interactions.^796^ The variability in cell types, delivery materials, and therapeutic nucleic acids means that successful results in specific contexts may not universally apply, highlighting the challenges in translating these strategies from experimental to clinical settings.

Additionally, the integration of clustered regularly interspaced short palindromic repeats (CRISPR)/CRISPR-associated system (CRISPR/Cas) technologies, particularly CRISPR/Cas9, into ncRNA research offers novel avenues for manipulating ncRNA expression, including lncRNAs and microRNAs (miRNAs).^797–800^ CRISPR/Cas9’s flexibility and high specificity make it an advantageous tool for gene-targeted cancer therapies, such as CRISPR interference, activation, and knockout strategies, now moving into preclinical trials. This gene-editing technology promises to enhance the efficacy of traditional cancer treatments and aims to minimize off-target effects, thus fostering the development of personalized medicine.^801,802^

However, several challenges persist despite the growing number of ncRNAs identified as potential therapeutic targets. Technological advancements are still needed, and safety concerns must be rigorously addressed.^803^ The premature termination of the Phase I clinical trial for MRX34, a liposomal mimic of miR-34a, due to severe immune reactions and fatalities, underscores the critical need for comprehensive preclinical data and cautious progression to clinical trials.^804^

## Practical challenges in the epigenetic drug development and application

Massive efforts have been made in epigenetics-targeting drug development, whether approved in different countries for the treatment of specific indications or currently identified and further evaluated in fundamental experiments or clinical trials, exhibiting notable potential. However, there are still practical issues that should be dealt with when applying them to a large scale in clinical practice.

First of all, a thorough understanding of epigenetic mechanisms is crucial for successfully applying epigenetic-targeted drugs. Alterations in epigenetic enzymes can change DNA, histones, or chromatin structures, impacting cellular processes like transcription, replication, and DNA repair. Even minor modifications in epigenetic enzyme activity can significantly affect cellular functions. Therefore, deepening our understanding of epigenetic biology is essential. However, gaps remain in our knowledge, particularly regarding the roles of epigenetic regulation and its proteins in mammals, such as new DNA modifications like m6A, RNA modifications beyond m6A, novel reader domains, and PPI networks related to epigenetic regulation. These areas represent potential targets for novel epigenetic drugs but require further exploration.^805–810^ Second, many molecules identified through virtual screening and molecular docking techniques are primarily used as molecular probes to study enzyme localization and function. However, transitioning from a potent molecular probe to a viable therapeutic agent involves rigorous in vivo evaluation to confirm their inhibitory effects and therapeutic potential. This step is crucial as it determines whether these compounds can be safely and effectively used in clinical settings.^811,812^ These small molecules are potent probes to detect the localization and function of targeted enzymes, and their potential for medical use needs confirmation. Notably, another challenge that needs to be overcome is associated with the precise localization and targeted activity of epigenetics-targeted drugs is critical for enhancing their clinical efficacy and reducing potential side effects. The need for precise delivery of epigenetic drugs to subcellular structures is underscored by the diverse localizations and functions of their target enzymes and ncRNAs. For instance, enzymes responsible for histone modifications are found both in the nucleus, where they modify histones, and in the cytoplasm, where they regulate non-histones and related signaling pathways at the post-translational level.^813,814^ Similarly, the function of ncRNAs depends significantly on their localization and distribution within the cell.^790^ To address these challenges, a deeper understanding of the intracellular trafficking mechanisms is required. Additionally, developing sophisticated drug delivery systems, such as lipid-based nanoparticles or targeted delivery vehicles, can enhance the specificity and efficiency of these therapies by directing them to their precise intracellular sites of action. Furthermore, the challenge of achieving selective inhibition within the HDAC family exemplifies the broader issue of specificity in drug design. Many drugs targeting epigenetic enzymes exhibit pan-inhibitory effects, leading to significant off-target effects and adverse reactions, particularly when the drugs indiscriminately affect multiple members of an enzyme family.^408^ This necessitates the development of more selective inhibitors that specifically target single proteins or subfamilies of proteins. By focusing on selective inhibition, researchers can potentially improve the safety and efficacy of these treatments, minimizing unwanted interactions and enhancing their therapeutic impact.

Importantly, the possibility of developing resistance to epigenetic-targeted drugs, which is another factor limiting their further application, cannot be ignored. Several studies have investigated the mechanisms involved in the development of resistance to epigenetic agents in different cancers.^815–817^ The activation of certain signaling pathways and gene mutations in tumor cells play an indispensable role in inducing the development of drug resistance to epigenetic agents. Research has shown that the activation of enhanced Wingless/Integrated (Wnt)/β-catenin signaling contributes to developing resistance to BET inhibitors in leukemia cells. However, inhibition of this pathway helps rescue drug sensitivity in vitro and in vivo.^818,819^ Furthermore, enhanced activity of the protein kinase B (AKT)/mTOR complex 1 (mTORC1) signaling pathway is also responsible for drug resistance to BET inhibitors in prostate cancer.^820^ In another study on HDAC resistance in solid tumors, the potential role of the activation of some kinases and downstream pathways was also reported.^821^ Additionally, altered TME may be one of the culprits in promoting drug resistance. Tumor-associated macrophage (TAM), a pivotal mediator in inducing tumor cells to develop resistance to traditional antitumor therapies, has recently been proposed to be involved in the occurrence of epigenetic drug resistance.^822,823^ A 2020 study on triple-negative breast cancer reported that interleukin-6 (IL-6) and IL-10 derived from TAM activated STAT3 signaling in tumor cells, conferring them with drug resistance to BET inhibitors.^823^ Additionally, these findings pave a theoretical foundation for the combination of epigenetics-targeted drugs and other pharmaceutical molecules to optimize their long-term efficacy.^824^ The relationship between drug resistance to epigenetic agents and mutations in certain genes is observed in tumor cells, especially in the case of applying EZH2 and IDH inhibitors.^59,825,826^ Based on this idea, the loss-of-function mutation of specific genes by CRISPR/Cas techniques may provide better platforms for coping with drug resistance.

In conclusion, for epigenetics-targeted drugs, it is crucial to balance pharmacokinetics (how the drug is processed in the body), tolerability (how well the drug is tolerated), and therapeutic efficacy (how to avoid off-target off-target toxicities and resistance). Developing optimal dosing regimens that maximize efficacy while minimizing side effects and resistance requires a thorough understanding of the drug’s behavior in the body, including its absorption, distribution, metabolism, and excretion. Innovative dosing strategies, possibly involving controlled release formulations or real-time monitoring of drug levels, could play a vital role in achieving this balance. Continued research into the biological and pathological roles of targets for epigenetic drugs is essential.

## Developing trends and forthcoming prospects in epigenetics-targeted drugs

**Fig. 5:**
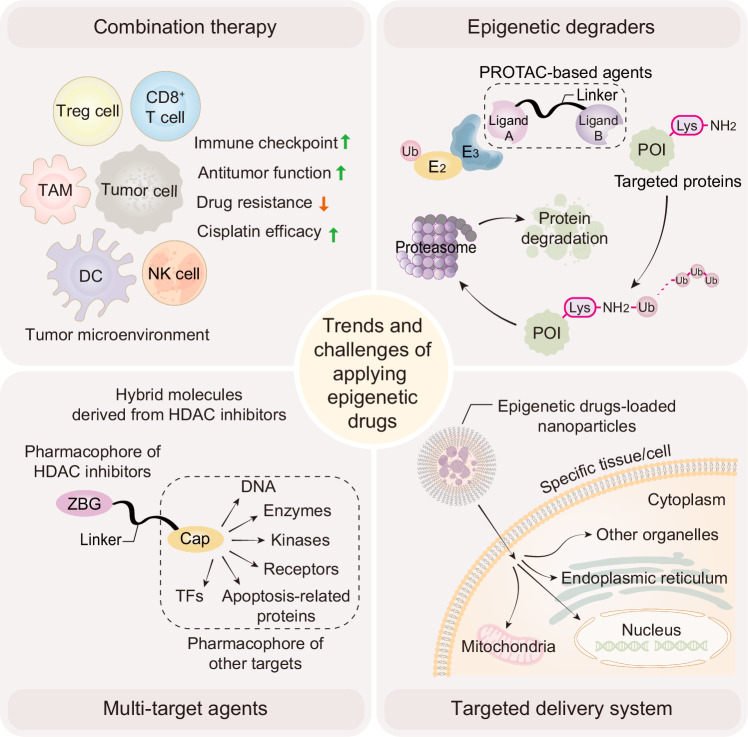
The promising trends and practical challenges in the clinical application of epigenetics-targeted drugs. From the perspective of clinical practice, epigenetic agents are expected to become promising adjuvants in combination with traditional antitumor therapeutics, contributing to superior efficacy and decreased resistance. Based on this idea, multitarget anticancer agents inhibiting both HDAC and other pathological molecules have gained much attention as a new strategy. Further, epigenetic degraders based on PROTAC or other techniques responsible for TPD help supplement the catalytic function of epigenetic inhibitors. Notably, the successful transport of epigenetic regulators to specific tissues or cells, or even the finite subcellular structures, is the prerequisite for exerting therapeutic effects. The further optimization of different types of nanoparticles makes them inspiring tools for the delivery system

Research on epigenetics-targeted drugs has progressed rapidly, with a growing focus on their potential as next-generation clinical candidates. Current trends, illustrated in Fig. 5, emphasize the synergy between these agents and other therapeutic modalities such as chemotherapy, radiotherapy, kinase inhibitors, and immunotherapy. This integration promotes precision medicine and personalized treatment strategies and enhances the overall effectiveness of cancer therapies.^827,828^ Furthermore, developing epigenetic degraders, which can hydrolyze targeted proteins, complements the inhibitory functions of traditional epigenetic drugs.^829^ Notably, the swift advancement of sequencing technology has empowered the detection of epigenetic irregularities with growing efficacy, substantially enhancing the integration of epigenetics into personalized medicine.

### Epigenetics-based combination therapy in cancer cells

The integration of epigenetics-targeted drugs with conventional cancer therapies—such as chemotherapy, targeted therapy, immunotherapy, and hormone therapy—is emerging as a promising strategy for cancer treatment. Increasing experimental studies and clinical trials are assessing the safety and efficacy of various combination regimens. The benefits of these combinations can be categorized into two primary aspects:

Epigenetic drugs can synergize with other cancer therapies by modulating the metabolic and pathological characteristics of cancer cells, immune cells, and stromal cells within the TME.^830^ Although immune checkpoint inhibitors (ICIs), which target immune checkpoint proteins (ICPs), such as PD-L1, PD-1, and CTLA-4, show significant potential, their effectiveness may be limited by factors such as insufficient antigen presentation and suboptimal T cell responses in the TME.^831,832^ Epigenetic modifications can enhance the expression of tumor antigens and ICPs, overcoming these limitations. The use of epigenetic therapies not only disrupts immunosuppressive pathways but also enhances the recruitment of tumor-reactive immune cells, resulting in synergistic effects with ICIs.^136,833–835^

Interfering with aberrant epigenetic features is crucial for combating drug resistance, a major challenge in oncology. Chemoresistance often correlates with changes in DNA methylation and histone acetylation, among other epigenetic characteristics.^139,836,837^ Combining chemotherapeutic agents with epigenetic drugs has become an important strategy to address resistance,^838,839^ also helping to mitigate chemotherapy-related side effects.^840,841^ Additionally, reversing epigenetic alterations in chemoresistant tumor cells can restore their sensitivity to conventional therapies, offering a renewed opportunity for treatment.^842^ In the context of targeted therapy, inhibitors of mutant kinases initially provide rapid benefits but often lead to the development of resistance over time.^843^ The potential of epigenetic treatments, particularly HDAC and DNMT inhibitors, to reverse such resistance is currently being explored.^844,845^ The development of dual inhibitors, like CUDC907, CUDC101, and 4SC-202, shows promising results in overcoming resistance in kinase-driven cancers and warrants further investigation.^846^ For hormone-dependent cancers, such as estrogen receptor-positive breast cancer and androgen receptor-positive prostate cancer, endocrine therapy remains a crucial treatment option.^847^ However, epigenetic alterations can lead to resistance during endocrine therapy.^848,849^ Targeting these epigenetic changes can help sustain the effectiveness of endocrine therapies and reduce the proliferation of cancer cells.^848,850^

At present, various combination regimens based on HMA and traditional anticancer drugs have entered clinical trials, gaining the potential to become an alternative for patients with certain diseases. For example, the combination of the oral B-cell leukemia/lymphoma 2 inhibitor venetoclax with HMAs has become a standard regimen among patients with AML or MDS who are ineligible for intensive chemotherapy. In November 2018, the FDA approved this combination for AML therapy.^851–853^ Further, triplet regimens that include HMAs, venetoclax, and other targeted agents are being developed for AML with specific gene mutations.^229,854–856^ Early results from these clinical trials have demonstrated a good safety profile and promising effects, with ongoing studies needed to confirm their efficacy and potential adverse events. In May 2022, the combination of ivosidenib, an IDH1 inhibitor, and azacitidine was approved by the FDA for older patients with newly diagnosed IDH1-mutated AML.^240^ Other drugs being combined with HMAs include HDAC inhibitors, polo-like kinase 1 inhibitors, T-cell immunoglobulin domain and mucin domain-3 antibodies, and PD-L1 antibodies.^857–860^ These combinations are currently under evaluation in ongoing registrational clinical trials across different stages, with promising results anticipated for updating clinical strategies. Additionally, the combination of HMAs with targeted therapy and immunotherapy, as well as chemotherapy, is showing promising application prospects, especially in hematologic malignancies with acquired chemoresistance caused by aberrant DNA methylation.^861,862^ Currently, azacitidine is approved in multiple countries and is widely used in patients with myeloproliferative disorders, such as MDS, AML, chronic myelomonocytic leukemia (CMML), and juvenile myelomonocytic leukemia, whereas decitabine is approved for the treatment of MDS, AML, and CMML.^863–865^

### Small molecules serving as epigenetics-targeted degraders

As an emerging therapeutic strategy, epigenetics-targeted degraders for targeted protein degradation (TPD), respected by molecules based on PROTAC, autophagy-targeting chimera (AUTAC), hydrophobic tagging (HyT), molecular glue (MG), and other novel techniques for drug discovery are worth trying as a remarkable alternative presenting pioneering approaches.^866^

PROTACs, first proposed in 2001, are considered revolutionary technologies in drug discovery. They consist of a ligand for targeted proteins, a ligand for E3 ubiquitin ligase, such as Von Hippel-Lindau and Cereblon (CRBN), and a linker connecting the two ligands.^867^ Many degraders targeting diverse epigenetic enzymes have been developed, including the newly designed or derived from the optimization of known selective inhibitors.^868,869^ This technology has led to the creation of various degraders targeting a wide range of epigenetic enzymes, enhancing their potency, duration of action, safety profile, and ability to counteract resistance mechanisms compared to conventional inhibitors.^867–869^ For instance, dBET1, derived from the BRD inhibitor JQ-1, was the first PROTAC-based degrader targeting BET proteins. It has demonstrated superior anticancer effects in both AML cell lines and mouse xenograft models compared to JQ-1 alone.^836,870^ Further optimization led to dBET6, which increased cell permeability significantly improved survival rates in solid tumor models, and reduced the emergence of resistance.^836^ Innovative approaches to enhance the selectivity and safety of PROTACs include antibody-PROTAC technology, which combines monoclonal antibodies targeting specific pathological cells with degraders. This strategy facilitates targeted delivery, minimizing side effects while maximizing efficacy. Antibody-PROTACs have been developed for breast cancer cells overexpressing human epidermal growth factor receptor 2 and prostate cancer cells expressing six transmembrane epithelial antigens of the prostate 1, showing enhanced degradation specificity in these cell types.^871,872^ Compared with general degradation agents, these small molecules present the preferential degradation of target proteins in specific cell lines. Additionally, integrating control elements into PROTAC molecules allows for activation in specific physiological or pathological conditions, reducing potential off-target effects.^873^ Techniques such as photocaged PROTACs, photo-switchable PROTACs, and radiotherapy-triggered PROTAC prodrugs represent cutting-edge strategies in this area. These methods ensure that the degradation activity of PROTACs can be spatially and temporally controlled, enhancing their clinical applicability and safety.^874–876^ Overall, the evolution of PROTACs and other PROTACs-oriented TPD strategies is shaping a promising future for epigenetics-targeted therapies, offering more precise and effective treatment modalities for various diseases, particularly cancer.

Other types of epigenetics-targeted degraders, such as AUTAC-based agents, HyT-based degraders, and MG, offer innovative alternatives to PROTACs for TPD. Each technology employs distinct mechanisms and offers unique advantages for therapeutic applications. Unlike PROTACs, which utilize the proteasomal degradation pathway, AUTAC agents promote lysosome-dependent degradation of target proteins.^877^ One of the key benefits of AUTAC degraders is their enhanced membrane permeability due to their typically low molecular weights, making them potent therapeutic candidates.^878^ For instance, AUTAC-based degraders targeting BRD4, developed from the covalent interaction between autophagy key proteins and JQ-1, have shown significant antiproliferative activity across multiple tumor cell lines. This demonstrates their potential as effective medical tools for treating various diseases.^879^ Introduced in 2011, HyT technology uses small molecules composed of a targeted protein ligand, a hydrophobic tag, and a linker. Unlike PROTACs that often target the ubiquitin-proteasome system, HyT-based degraders work by increasing the hydrophobicity of the target protein, facilitating its degradation.^880^ MS1943, an EZH2 HyT degrader, illustrates this technology’s effectiveness.^881^ It has shown superior inhibitory effects on tumor cell lines and greater selectivity towards cancer cells over normal cells, demonstrating significant tumor suppression and good tolerance in mouse models.^881^ Other HyT-based degraders are also developed, including those targeting HDAC and YEATS domain readers, providing therapeutic strategies for diseases caused by mutations or dysfunction in specific proteins.^882–884^ Further development of HyT-based degraders is ongoing, with efforts to improve their bioavailability and therapeutic effects in vivo by exploring new hydrophobic labels.^885^ MG differs fundamentally from PROTACs and other degraders by inducing degradation through promoting tight binding between the target protein and proteasome components, leading to the protein’s subsequent degradation.^886,887^ A notable example is the MG-based degrader DD-1-073, targeting HDAC1/3, derived from SAHA (a known HDAC inhibitor). This was among the first applications of MG in HDAC degrader development.^888^ Similarly, another MG-based agent targeting BRD4, termed JP-2-197, is further established as an optimal derivative of JQ-1.^888^ Due to their low molecular weights, DD-1-073 and JP-2-197 have favorable pharmacokinetic properties, enhancing cell permeability and drug-ability.^888^ Despite their potential, the development of MG-based agents faces challenges due to the lack of systematic strategies for their design and identification, making large-scale screening and optimization difficult.

Developing epigenetic-targeting degraders, especially those targeting HDACs and epigenetic readers, has made significant progress, opening new avenues for clinical practice. These novel small molecules offer promising therapeutic alternatives, but several challenges and limitations must be addressed to enhance their clinical applicability and effectiveness.

### Combining epigenetics-targeted drugs and sequencing technology

Owing to the substantial relationship among epigenetic signatures, lifestyle choices, and environmental influences, drugs that target epigenetic mechanisms are highly promising for advancing personalized medicine.^889^ However, leveraging these drugs in this field is challenging. As disease research enters a new phase owing to advancements in sequencing technologies, the potential for epigenetic therapies to enhance personalized healthcare is being progressively realized.^890^

The advent of these cutting-edge technologies, ranging from whole-genome sequencing to single-cell analysis, has facilitated the detection of gene mutations and expression alterations associated with epigenetic changes throughout disease progression.^891^ This advancement significantly enhances our understanding of the heterogeneity in epigenetic modifications across various cell types, thereby revealing new therapeutic targets for clinical application. In recent years, advancements in single-cell methodologies have allowed researchers to further explore the multiple dimensions of the epigenome, including chromatin accessibility, DNA methylation patterns, histone modification profiles, and chromatin interaction networks.^892–894^ Collectively known as “single-cell epigenomics”, this burgeoning field offers an enhanced comprehension of epigenomic regulation in physiological and pathological settings at the level of individual cells from a more intuitive perspective.^895–897^ These comprehensive “omic” profilings support the distinct biological identity of individuals, thus providing a solid theoretical foundation for refining therapeutic strategies to achieve individual targeting.^898^ Furthermore, by integrating CRISPR/cas9 gene-editing technology with various sequencing, researchers can conduct high-throughput functional genomic screens and identify pathological genes that are responsive to epigenetic therapies. This step not only aids in identifying novel targets for epigenetic agents but also promotes the assessment of therapeutic responses of target tissues or cells.^899,900^ For example, these methods may help detect the occurrence of drug resistance and optimize the efficacy of epigenetic interventions.^901^

The significant potential of epigenetics in customizing personalized medicine has generated high enthusiasm, and advancements in this field have been consistently focused in medical research. Notably, the introduction of sequencing technologies has enabled us to investigate the correlation between epigenetic markers and disease pathology more comprehensively, thus facilitating the development of targeted therapeutic strategies. Nonetheless, the current epigenetic technologies used in the laboratory present several technical challenges that require refinement, such as the necessity for high-demand algorithms,^902^ sufficient amounts of training data,^903^ limited genome coverage per cell,^904,905^ and uncertain reproducibility.^906^ These techniques require significant improvement before they can be effectively used in clinical practice.

## Conclusions and perspectives

Since the term “epigenetics” was first introduced in 1942, there has been a significant focus on elucidating the mechanisms and pivotal roles of epigenetic modifications and their associated enzymes in human physiology and pathology. Drugs targeting epigenetic enzymes have shown promising potential for treating diseases, particularly cancers. This review comprehensively examines the major epigenetic mechanisms involved in the pathogenesis and progression of various diseases. It also highlights recent advances in epigenetics-targeted drugs, underscoring their potential in clinical settings. Additionally, we explore the integration of novel technologies in drug development and the synergistic value of these drugs in conjunction with other cancer therapies, pointing to the future direction of epigenetics-oriented therapeutic strategies. Despite these advancements, significant challenges remain. For instance, certain enzymes that regulate the epigenetic landscape still lack effective targeted drugs, and those identified through virtual screening require further in vivo and in vitro investigation to validate their efficacy and safety profiles. Addressing these gaps will be crucial for integrating epigenetics-targeted drugs into clinical practice.

Advances in in-depth understanding of epigenetics have largely enhanced possibilities of curing disease. To date, the application of epigenetics-modifying drugs in preclinical and clinical setting has provided promise for the beginning of the era of epigenetic-orientated therapeutic strategies. Given the future needs in this field, great attention should be focused on exploring the heterogeneity of epigenetic hallmarks in different diseases to design and develop epigenetics-targeted drugs with high selectivity and improved targeting efficiency, based on the elucidation for the biological and pathological roles of epigenetics. These results are imperative for designing and developing agonists and inhibitors of epigenetic enzymes with enhanced selectivity and bioactivity. Furthermore, apart from the knowledge based on experimental research and preclinical studies, assessing the therapeutic potential of epigenetic drugs for specific diseases, particularly in advanced clinical trials, is also vital for advancing this field. Concurrently, emphasis should be placed on the clinical potential of integrating innovative drug discovery technologies into developing epigenetic-based drugs. Moreover, while capitalizing on unique strengths of epigenetics, efforts should be made to combine these novel agents with traditional therapeutic modalities, with a view to achieving synergic effects in treating disease, especially in the case of tumors with genomic complexity. In conclusion, we believe that deepened research in this field will catalyze innovation in treatment approaches for diseases involving epigenetic mechanisms, offering new hope to patients.
